# Supplementary material for: Multiplexed and high-throughput neuronal fluorescence imaging with diffusible probes
Source: Nat Commun. 2019 Sep 26;10:4377. doi: 10.1038/s41467-019-12372-6 (PMC6763432; doi:10.1038/s41467-019-12372-6)
Supplement: Supplementary file 1 — Supplementary Information [file 41467_2019_12372_MOESM1_ESM.pdf]

Supplementary Information

Multiplexed and high-throughput neuronal fluorescence imaging with diffusible probes

Guo *et al.*

Supplementary Note 1

To identify high quality antibodies for PRISM, we first used CiteAb to identify antibodies with high citation numbers in the literature, and subsequently performed IF using these antibodies to characterize their staining patterns (e.g., linear structures for cytoskeletal antibodies, colocalization with other synaptic markers for synaptic antibodies). In addition, we performed cross-correlation analysis (see **Supplementary Figure 1**) to quantify the extent of colocalization of the synaptic antibodies with validated synaptic marker synapsin-I. Specifically, we first identified two synapsin-I antibodies that exhibited highly similar staining patterns (Pearson correlation coefficient, PCC=0.9) and used them as synaptic markers. We then performed co-staining of the antibody being tested with the synapsin-I antibody. For each co-stained image, the spatial cross-correlation function (SCCF) between the two channels was calculated. The correlation decay length and the zero-shift correlation (equivalent to the PCC) was extracted from the SCCF. Higher PCC and smaller correlation decay length indicate higher colocalization with the canonical synaptic marker and therefore greater antibody quality indicated by staining specificity.

Supplementary Note 2

For independent channel multiplexed imaging, it is essential to ensure that each diffusible nucleic acid imaging probe binds exclusively to its corresponding complementary docking strand on the target antibody, with zero-to-minimal cross-hybridization with off-target docking strands, as well as non-specific binding to cells. Non-specific binding of imaging probes to cells will result in background fluorescence in images. Cross-hybridization of imaging probes with docking strands will result in bleed-through of the signal from one channel to another, similar to the bleed-through or crosstalk due to the overlap of spectra in conventional fluorescence microscopy. However, the cross-hybridization level between two docking sequences A and B depends on a number of factors, including the densities of each docking strand in the sample and the melting temperatures of the complementary duplexes (A-A' and B-B') and non-complementary duplexes (A-B' and B-A'), which may vary across cellular systems and plating conditions. Thus, for any given cellular application it is crucial to characterize cross-hybridization *in situ* (see **Supplementary Figure 11**) within the same cell culture that is used for multi-channel imaging (e.g., primary neuronal culture in multi-well plate format).

Supplementary Note 3

The nanometer-scale resolution achieved by DNA-PRISM imaging and downstream protein co-localization analyses can be highly sensitive to physical sample perturbation. The requirement for multiple rounds of gentle reagent exchange and wash-out needed for multiplexing therefore make manual handling infeasible. To reliably control fluid pressure over the sample and automate multiple rounds of imaging probe wash-out and exchange for PAINT-based imaging, we designed a simple flow chamber setup in a multi-well plate format (**Supplementary Figure**). Using this setup, neurons were cultured and stained with a standard protocol in a 96-well plate (see Materials and Methods), with individual wells converted into flow cells prior to imaging directly in the multi-well plate. The flow of imaging probe and wash buffer was regulated using a custom software-controlled fluidics system. This setup streamlined the multiplexed imaging workflow and minimized physical perturbation of subcellular structures.

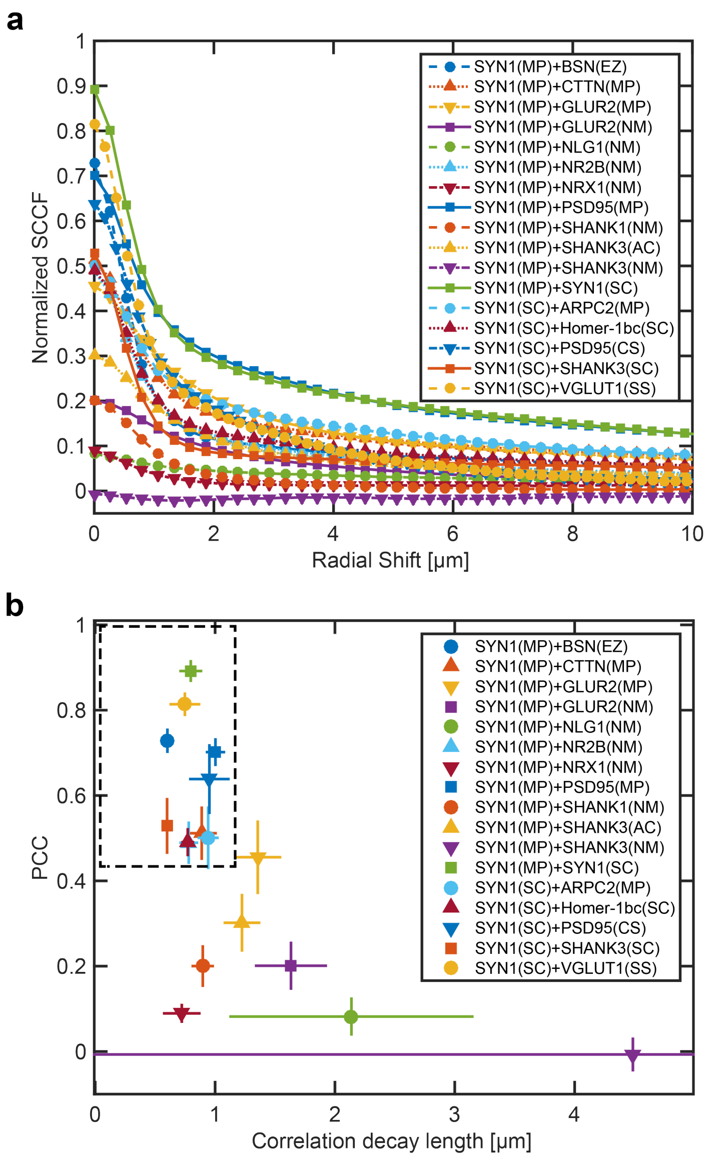

Supplementary Figure 1. Image cross-correlation analysis for antibody evaluation prior to ssDNA conjugation. (a) Spatial cross-correlation function (SCCF) between the confocal images of the antibody being tested and the synaptic marker synapsin-I. (b) The average Pearson correlation coefficient (SCCF at zero shift) versus the correlation decay length of the SCCF for each antibody tested in (a). Higher PCC and smaller correlation decay length indicate better colocalization of the antibody with the synaptic marker. Only antibodies within the boxed region were used in PRISM. Error bars represent 95% confidence intervals. Antibody target names and antibody vendor: synapsin-I (SYN1), bassoon (BSN), cortactin (CTTN), Neuroligin 1 (NLG1), NMDAR2B (NR2B), Neurexin 1 (NRX1); Millipore (MP), Santa Cruz (SC), Enzo Life Sciences (EZ), NeuroMab (NM), Abcam (AC), Cell Signaling (CS), Synaptic Systems (SS).

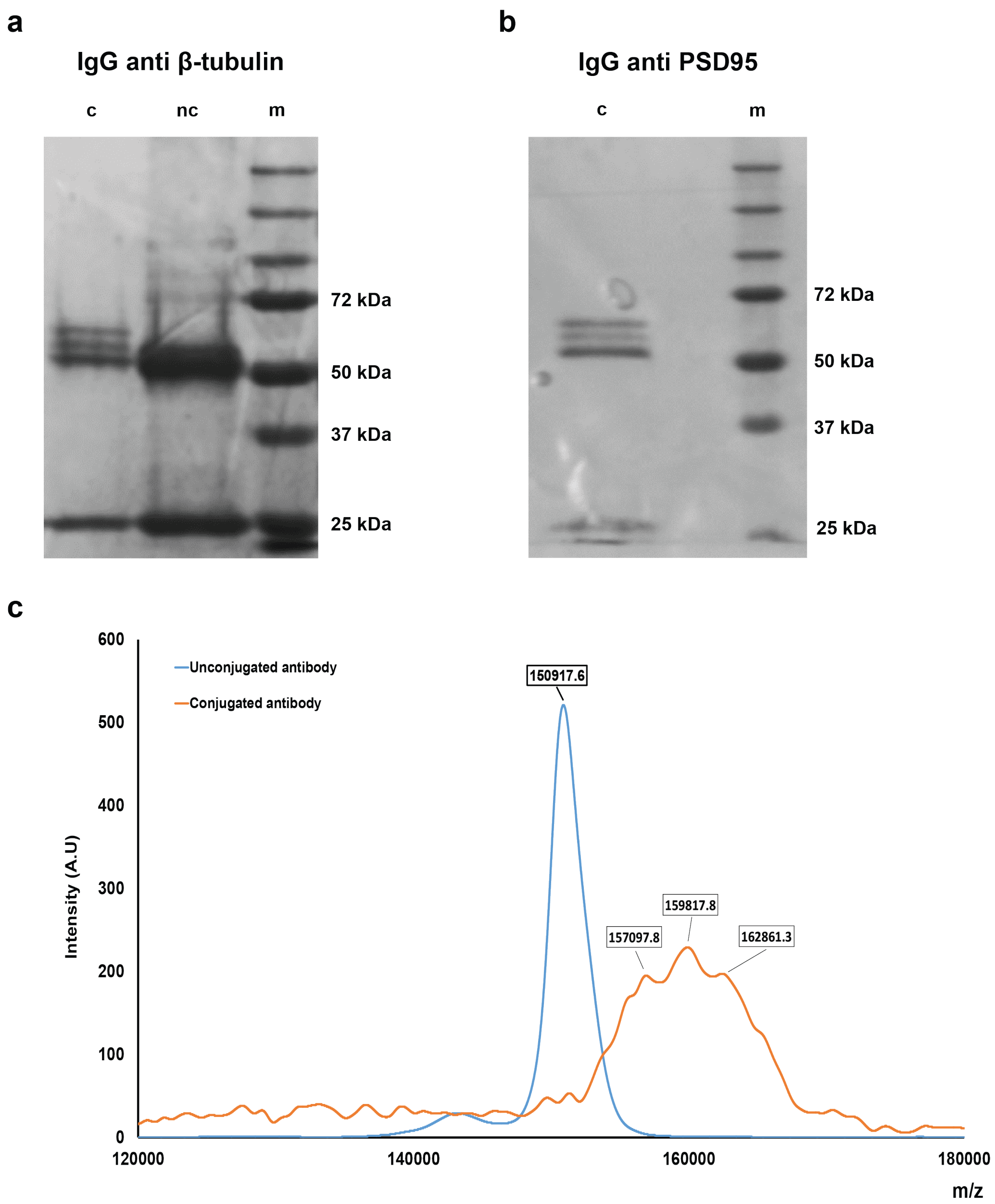

Supplementary Figure 2. SiteClick specifically conjugates 2-4 ssDNA docking strands to the heavy chain of the antibody. (a and b) SDS-PAGE electrophoresis of native or ssDNA-conjugated IgG antibodies using SiteClick against (a) β-tubulin III and (b) PSD95. Antibodies were reduced by DTT before electrophoresis to separate the heavy and light chains (c: conjugated, nc, native, m: protein marker). SDS-PAGE shows only the heavy chains of the antibodies are modified. (c) Mass spectra of the native or ssDNA-conjugated IgG. Three peaks in the ssDNA-conjugated IgG curve (orange) correspond to masses of IgG with 2, 3, and 4 ssDNA oligos.

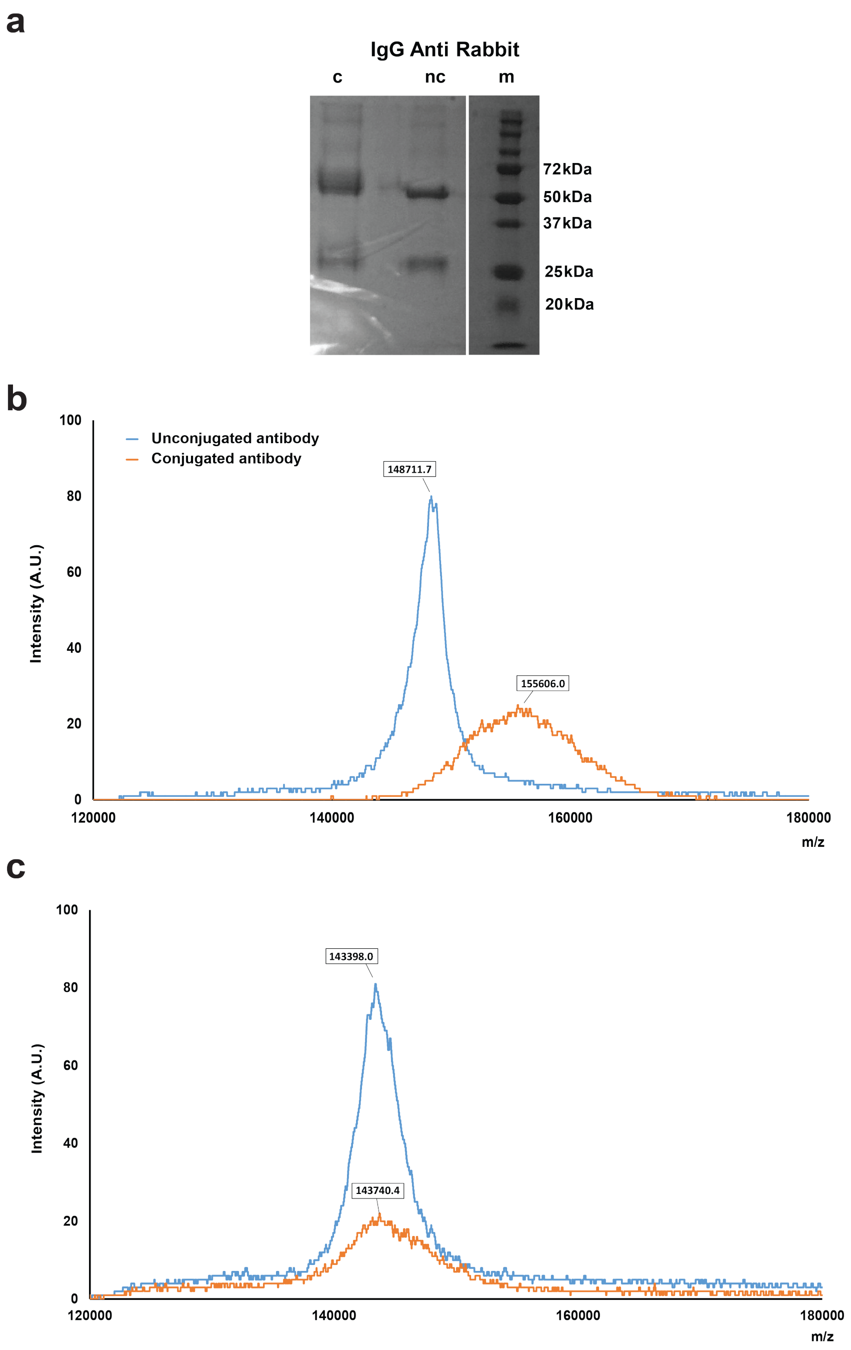

Supplementary Figure 3. SMCC modification of antibodies. (a) SDS-PAGE electrophoresis of native or ssDNA-conjugated, anti-rabbit secondary IgG antibodies. Antibody was reduced by DTT before electrophoresis to separate the heavy and light chains (c: conjugated, nc, native, m: protein marker). SDS-PAGE shows both the heavy and light chains of the antibodies are modified. (b) Mass spectra of the native or ssDNA-conjugated anti-bassoon IgG. (c) Mass spectrum of the native or ssDNA-conjugated anti-SHANK3 IgG.

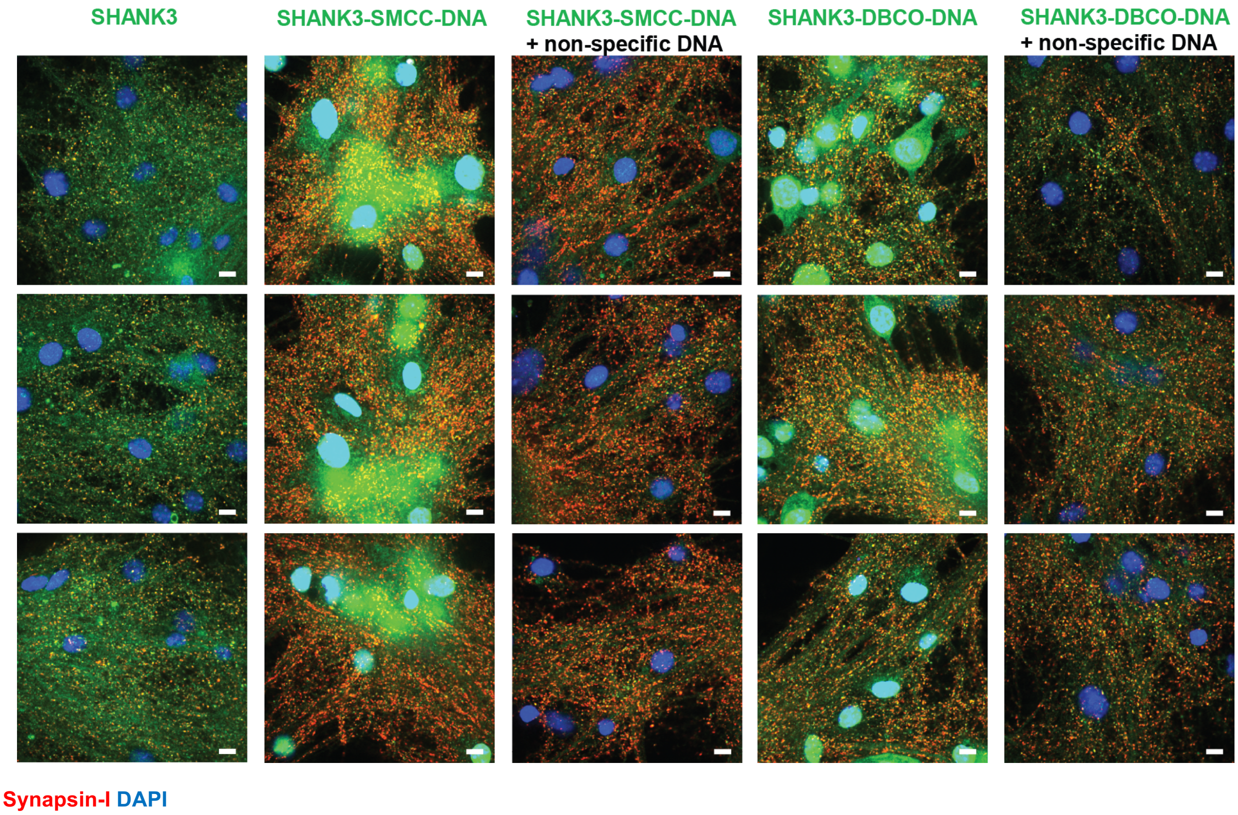

Supplementary Figure 4. IF images show that non-specific (salmon sperm) DNA blocks off-target localization of ssDNA-conjugated antibody to nuclei (DAPI stain shown in blue). Cortical neurons were stained either with native or ssDNA-conjugated anti-SHANK3 antibody (green) together with anti-synapsin-I antibody (red). Three fields of view (image panel rows) are shown for each staining condition (image panel column). Scale bars: 10 μm.

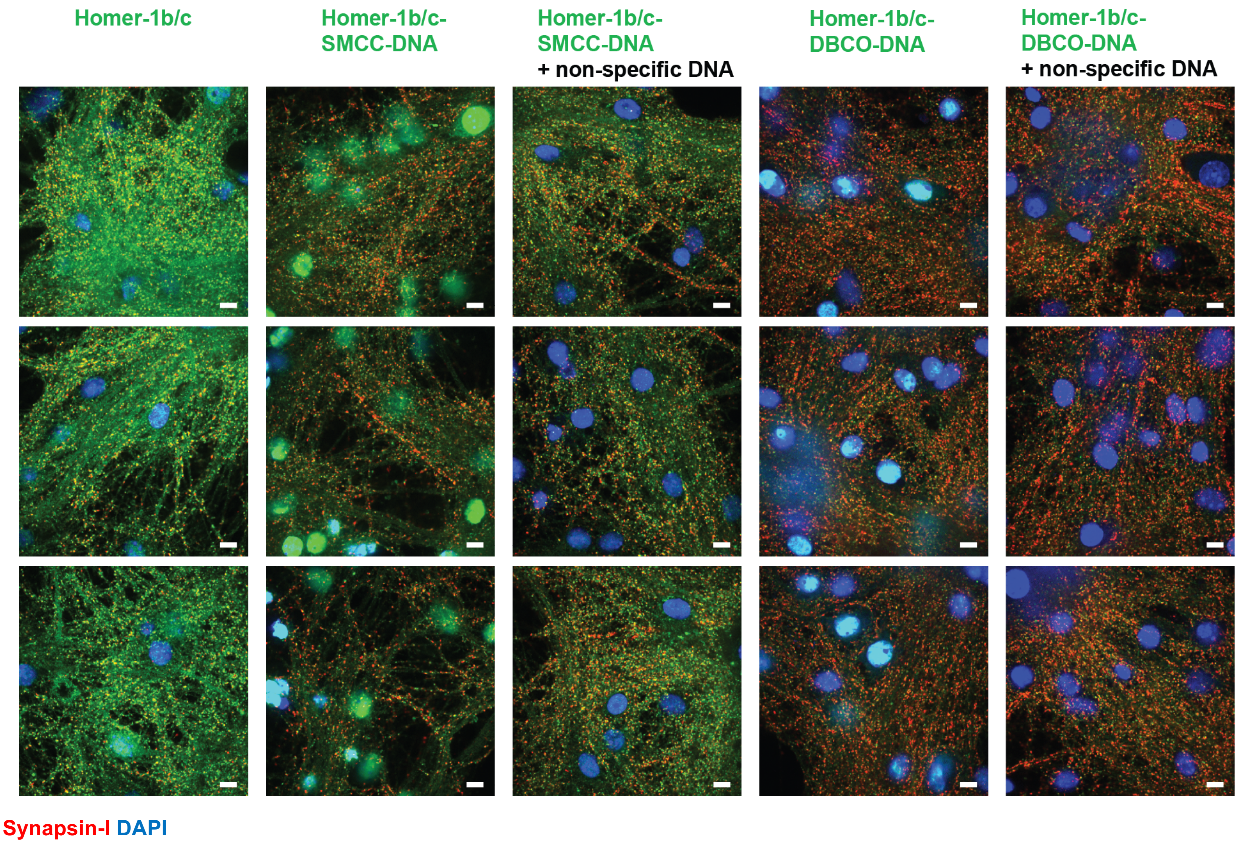

Supplementary Figure 5. IF images show that non-specific (salmon sperm) DNA blocks off-target localization of ssDNA-conjugated antibody to nuclei (DAPI stain shown in blue). Cortical neurons were stained either with native or ssDNA-conjugated anti-Homer-1b/c antibody (green signal in images) together with anti-synapsin-I antibody (red signal in images). Three fields of view (image panel rows) are shown for each staining condition (image panel column). Scale bars: 10 μm.

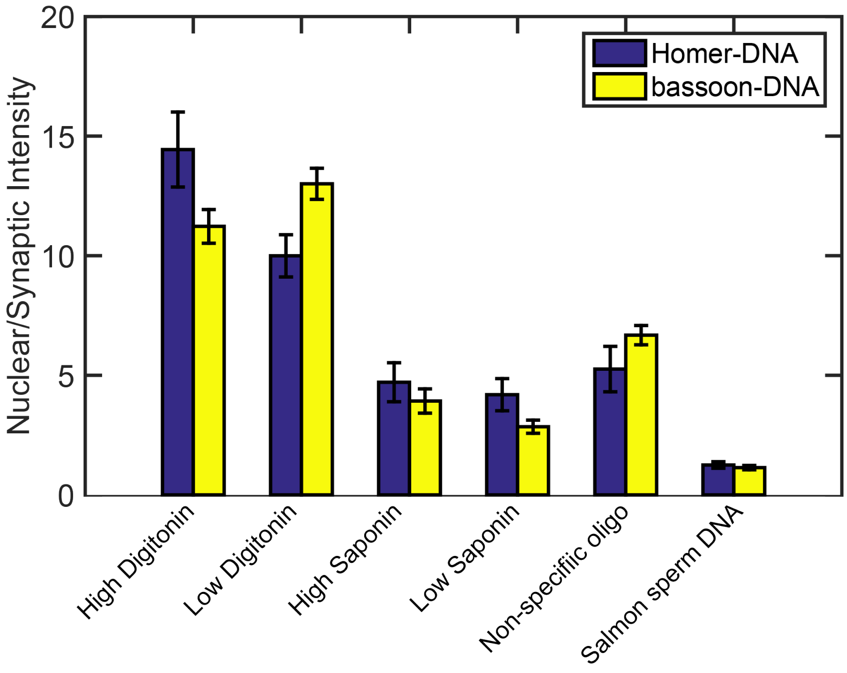

Supplementary Figure 6. High-content screening for nuclear blocking agents. Six nuclear blocking conditions were tested: saponin and digitonin, which are mild detergents that have been shown to selectively permeablize the plasma membrane, but not the nuclear envelope, at optimal concentrations; short non-specific ssDNA oligo (10 nt) that has similar size as the ssDNA conjugated to the antibodies; salmon sperm DNA, which is commonly used to block non-specific binding of ssDNA in Southern blotting. Two ssDNA-conjugated antibodies against synaptic proteins Homer-1b/c and bassoon were tested for each condition. Screening results showed that cells blocked with salmon sperm DNA exhibit nuclear/synaptic intensity ratio close to 1. Error bars represent 95% confidence intervals.

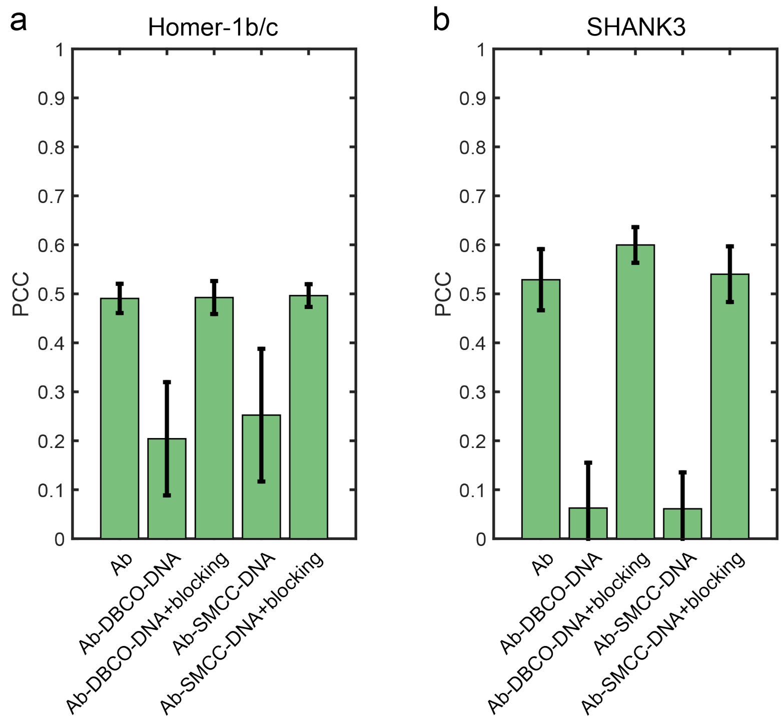

Supplementary Figure 7. Validation of ssDNA-conjugated antibodies using cross-correlation analysis. PCCs for (a) Homer-1b/c and (b) SHANK3 images shown in **Supplementary Figure 4** and **Supplementary Figure 5** were used to characterize changes in IF staining patterns. Reduced PCC of ssDNA-conjugated antibodies indicates disruption in antibody staining patterns (lower colocalization with synapsin-I). Blocking cells with salmon sperm DNA reverses the disruption in staining patterns. Error bars represent 95% confidence intervals.

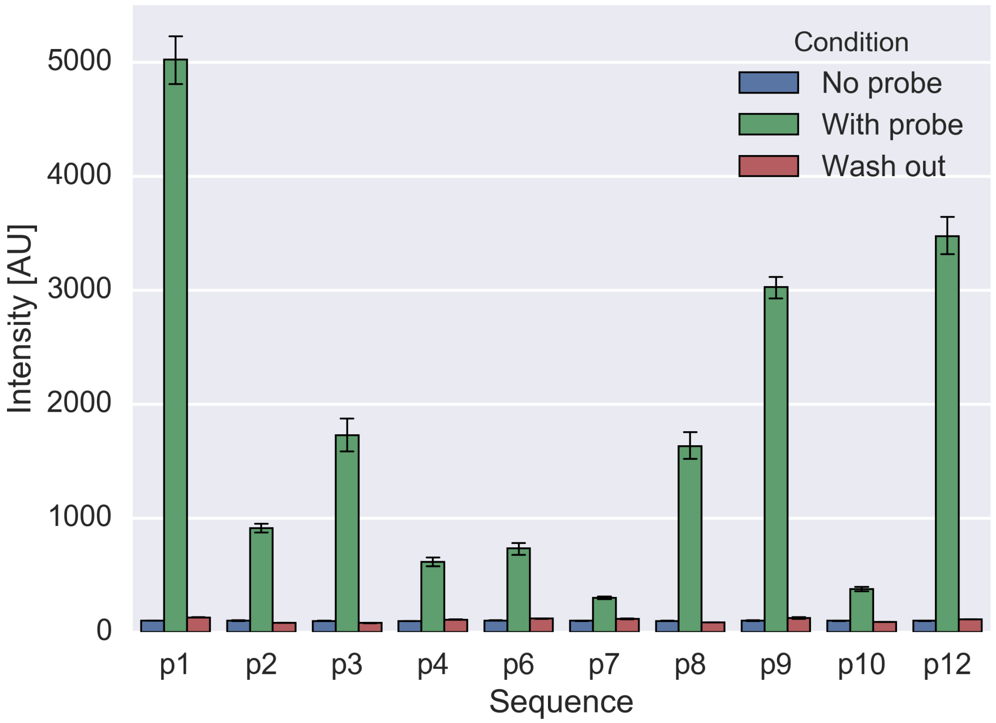

Supplementary Figure 8. Reversible staining with ssLNA imaging probes. Bar plot showing the average intensity within neuronal synapses for immunostained targets shown in **Supplementary Table 1** in three conditions: before incubation with ssLNA probe (blue), after 5 minute incubation with the ssLNA probe (green), or after 5 minute wash-out of the probe using probe-free low-salt buffer (0.01X PBS) (red). Six fields of view were averaged for each condition. Error bars represent 95% confidence intervals.

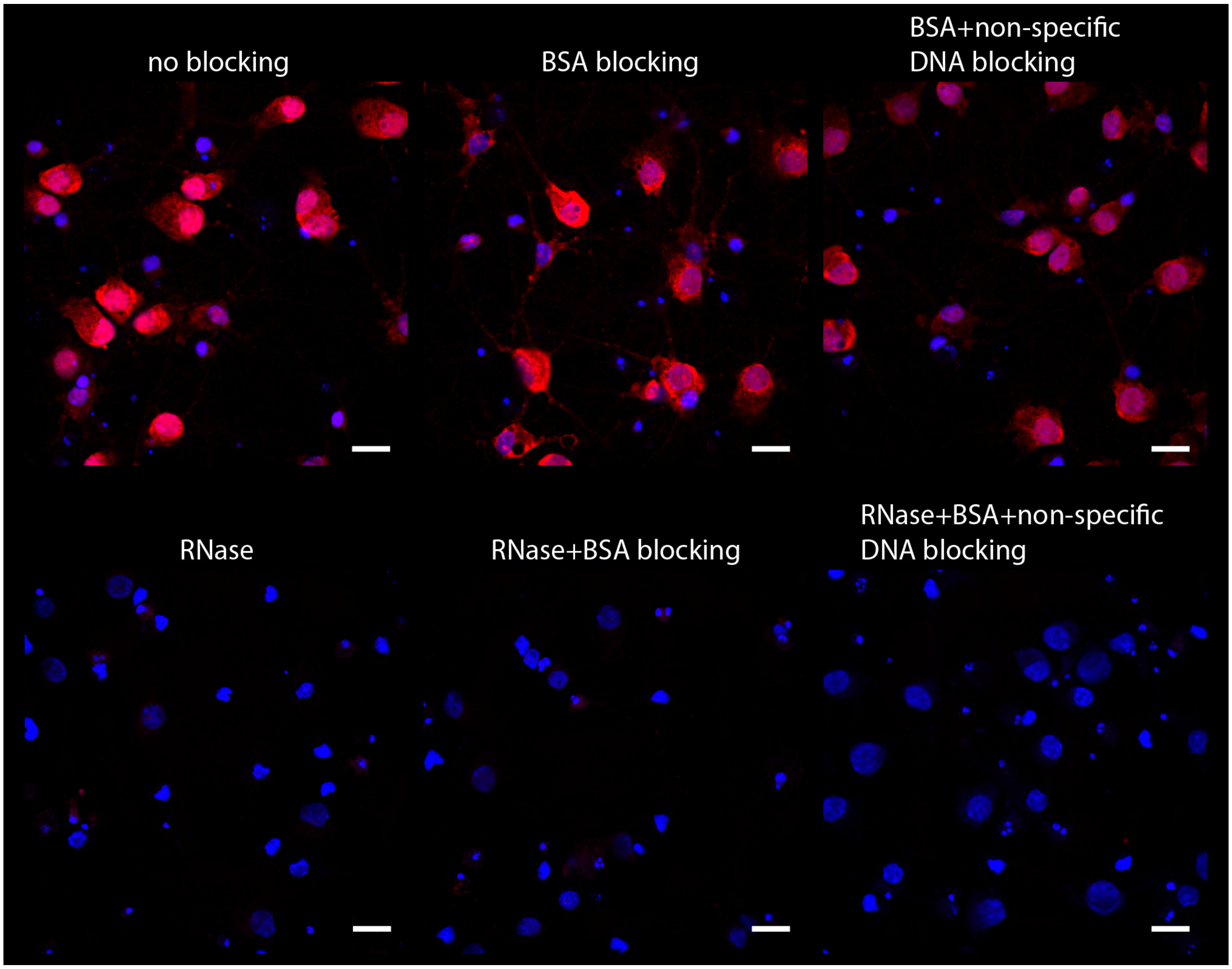

Supplementary Figure 9. RNase treatment reduces the non-specific binding of ssLNA imaging probes in neuronal culture. Fixed neuronal cultures treated with different blocking reagents in the absense of any antibody were incubated with p10 ssLNA probe (red fluorescence signal in the images). Cells treated with RNase (second row images) showed significantly lower non-specific binding due to the enzymatic digestion of endogenous RNA. All images were acquired using the same imaging settings and shown at the same contrast level. Blue in the images corresponds to Hoechst staining. BSA, bovine serum albumin. Scale bars: 20 μm.

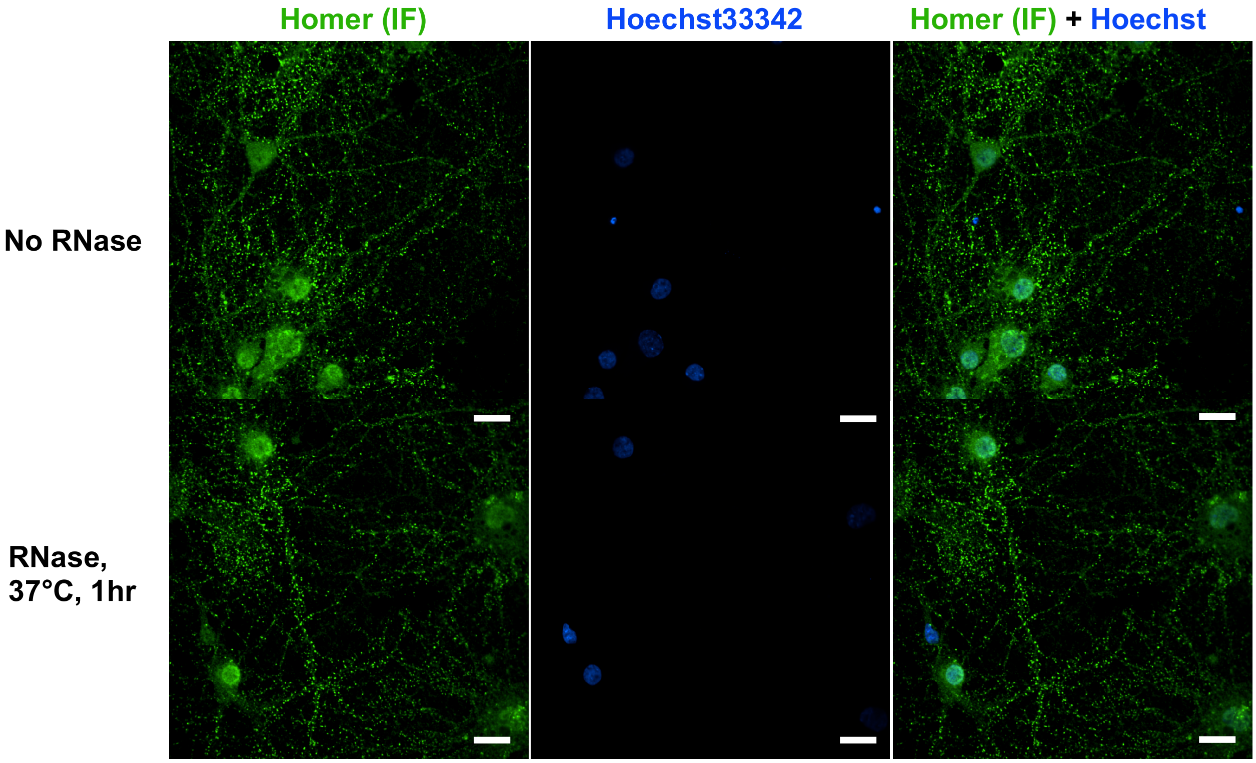

Supplementary Figure 10. Binding of antibodies to their targets is unaffected by RNase treatment. Neurons were treated with or without RNase (image rows) and stained with anti-Homer-1b/c primary antibody and fluorophore-conjugated secondary antibody. Neurons treated with RNase did not show any apparent loss of Homer-1b/c signal. Scale bars: 20 μm.

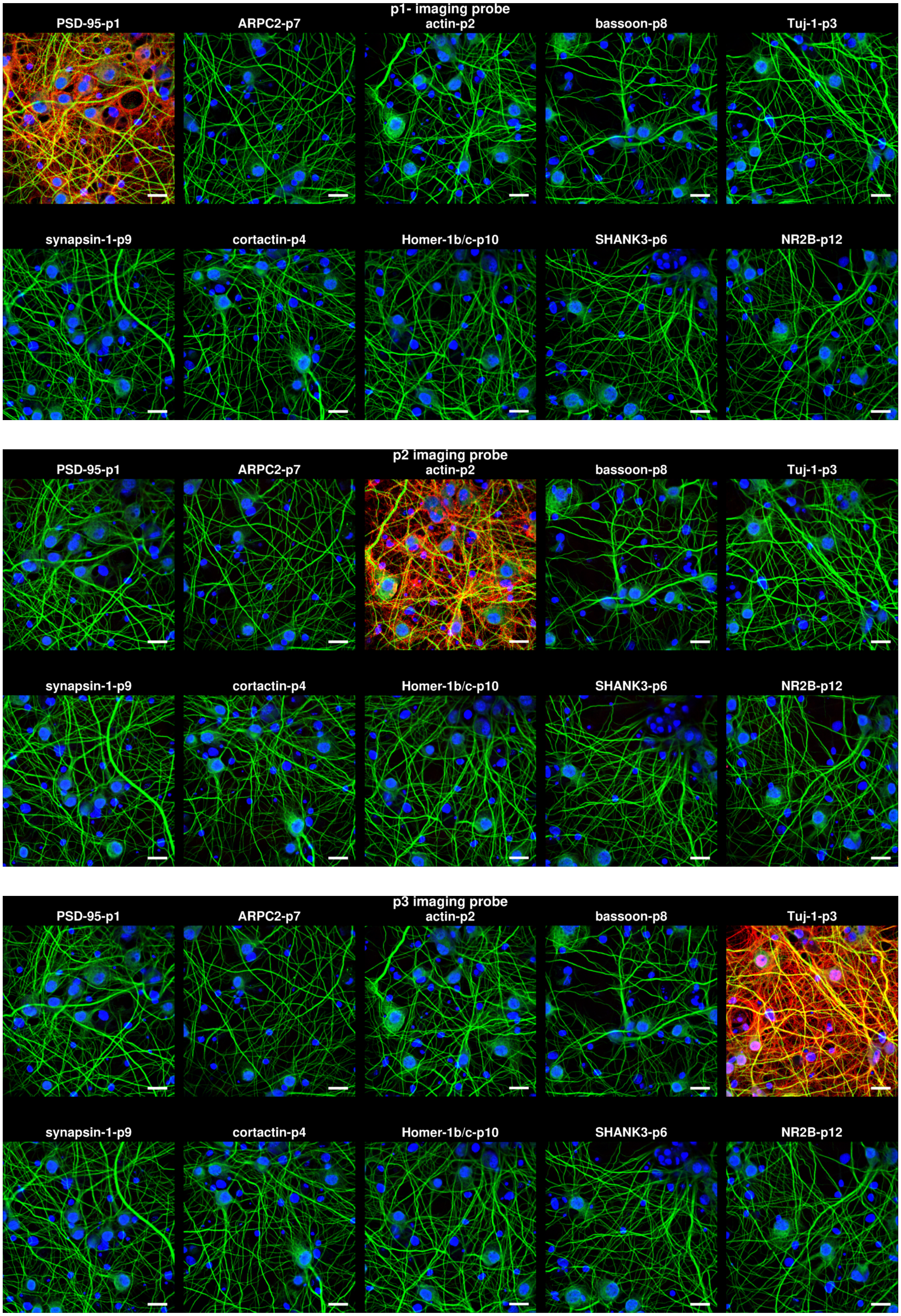
p1- imaging probe
PSD-95-p1
ARPC2-p7
actin-p2
bassoon-p8
Tuj-1-p3
synapsin-1-p9
cortactin-p4
Homer-1b/c-p10
SHANK3-p6
NR2B-p12
p2 imaging probe
PSD-95-p1
ARPC2-p7
actin-p2
bassoon-p8
Tuj-1-p3
synapsin-1-p9
cortactin-p4
Homer-1b/c-p10
SHANK3-p6
NR2B-p12
p3 imaging probe
PSD-95-p1
ARPC2-p7
actin-p2
bassoon-p8
Tuj-1-p3
synapsin-1-p9
cortactin-p4
Homer-1b/c-p10
SHANK3-p6
NR2B-p12

(Continued)

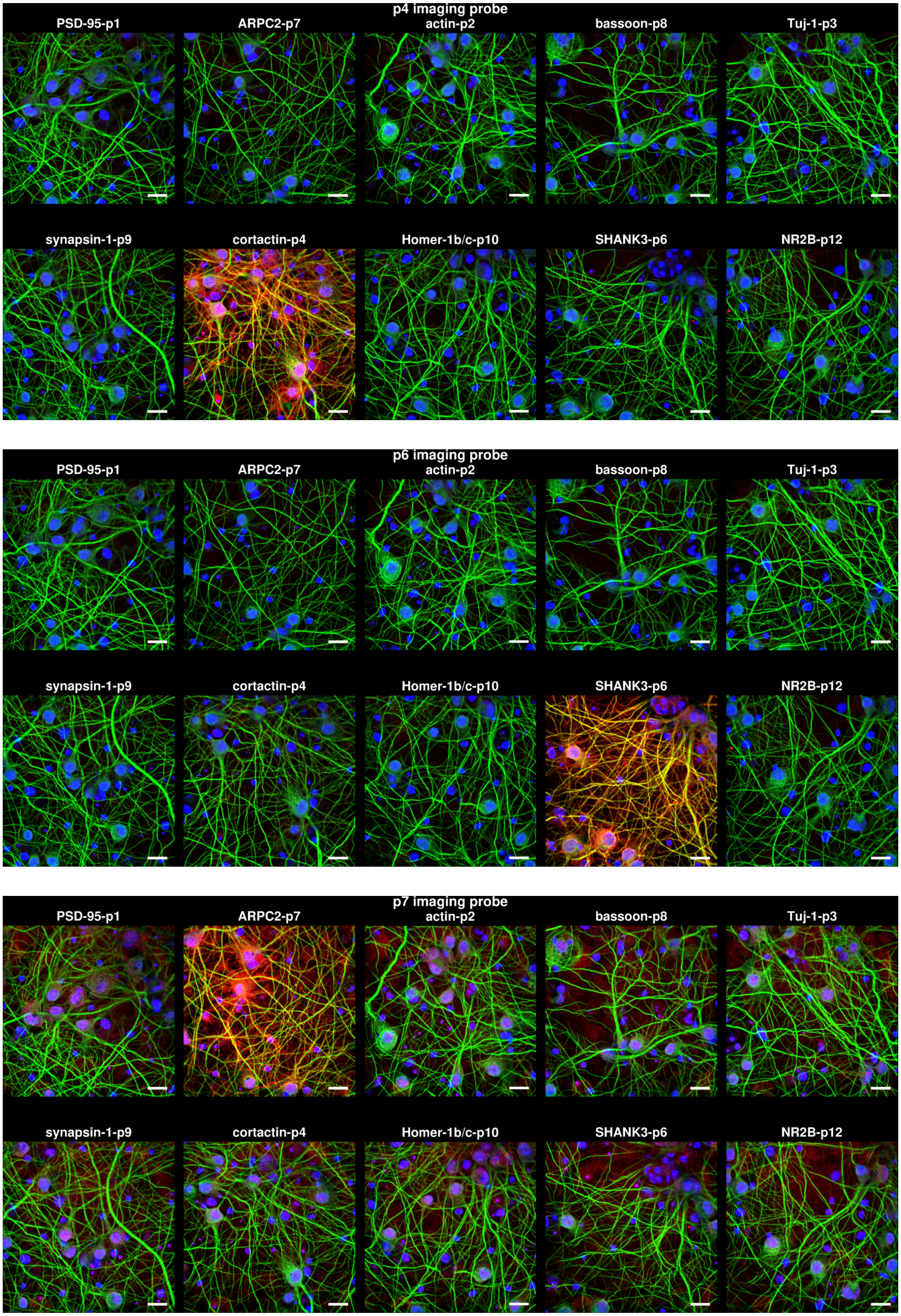
p4 imaging probe
PSD-95-p1
ARPC2-p7
actin-p2
bassoon-p8
Tuj-1-p3
synapsin-1-p9
cortactin-p4
Homer-1b/c-p10
SHANK3-p6
NR2B-p12
p6 imaging probe
PSD-95-p1
ARPC2-p7
actin-p2
bassoon-p8
Tuj-1-p3
synapsin-1-p9
cortactin-p4
Homer-1b/c-p10
SHANK3-p6
NR2B-p12
p7 imaging probe
PSD-95-p1
ARPC2-p7
actin-p2
bassoon-p8
Tuj-1-p3
synapsin-1-p9
cortactin-p4
Homer-1b/c-p10
SHANK3-p6
NR2B-p12

(Continued)

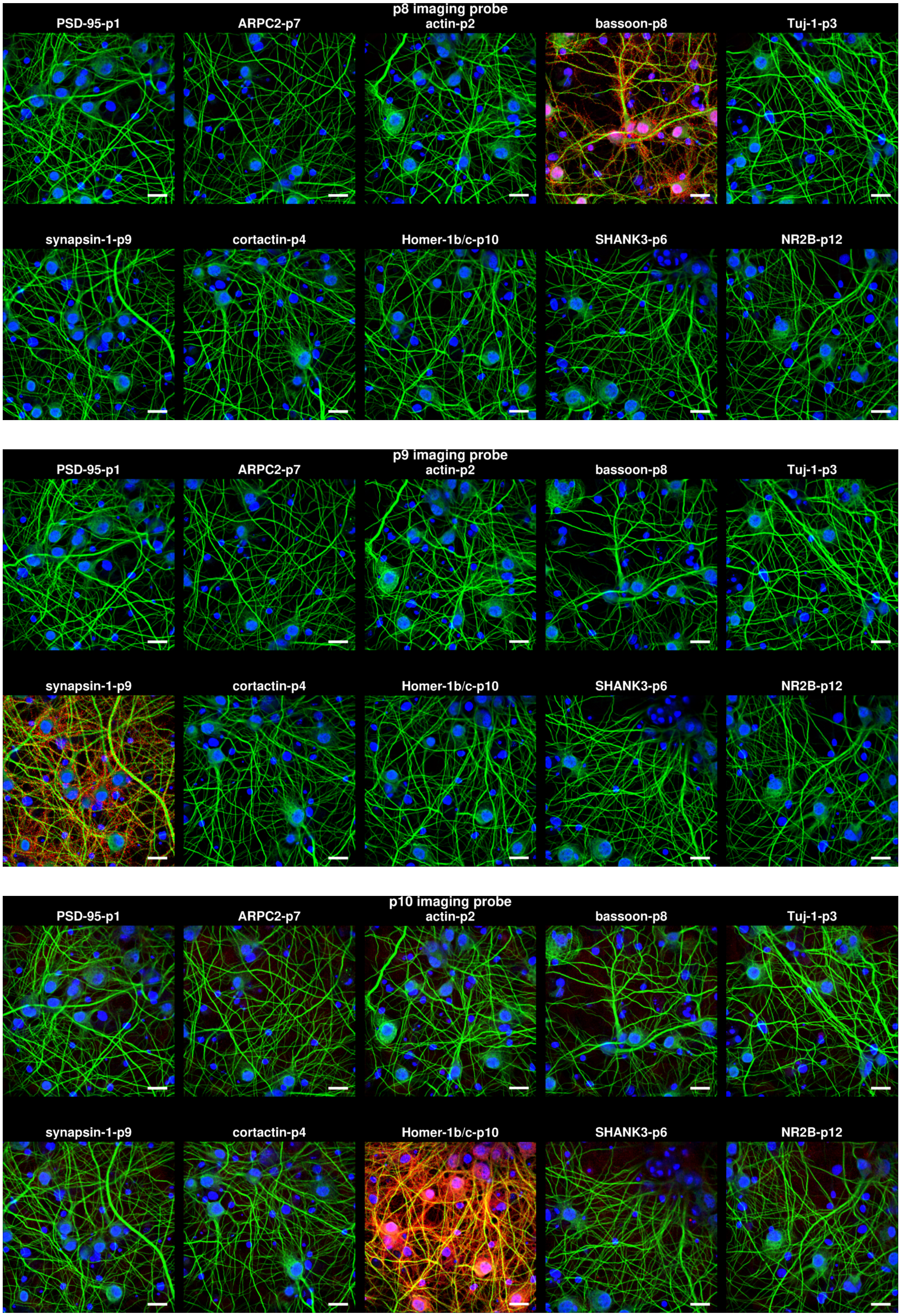
p8 imaging probe
PSD-95-p1
ARPC2-p7
actin-p2
bassoon-p8
Tuj-1-p3
synapsin-1-p9
cortactin-p4
Homer-1b/c-p10
SHANK3-p6
NR2B-p12
p9 imaging probe
PSD-95-p1
ARPC2-p7
actin-p2
bassoon-p8
Tuj-1-p3
synapsin-1-p9
cortactin-p4
Homer-1b/c-p10
SHANK3-p6
NR2B-p12
p10 imaging probe
PSD-95-p1
ARPC2-p7
actin-p2
bassoon-p8
Tuj-1-p3
synapsin-1-p9
cortactin-p4
Homer-1b/c-p10
SHANK3-p6
NR2B-p12

(Continued)

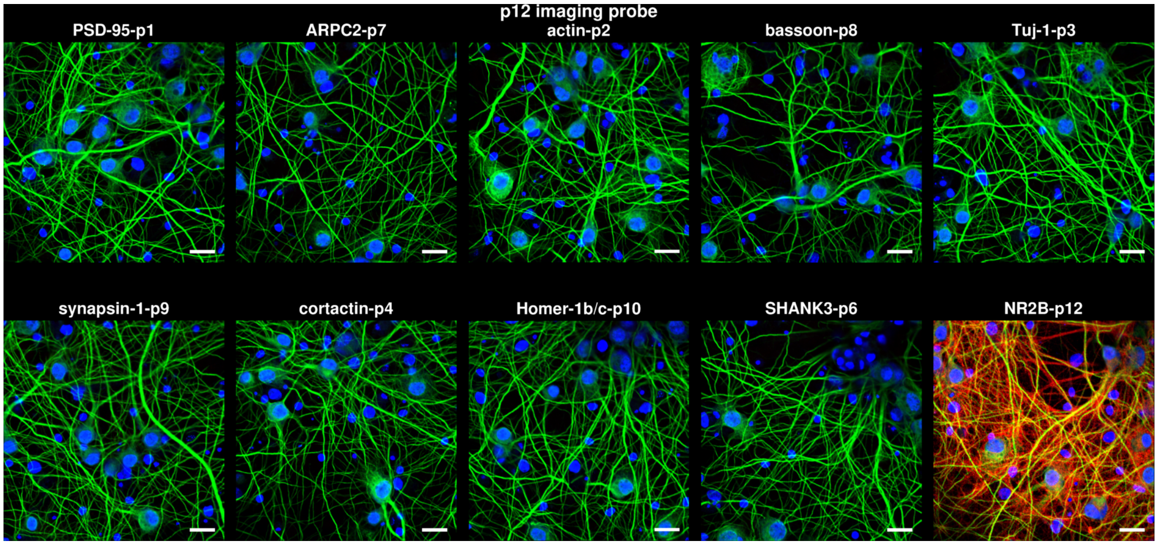

Supplementary Figure 11. Characterization of the specificities of 10 docking strand-to-imaging probe pairs in neuronal culture. Independent neuronal cultures in each of ten wells were stained with a distinct ssDNA-conjugated antibody targeting a single protein species. Each of the 10 wells was then incubated with a single ssLNA imaging probe at a time, imaged, washed, and so on for each of the ten imaging probes. Ideal imaging probes would produce high fluorescence in the well with the matching docking strand sequence, and zero-to-low fluorescence in other wells. Images from two rounds of ssLNA incubation (p9 and p12) are shown (red: ssLNA imaging probe, blue: Hoechst). G-a-R (Goat anti-Rabbit secondary antibody); G-a-Ms (Goat anti-Mouse secondary antibody). Scale bars: 20 μm.

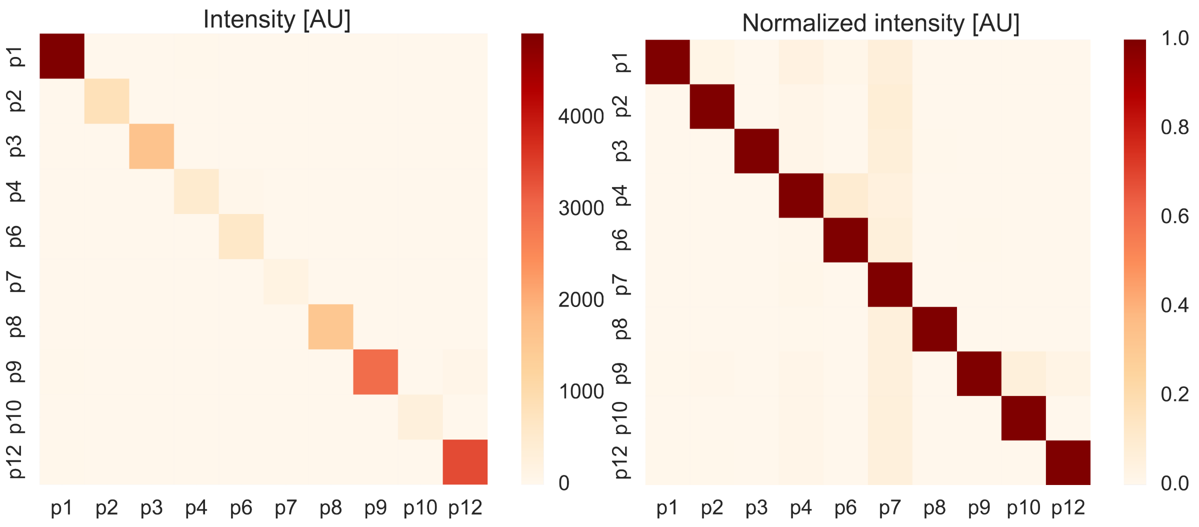

Supplementary Figure 12. Quantification of the ssLNA probe specificity test shown in **Supplementary Figure 11**. Heat map shows (left) the average fluorescence intensity in the synaptic regions for each combination of the imaging probe (column) with the docking strand (row) and (right) the normalized fluorescence intensity. Normalization is performed by dividing each column by its diagonal value. The off-diagonal part of the normalized intensity heat map corresponds to the ratio of (crosstalk+background)/signal for each docking strand-to-imaging probe pair in the PRISM images.

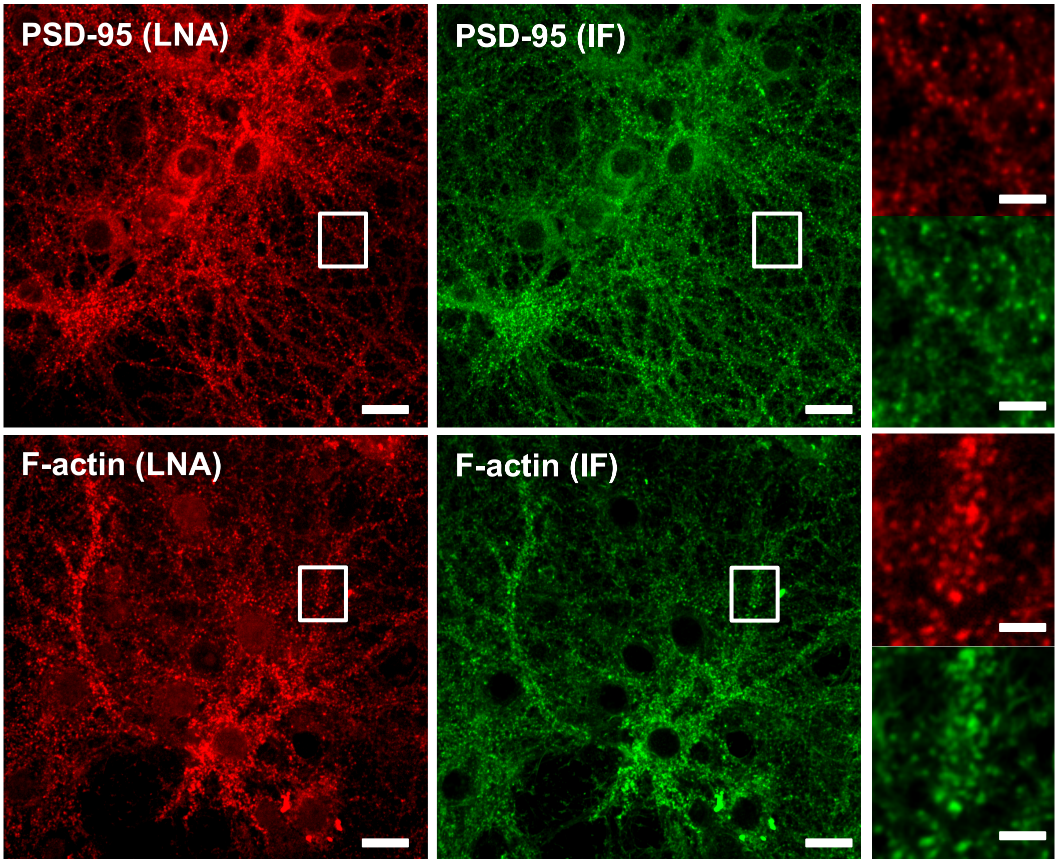

Supplementary Figure 13. Validation of LNA-PRISM. Rat hippocampal neurons stained with ssDNA-conjugated primary antibody and phalloidin against PSD-95 and F-actin were visualized using ssLNA (red) and fluorescently-labeled secondary antibodies (green). ssLNA produced the same staining patterns as regular fluorescently-labeled secondary antibodies. Zoom-in view of the boxed regions are shown on the right. Scale bars: 20 μm in the full fields of view and 5 μm in the zoom-in images.

Dendrite 1

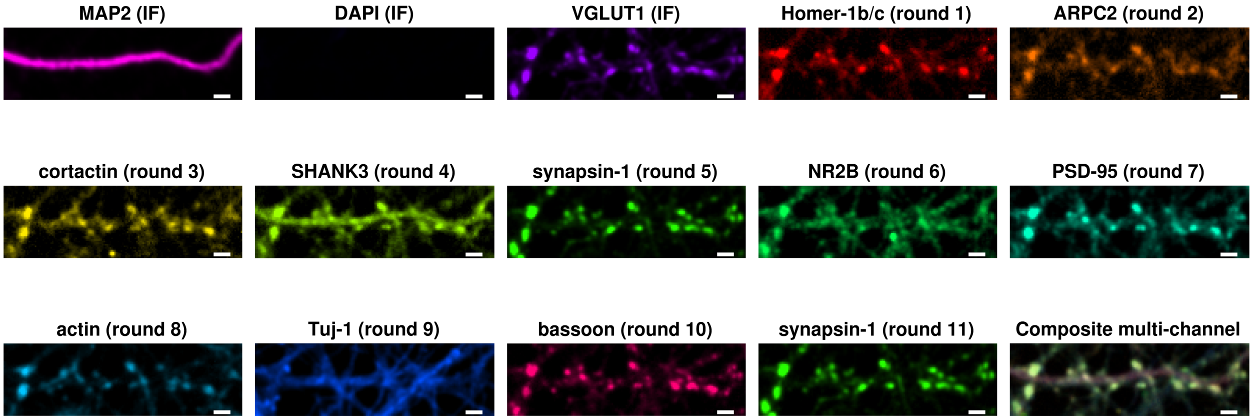

Dendrite 2

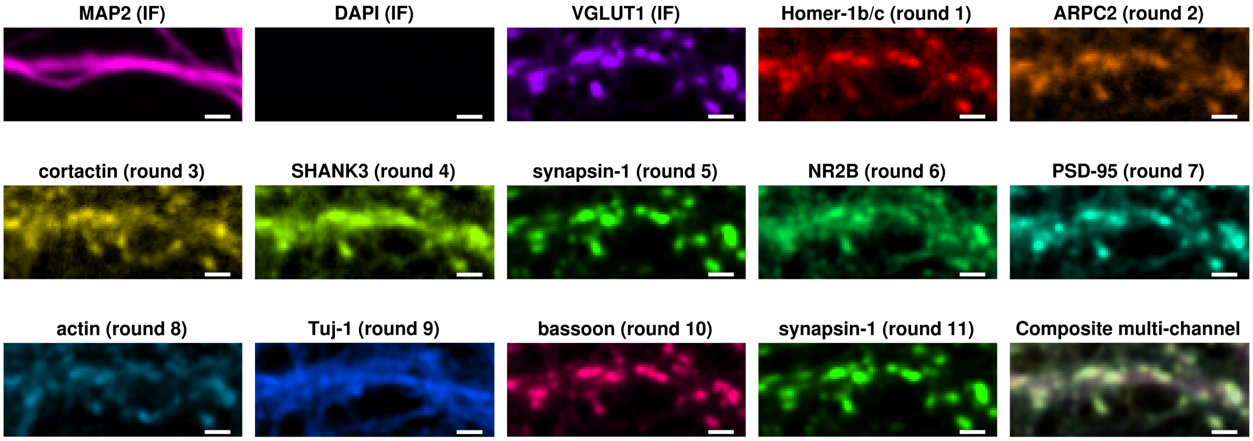

Dendrite 3

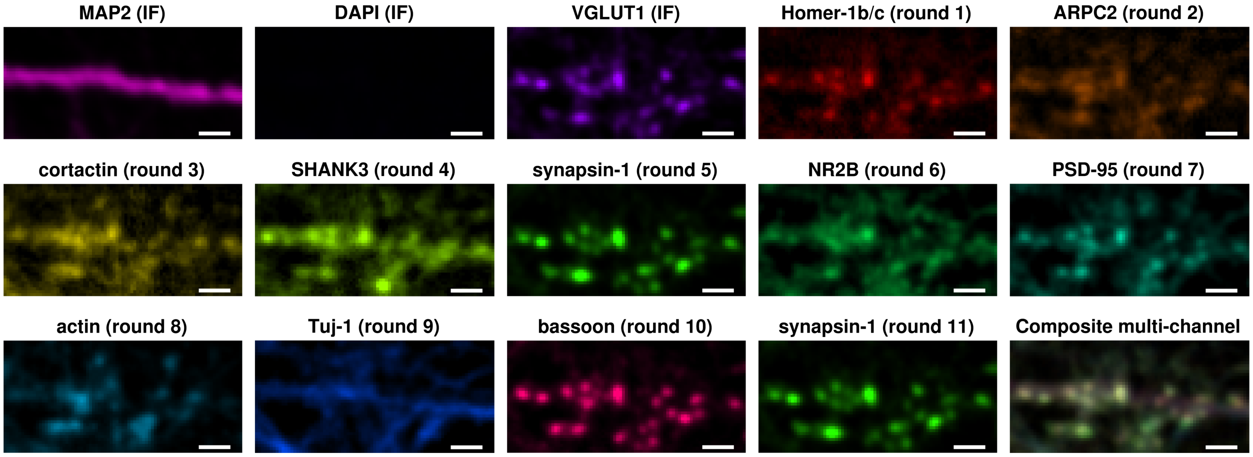

(Continued)

Dendrite 4

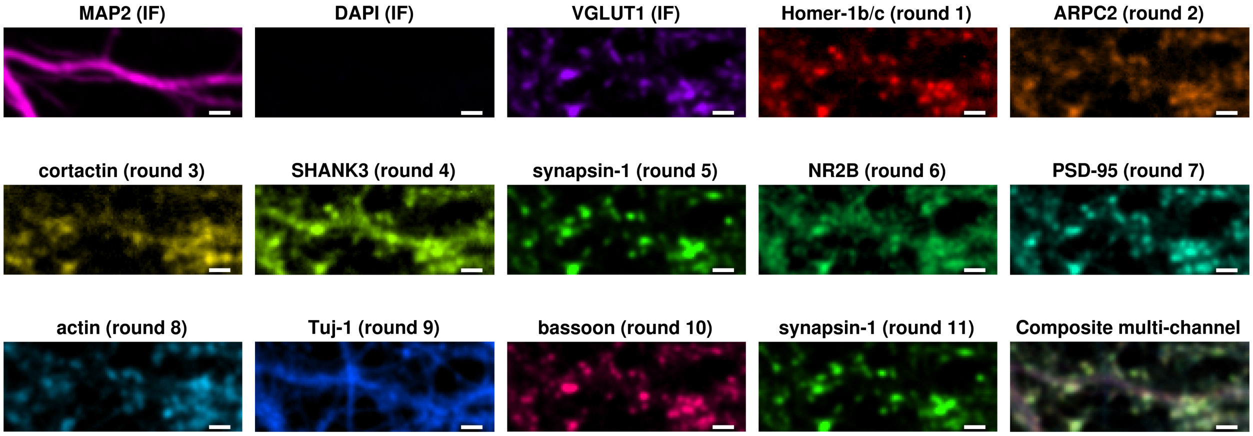

Dendrite 5

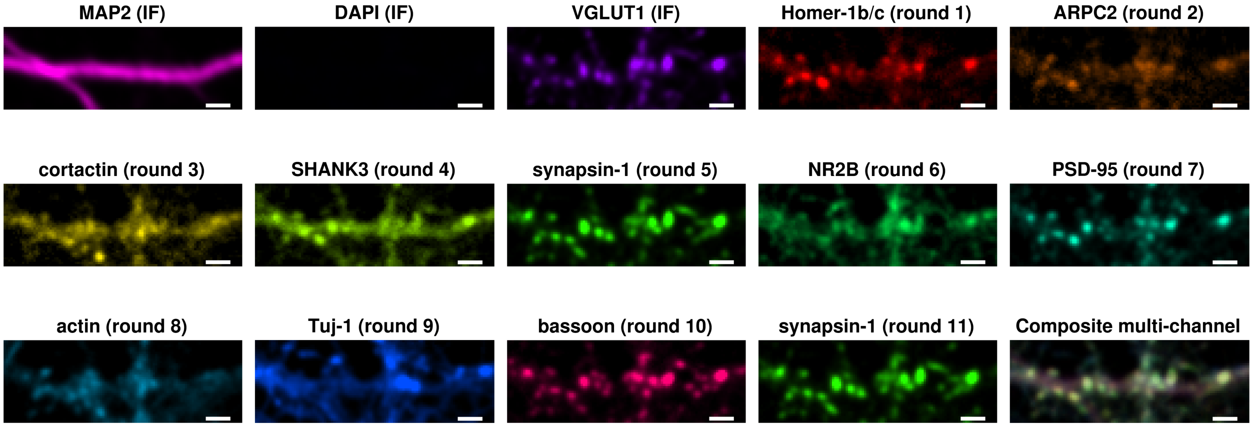

Dendrite 6

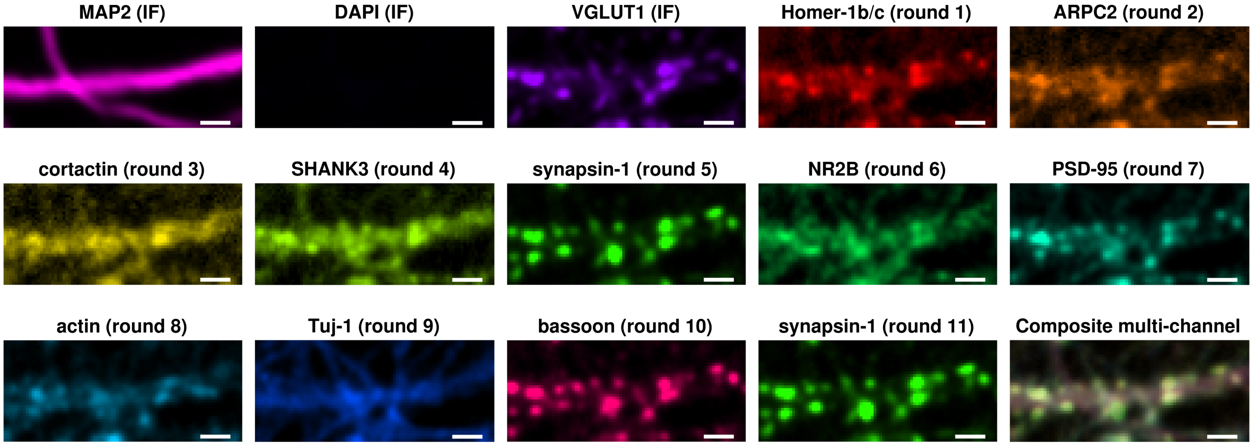

(Continued)

Dendrite 7

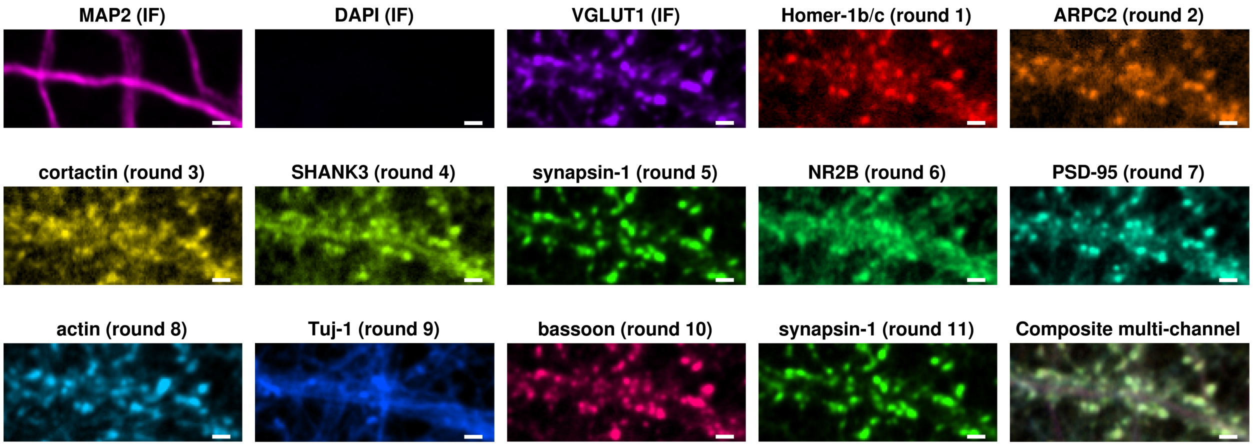

Dendrite 8

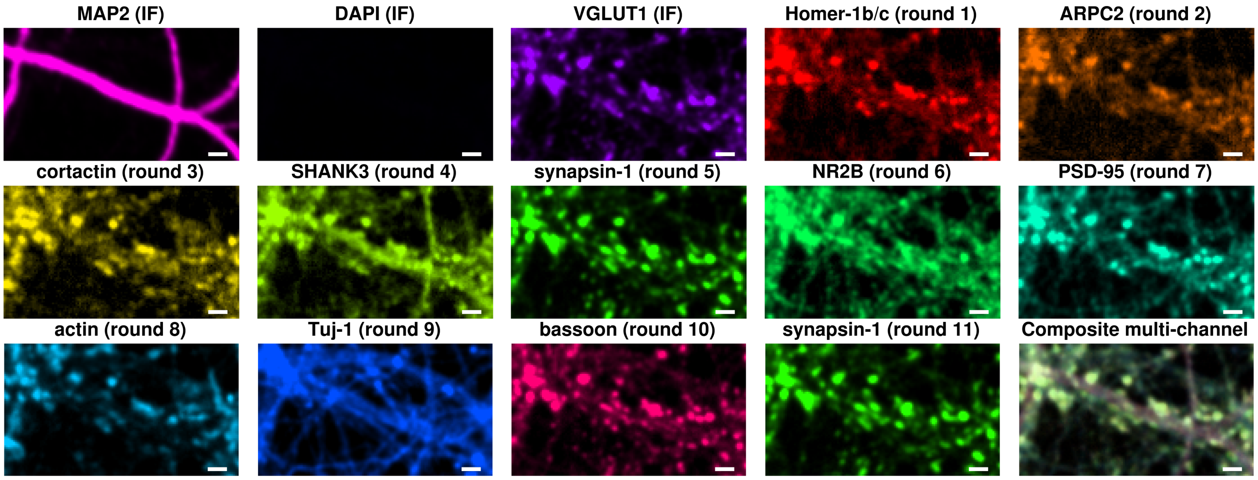

Supplementary Figure 14. Additional zoom-in confocal LNA-PRISM images of rat hippocampal neuronal dendrites. Synapsin-I was imaged twice, once in the middle and once at the end of the experiment. Scale bars: 2 μm.

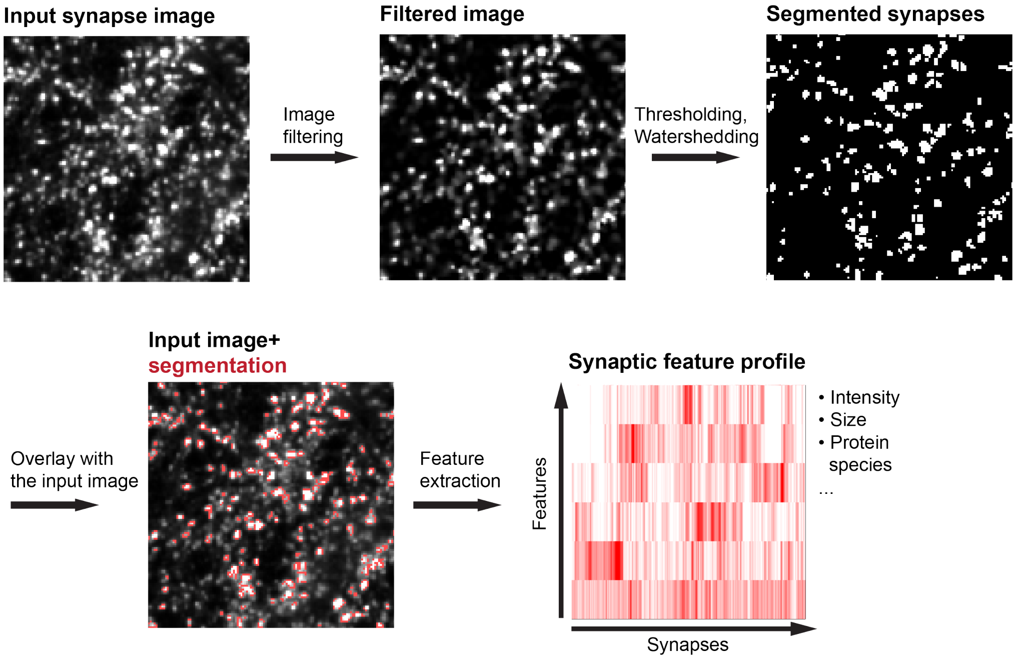

Supplementary Figure 15. Image-processing pipeline optimized for synapse segmentation to extract synaptic features including the size and intensity of each synaptic protein from LNA-PRISM images. See Methods for details.

synapsin-1

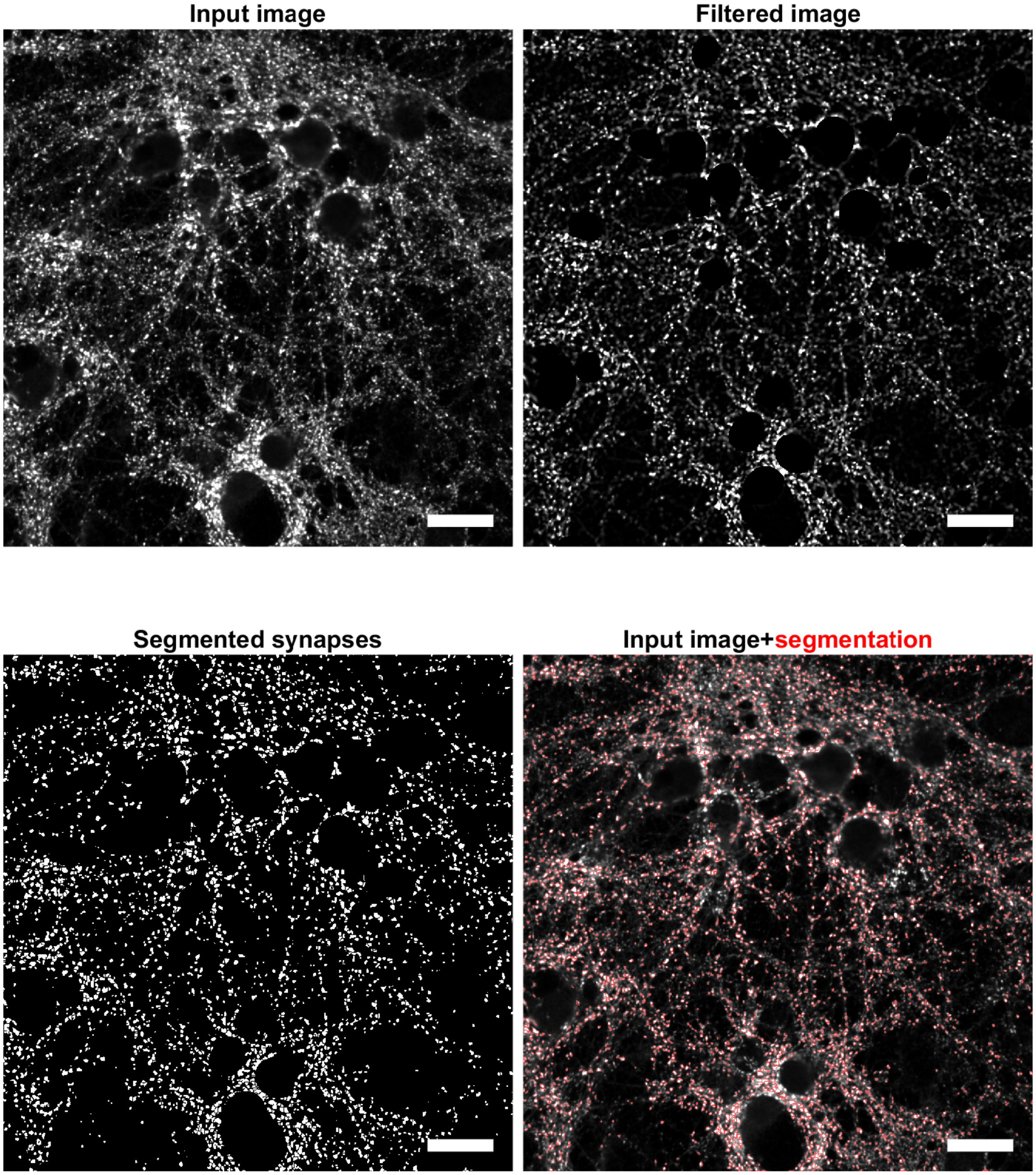

(Continued)

PSD-95

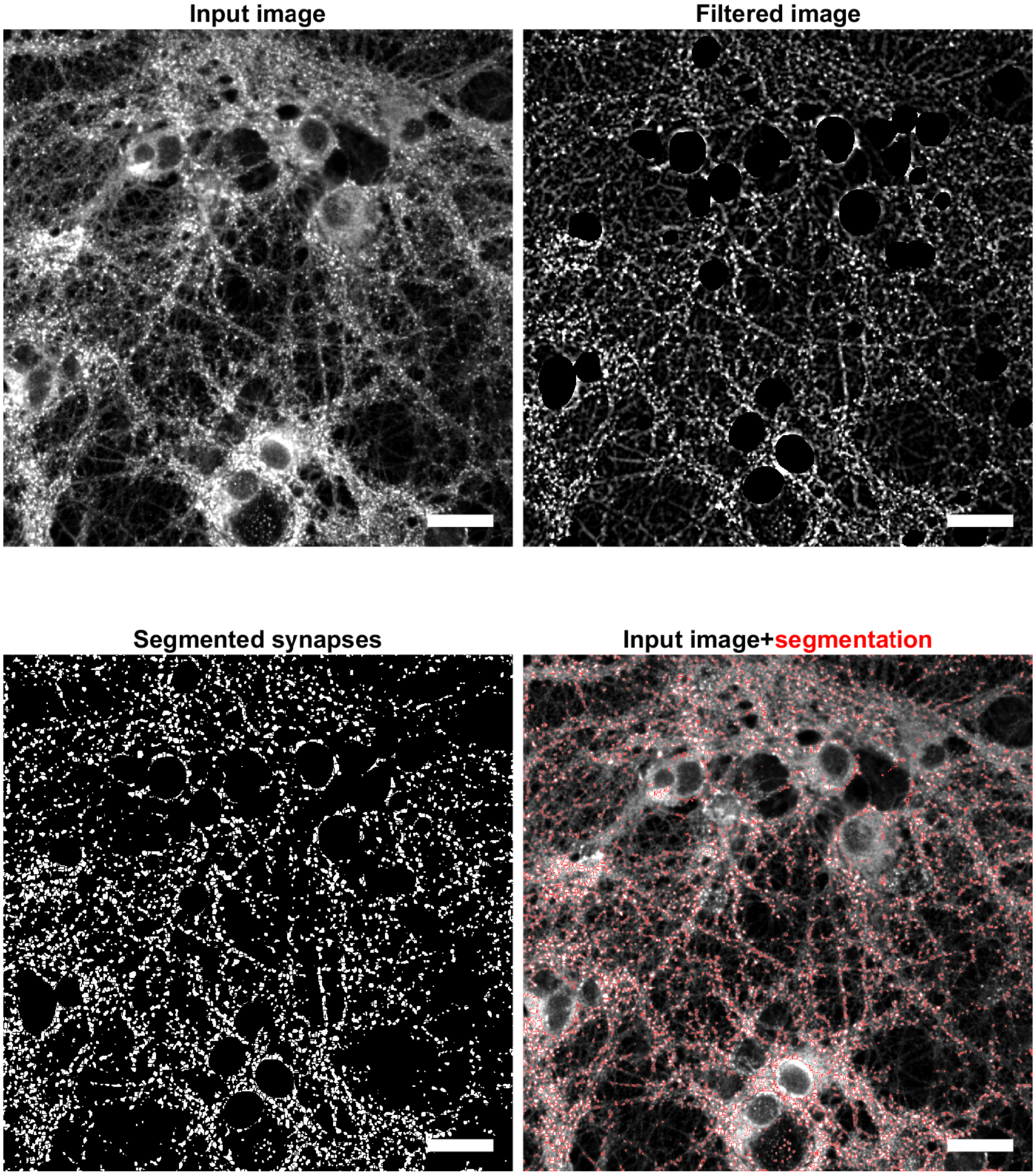

(Continued)

actin

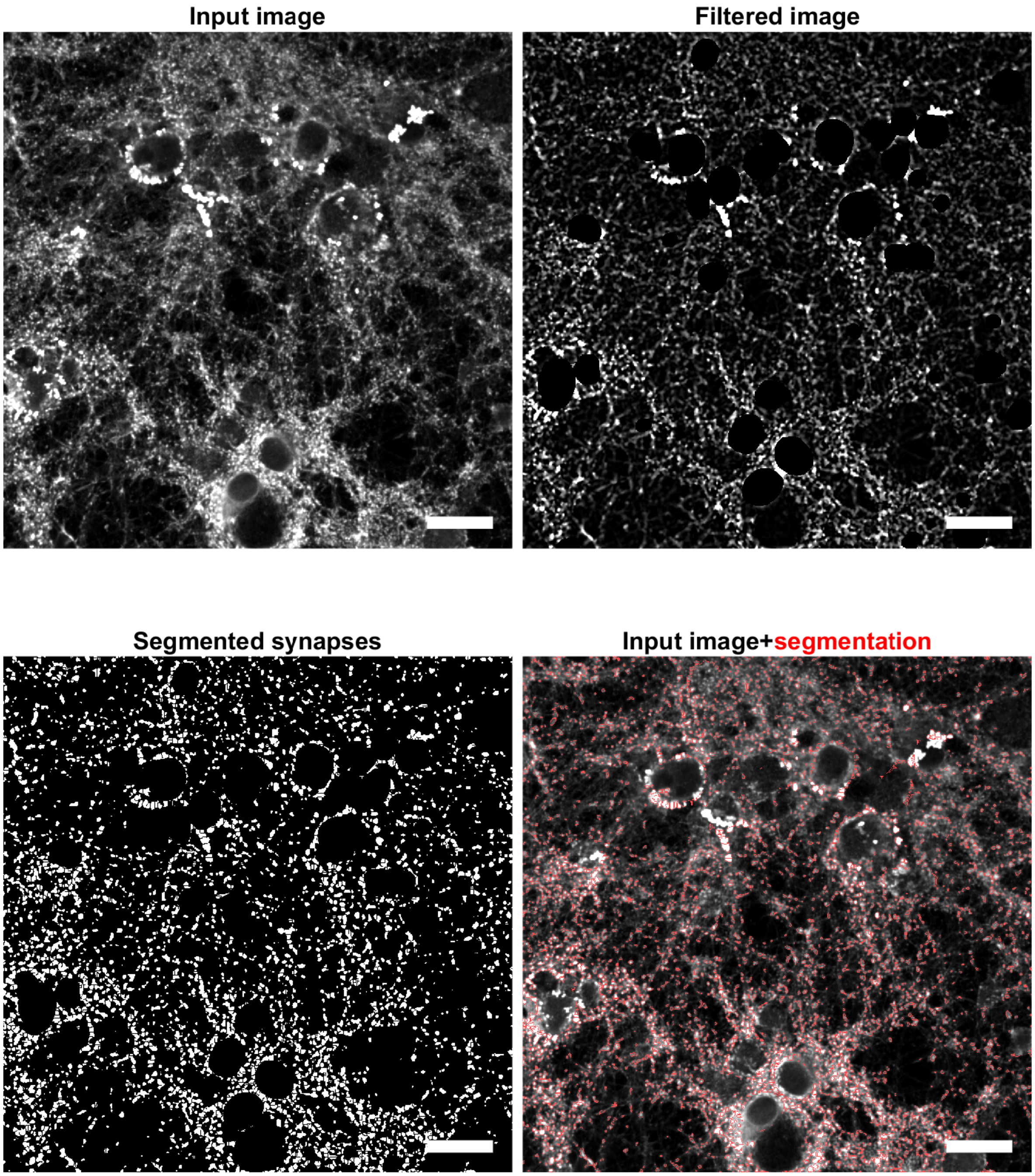

(Continued)

bassoon

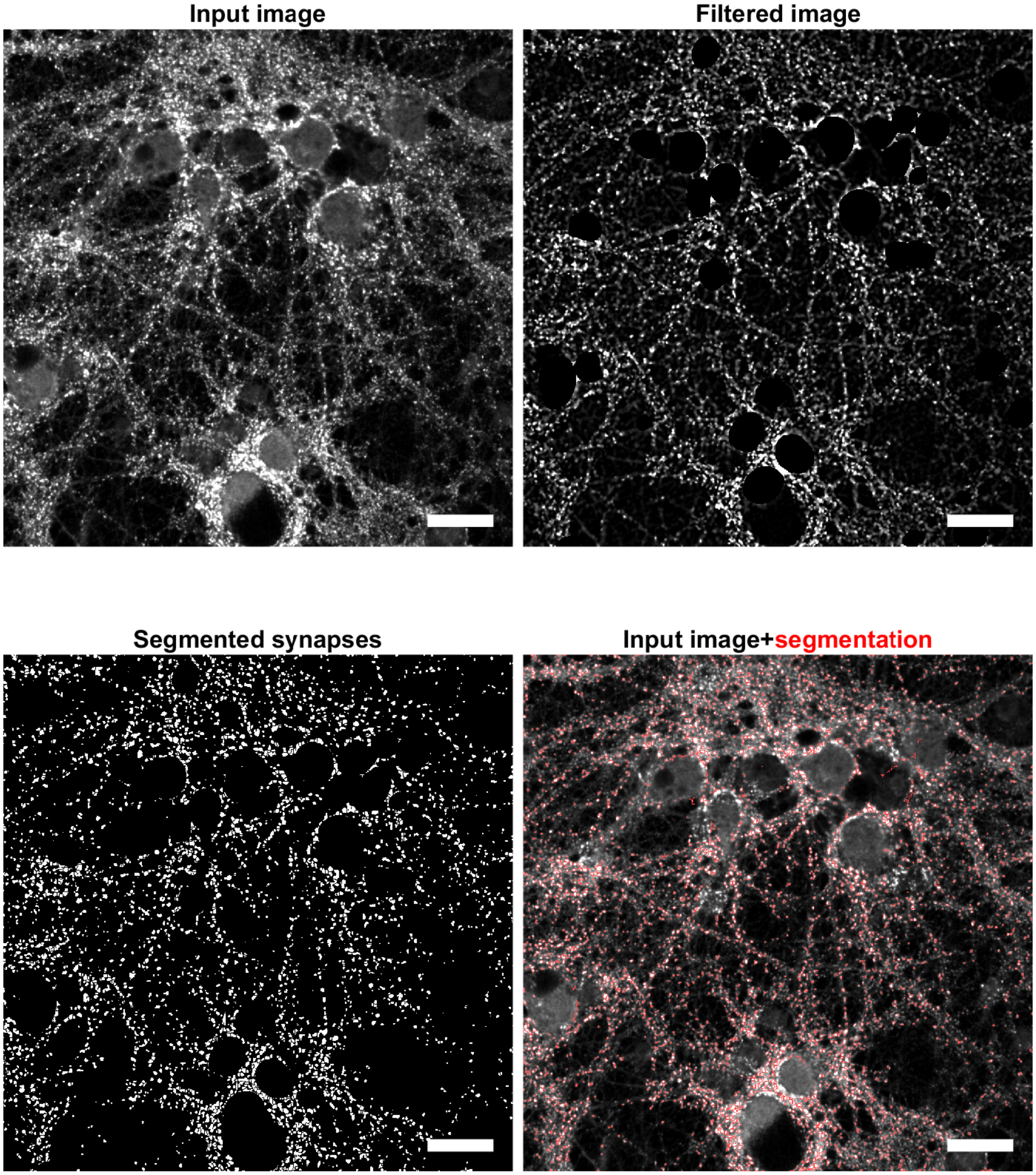

(Continued)

VGLUT1

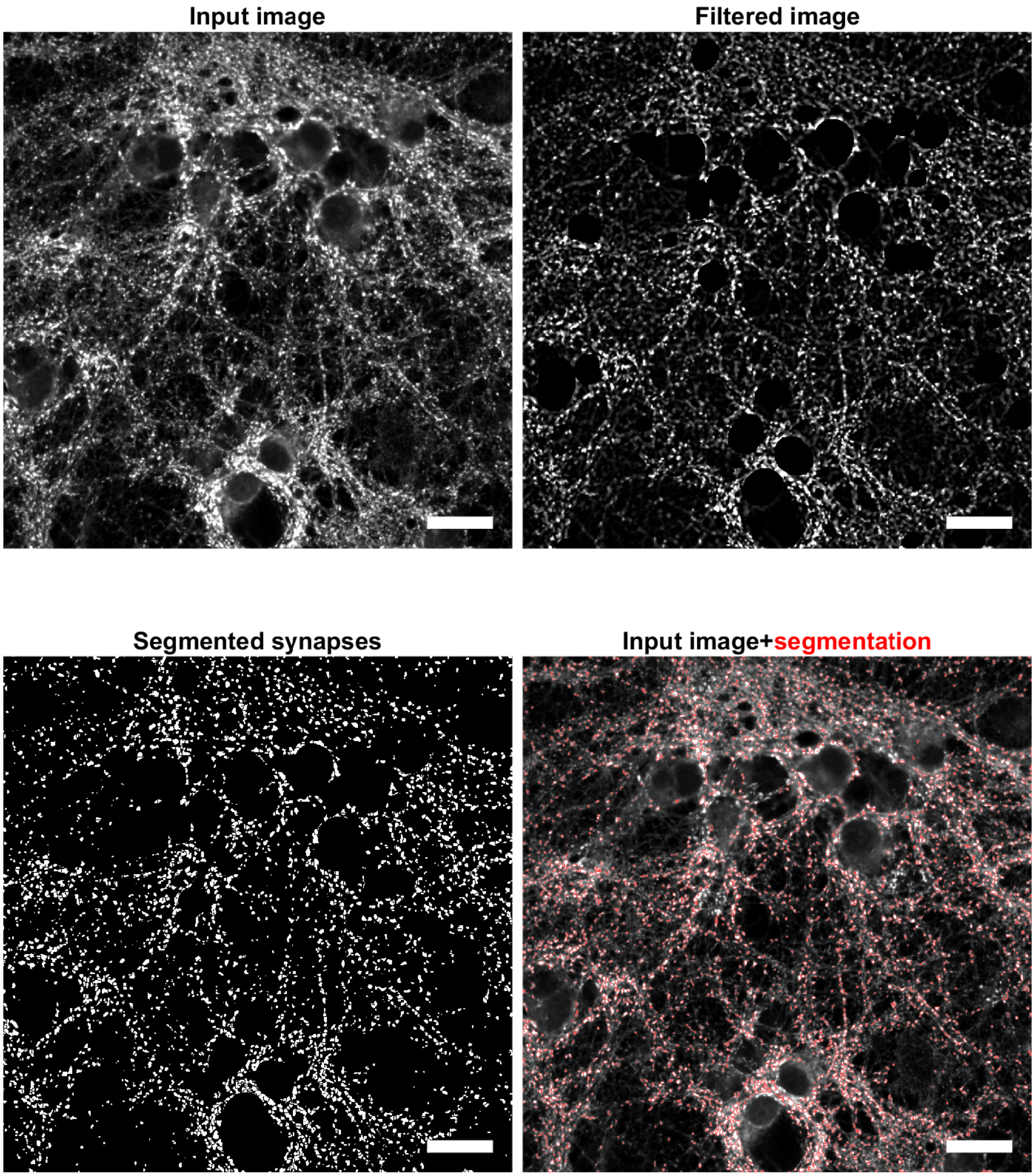

(Continued)

Homer-1b/c

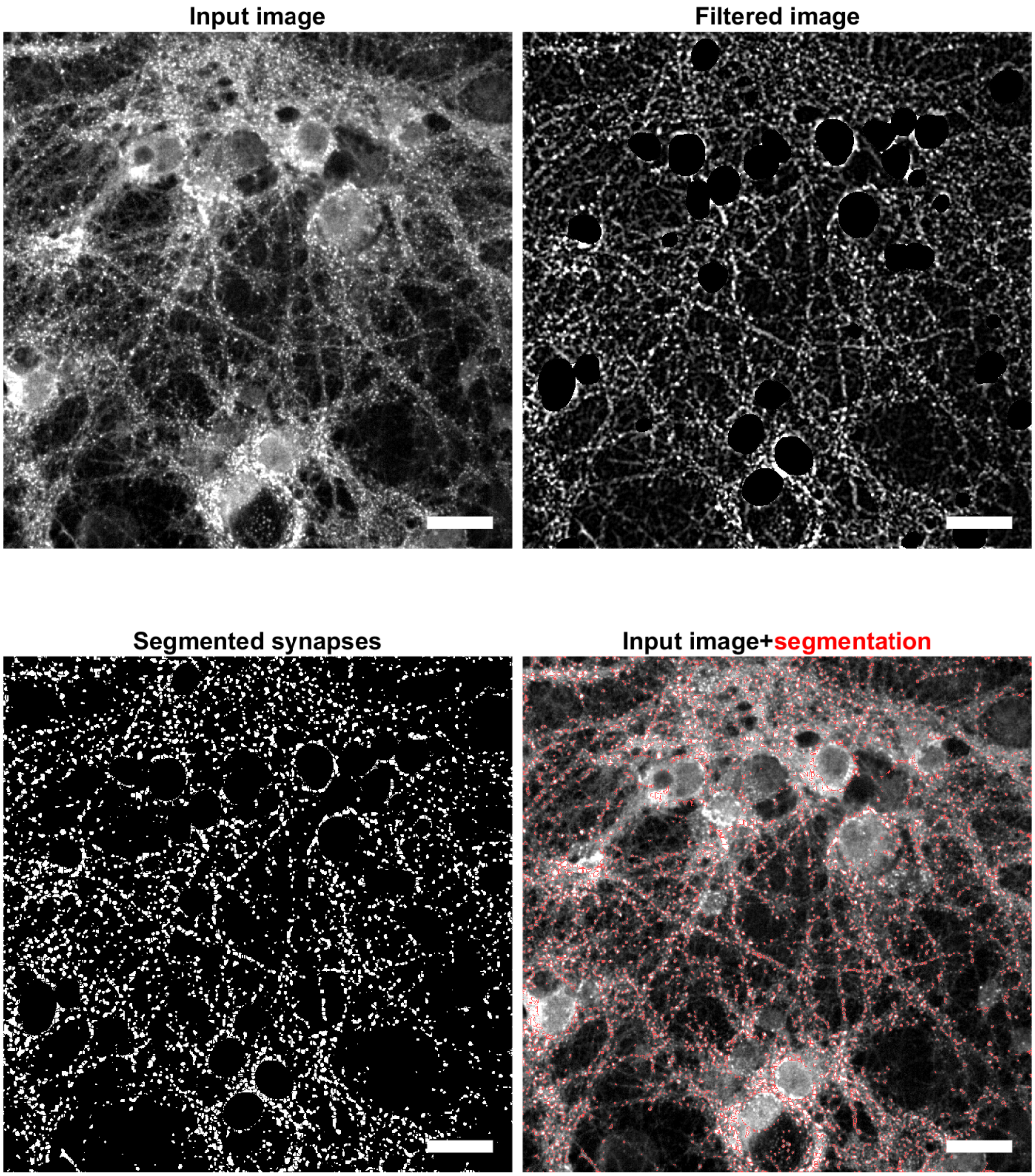

(Continued)

ARPC2

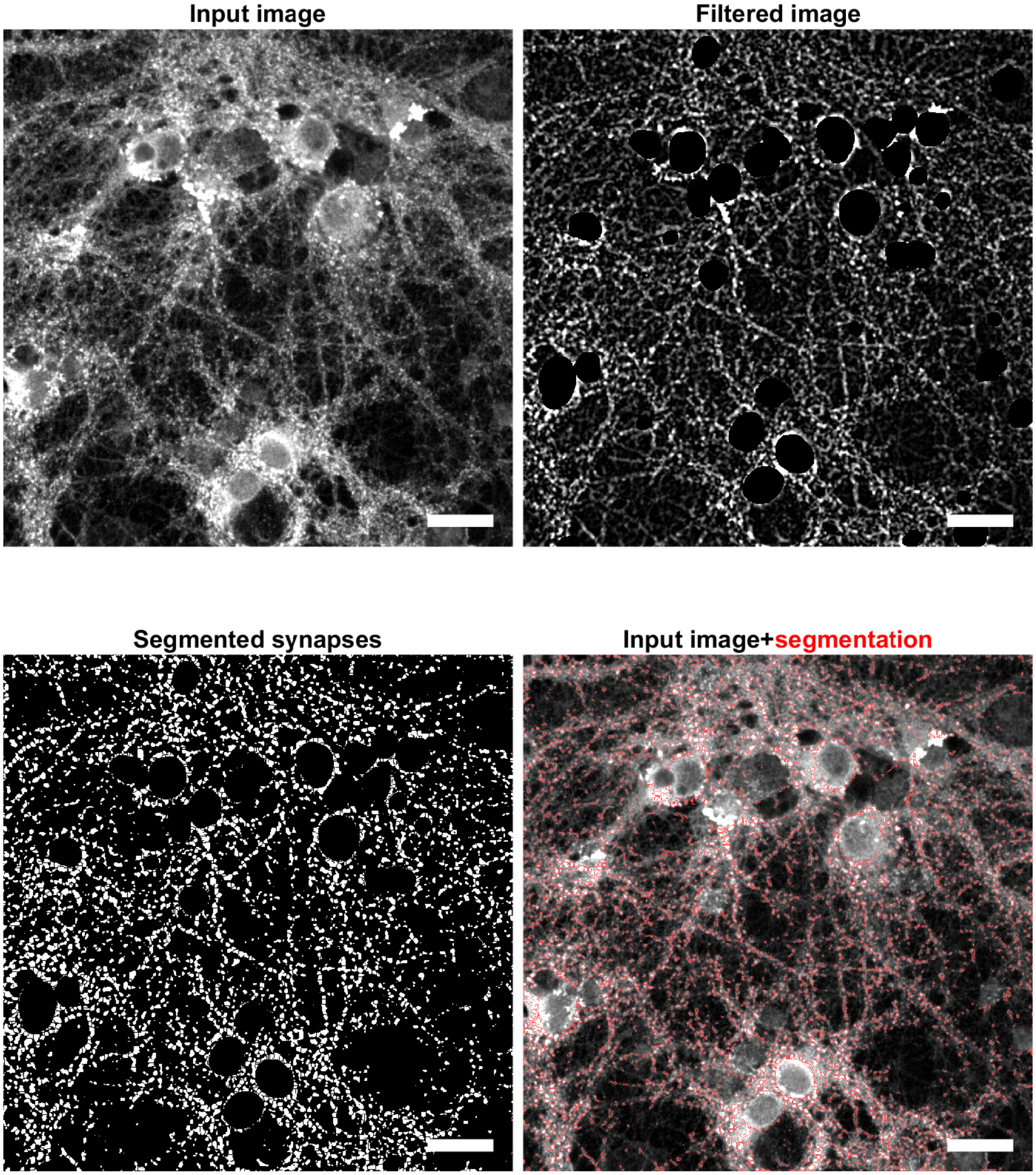

(Continued)

SHANK3

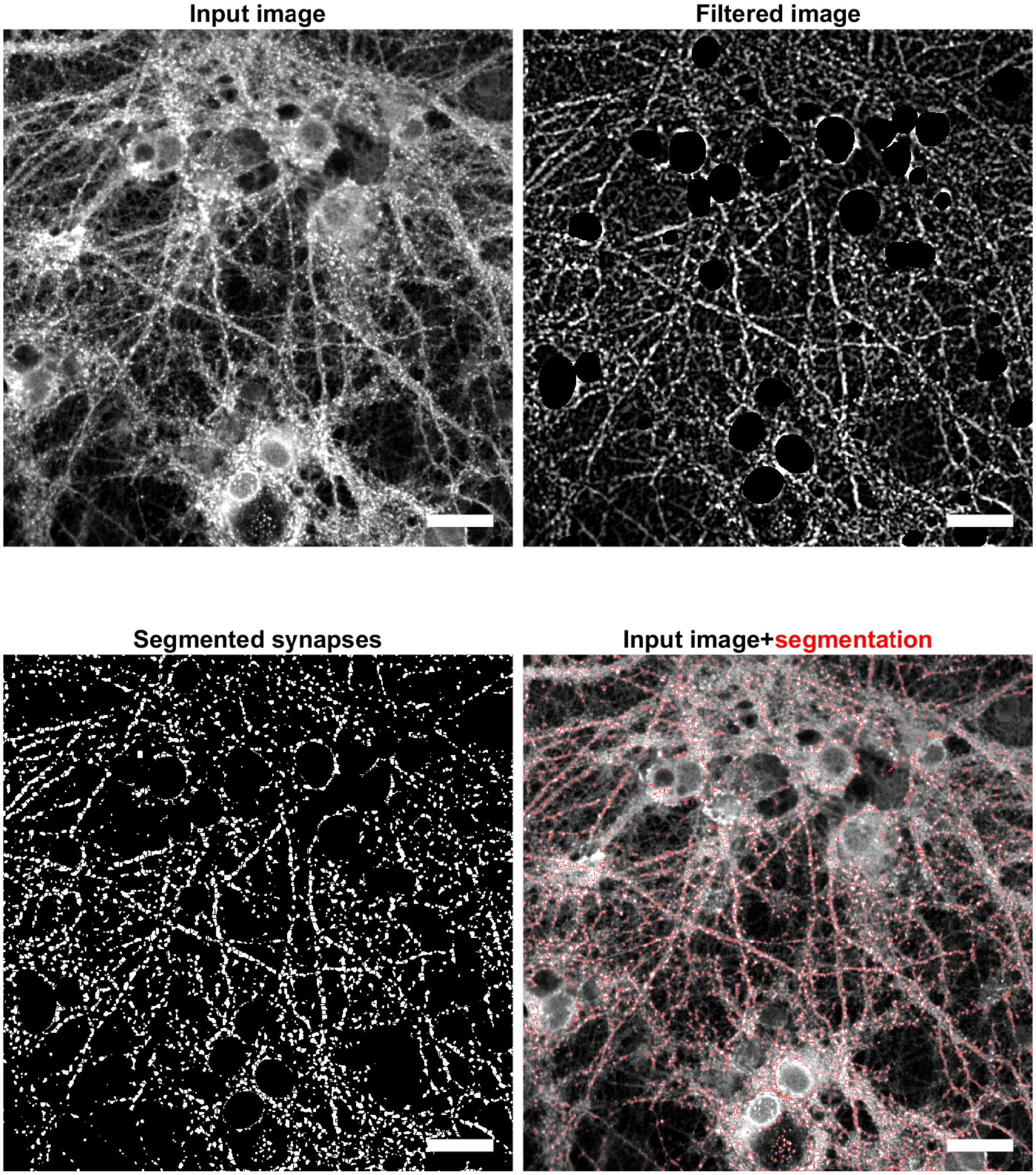

(Continued)

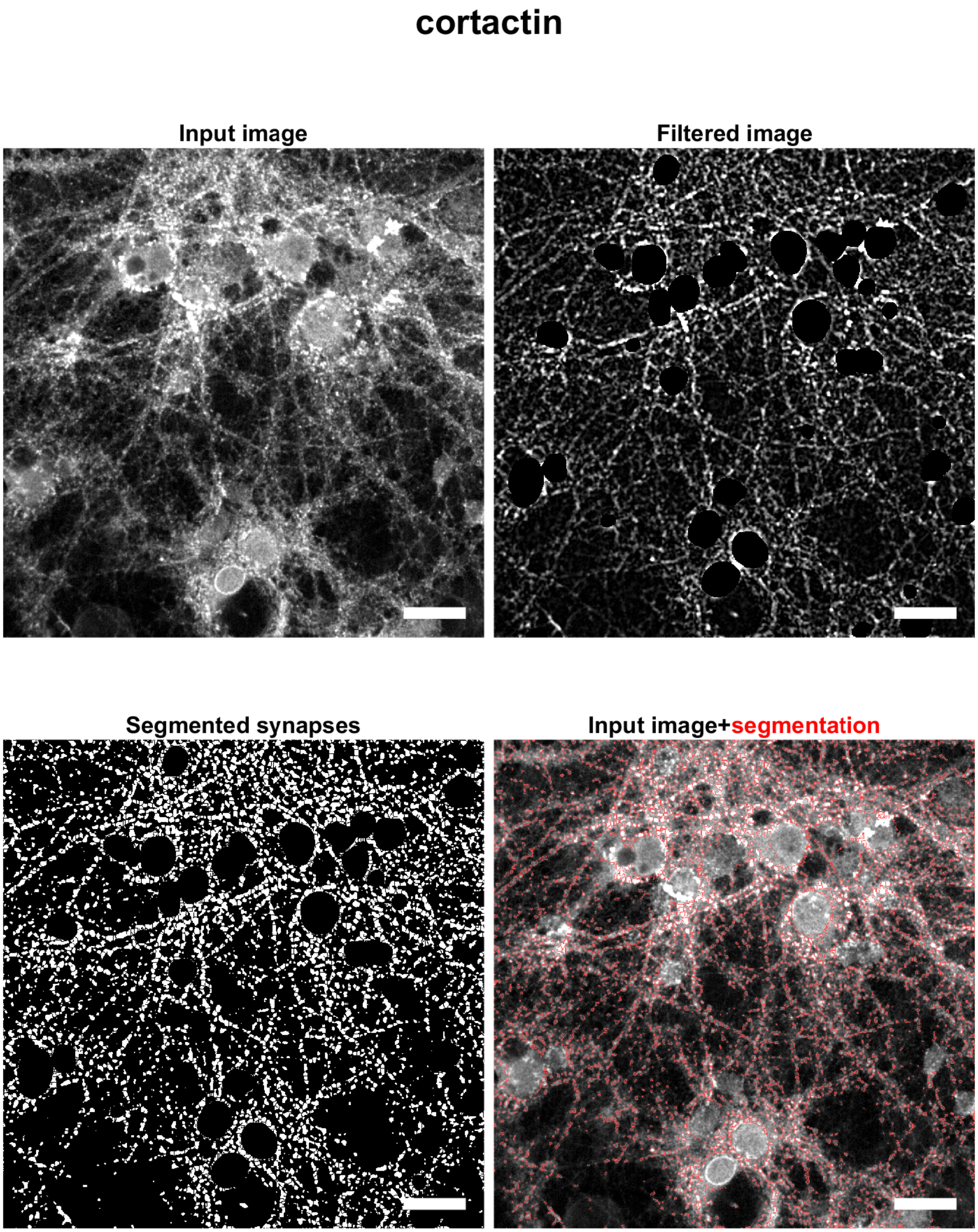

Supplementary Figure 16. Examples showing the processed LNA-PRISM images using the image-processing pipeline described in **Supplementary Figure 15** and **Materials and Methods** for quantifying synaptic features. Scale bars: 20 μm.

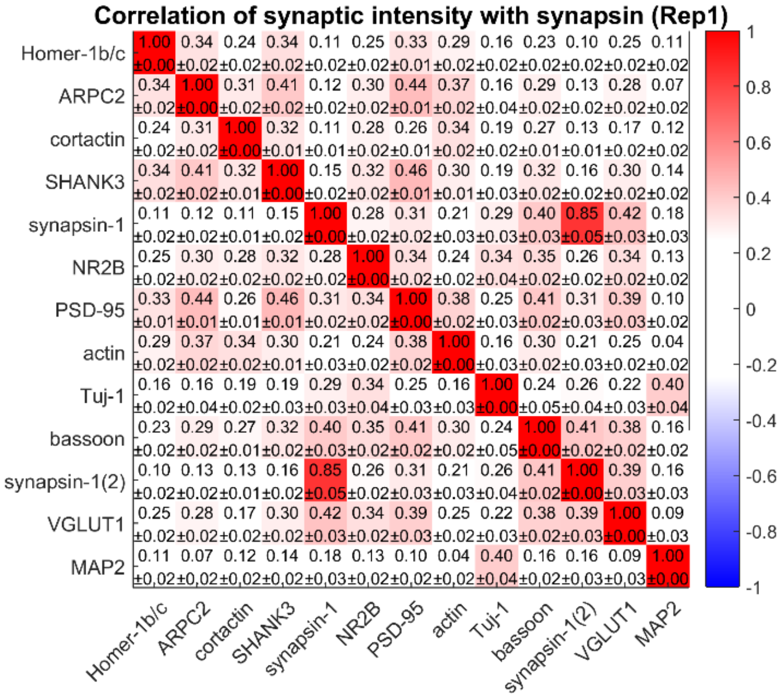
Correlation of synaptic intensity with synapsin (Rep1)
Homer-1b/c
ARPC2
cortactin
SHANK3
synapsin-1
NR2B
PSD-95
actin
Tuj-1
bassoon
synapsin-1(2)
VGLUT1
MAP2
1
0.8
0.6
0.4
0.2
0
-0.2
-0.4
-0.6
-0.8
-1

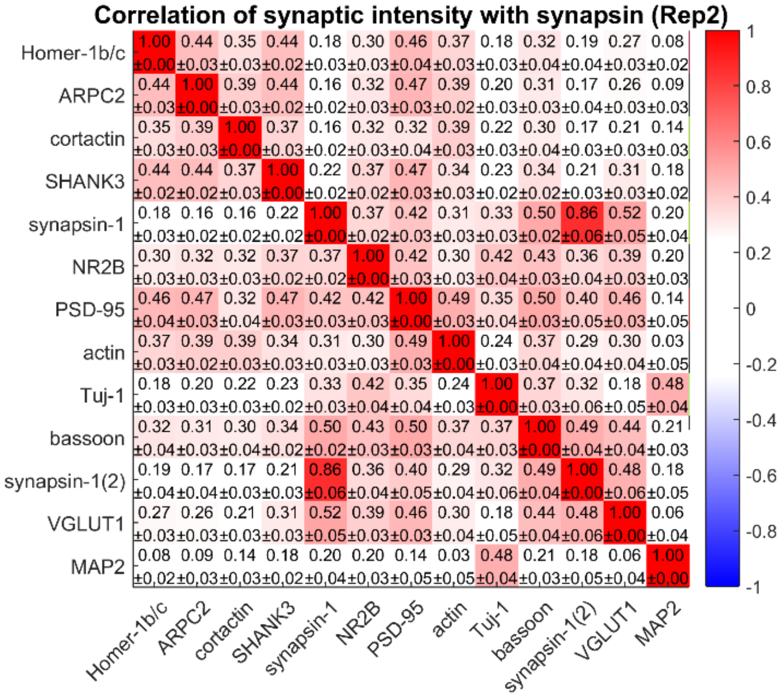
Correlation of synaptic intensity with synapsin (Rep2)
Homer-1b/c
ARPC2
cortactin
SHANK3
synapsin-1
NR2B
PSD-95
actin
Tuj-1
bassoon
synapsin-1(2)
VGLUT1
MAP2
1
0.8
0.6
0.4
0.2
0
-0.2
-0.4
-0.6
-0.8
-1

(Continued)

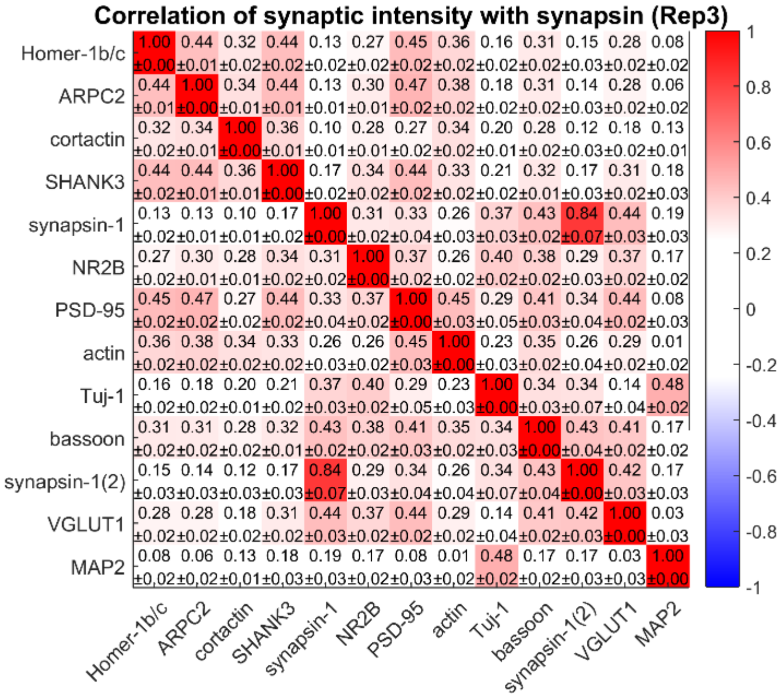

Supplementary Figure 17. PCC between intensity levels of synaptic proteins within synapses. Correlation matrices for 3 replicates are shown

Dendrite 1

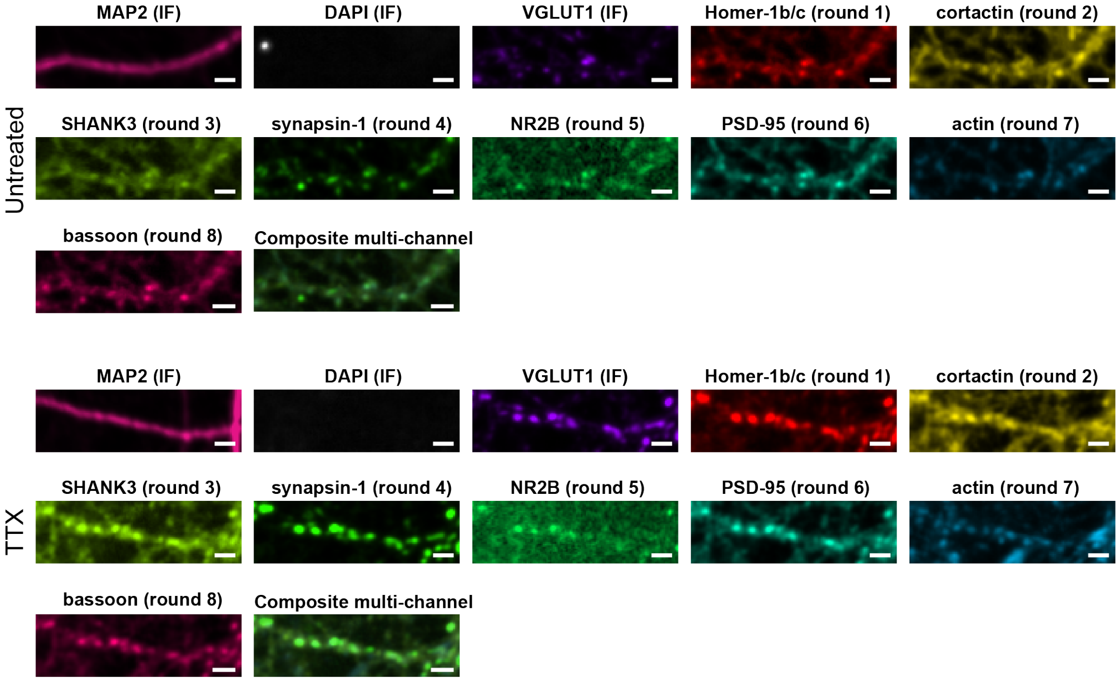

Dendrite 2

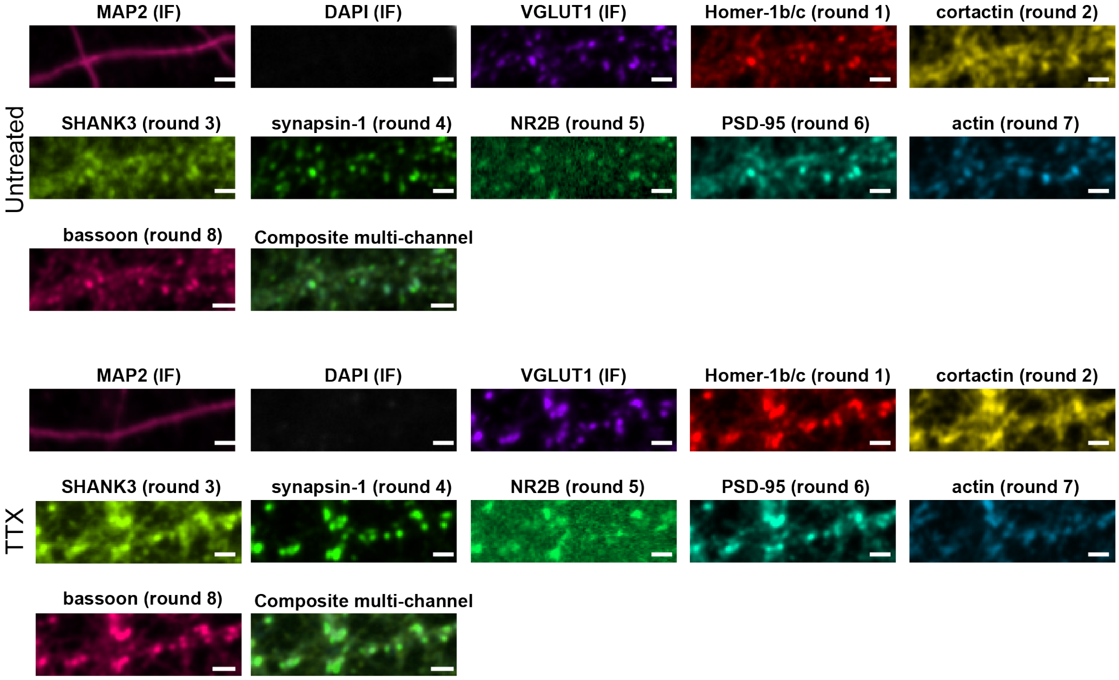

(Continued)

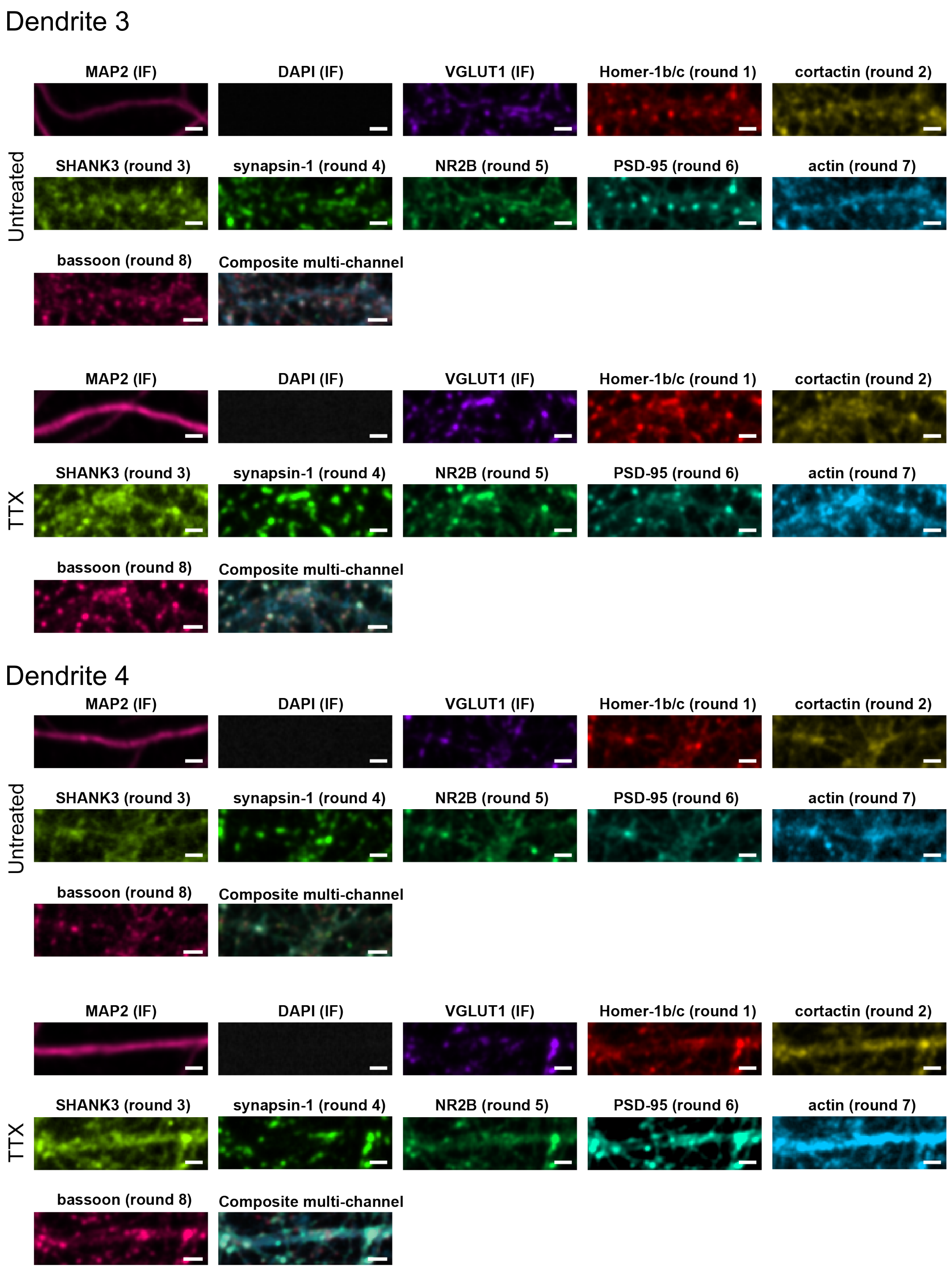

Supplementary Figure 18. Additional zoom-in confocal LNA-PRISM images of rat hippocampal neuronal dendrites from synaptic remodeling experiments. Scale bars: 2 μm.

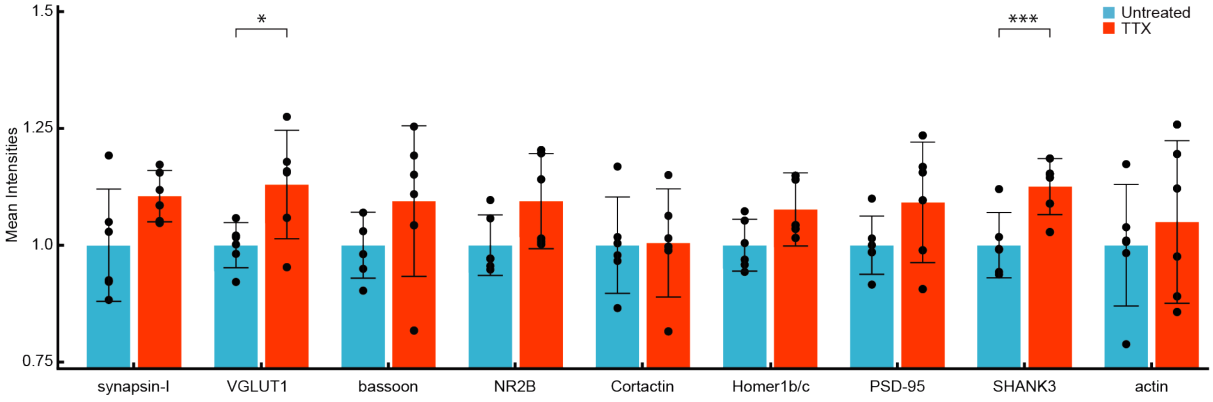

Supplementary Figure 19. Analysis of changes in mean intensities from multiplexed confocal imaging data acquired using LNA-PRISM. Bar heights represents average mean relative to average of untreated group. Points represent individual replicates. Error bars are 95% confidence intervals. P-values from Student's t-test.$p < 0.05$ (*) $p < 0.001$ (***)

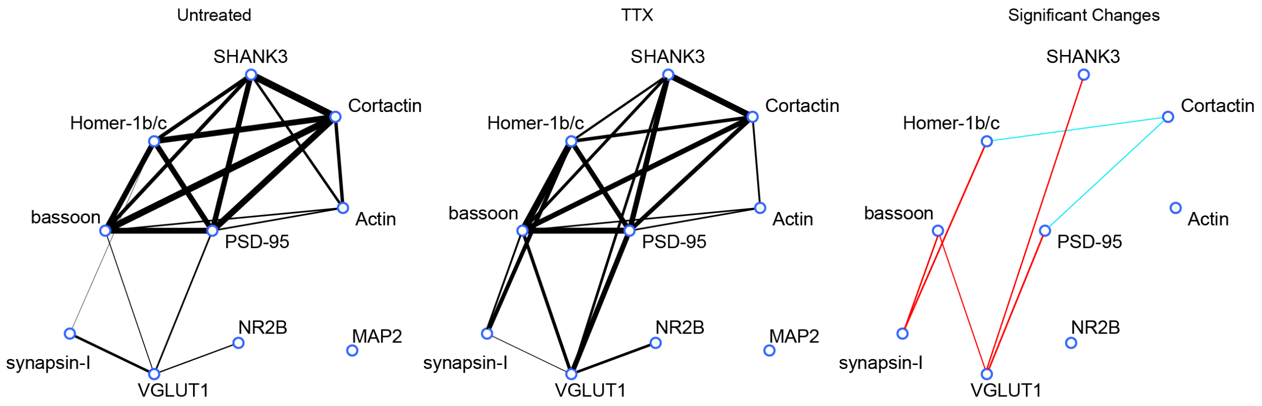

Supplementary Figure 20. Network representation of correlations between intensity levels of synaptic proteins within synapses (n=247,290 synapses from six replicates). The thickness of each edge represents the relative correlation strength between the respective nodes. Untreated (left) TTX Treated (Middle) Average difference from mean untreated (Right). Significant ($p < 0.05$) increases (red) and decreases (blue). P-value calculated from Student’s t-test.

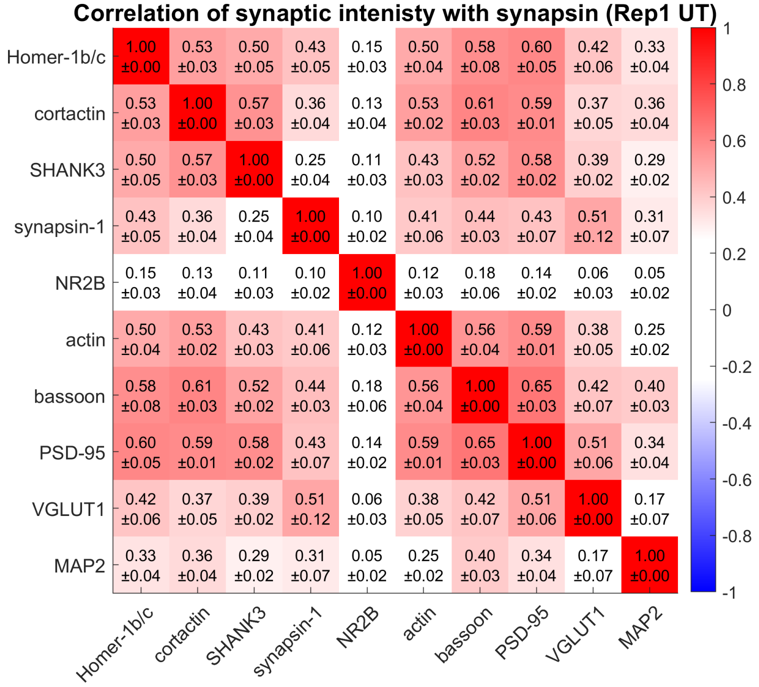
Correlation of synaptic intenisty with synapsin (Rep1 UT)
Homer-1b/c: 1.00 ±0.00, 0.53 ±0.03, 0.50 ±0.05, 0.43 ±0.05, 0.15 ±0.03, 0.50 ±0.04, 0.58 ±0.08, 0.60 ±0.05, 0.42 ±0.06, 0.33 ±0.04
cortactin: 0.53 ±0.03, 1.00 ±0.00, 0.57 ±0.03, 0.36 ±0.04, 0.13 ±0.04, 0.53 ±0.02, 0.61 ±0.03, 0.59 ±0.01, 0.37 ±0.05, 0.36 ±0.04
SHANK3: 0.50 ±0.05, 0.57 ±0.03, 1.00 ±0.00, 0.25 ±0.04, 0.11 ±0.03, 0.43 ±0.03, 0.52 ±0.02, 0.58 ±0.02, 0.39 ±0.02, 0.29 ±0.02
synapsin-1: 0.43 ±0.05, 0.36 ±0.04, 0.25 ±0.04, 1.00 ±0.00, 0.10 ±0.02, 0.41 ±0.06, 0.44 ±0.03, 0.43 ±0.07, 0.51 ±0.12, 0.31 ±0.07
NR2B: 0.15 ±0.03, 0.13 ±0.04, 0.11 ±0.03, 0.10 ±0.02, 1.00 ±0.00, 0.12 ±0.03, 0.18 ±0.06, 0.14 ±0.02, 0.06 ±0.03, 0.05 ±0.02
actin: 0.50 ±0.04, 0.53 ±0.02, 0.43 ±0.03, 0.41 ±0.06, 0.12 ±0.03, 1.00 ±0.00, 0.56 ±0.04, 0.59 ±0.01, 0.38 ±0.05, 0.25 ±0.02
bassoon: 0.58 ±0.08, 0.61 ±0.03, 0.52 ±0.02, 0.44 ±0.03, 0.18 ±0.06, 0.56 ±0.04, 1.00 ±0.00, 0.65 ±0.03, 0.42 ±0.07, 0.40 ±0.03
PSD-95: 0.60 ±0.05, 0.59 ±0.01, 0.58 ±0.02, 0.43 ±0.07, 0.14 ±0.02, 0.59 ±0.01, 0.65 ±0.03, 1.00 ±0.00, 0.51 ±0.06, 0.34 ±0.04
VGLUT1: 0.42 ±0.06, 0.37 ±0.05, 0.39 ±0.02, 0.51 ±0.12, 0.06 ±0.03, 0.38 ±0.05, 0.42 ±0.07, 0.51 ±0.06, 1.00 ±0.00, 0.17 ±0.07
MAP2: 0.33 ±0.04, 0.36 ±0.04, 0.29 ±0.02, 0.31 ±0.07, 0.05 ±0.02, 0.25 ±0.02, 0.40 ±0.03, 0.34 ±0.04, 0.17 ±0.07, 1.00 ±0.00
Homer-1b/c cortactin SHANK3 synapsin-1 NR2B actin bassoon PSD-95 VGLUT1 MAP2
1 0.8 0.6 0.4 0.2 0 -0.2 -0.4 -0.6 -0.8 -1

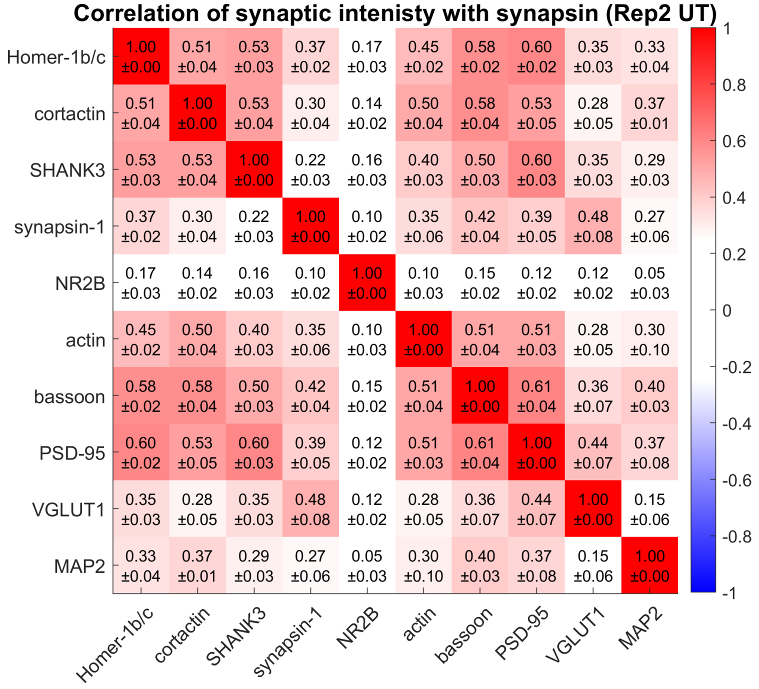
Correlation of synaptic intenisty with synapsin (Rep2 UT)
Homer-1b/c: 1.00 ±0.00, 0.51 ±0.04, 0.53 ±0.03, 0.37 ±0.02, 0.17 ±0.03, 0.45 ±0.02, 0.58 ±0.02, 0.60 ±0.02, 0.35 ±0.03, 0.33 ±0.04
cortactin: 0.51 ±0.04, 1.00 ±0.00, 0.53 ±0.04, 0.30 ±0.04, 0.14 ±0.02, 0.50 ±0.04, 0.58 ±0.04, 0.53 ±0.05, 0.28 ±0.05, 0.37 ±0.01
SHANK3: 0.53 ±0.03, 0.53 ±0.04, 1.00 ±0.00, 0.22 ±0.03, 0.16 ±0.03, 0.40 ±0.03, 0.50 ±0.03, 0.60 ±0.03, 0.35 ±0.03, 0.29 ±0.03
synapsin-1: 0.37 ±0.02, 0.30 ±0.04, 0.22 ±0.03, 1.00 ±0.00, 0.10 ±0.02, 0.35 ±0.06, 0.42 ±0.04, 0.39 ±0.05, 0.48 ±0.08, 0.27 ±0.06
NR2B: 0.17 ±0.03, 0.14 ±0.02, 0.16 ±0.03, 0.10 ±0.02, 1.00 ±0.00, 0.10 ±0.03, 0.15 ±0.02, 0.12 ±0.02, 0.12 ±0.02, 0.05 ±0.03
actin: 0.45 ±0.02, 0.50 ±0.04, 0.40 ±0.03, 0.35 ±0.06, 0.10 ±0.03, 1.00 ±0.00, 0.51 ±0.04, 0.51 ±0.03, 0.28 ±0.05, 0.30 ±0.10
bassoon: 0.58 ±0.02, 0.58 ±0.04, 0.50 ±0.03, 0.42 ±0.04, 0.15 ±0.02, 0.51 ±0.04, 1.00 ±0.00, 0.61 ±0.04, 0.36 ±0.07, 0.40 ±0.03
PSD-95: 0.60 ±0.02, 0.53 ±0.05, 0.60 ±0.03, 0.39 ±0.05, 0.12 ±0.02, 0.51 ±0.03, 0.61 ±0.04, 1.00 ±0.00, 0.44 ±0.07, 0.37 ±0.08
VGLUT1: 0.35 ±0.03, 0.28 ±0.05, 0.35 ±0.03, 0.48 ±0.08, 0.12 ±0.02, 0.28 ±0.05, 0.36 ±0.07, 0.44 ±0.07, 1.00 ±0.00, 0.15 ±0.06
MAP2: 0.33 ±0.04, 0.37 ±0.01, 0.29 ±0.03, 0.27 ±0.06, 0.05 ±0.03, 0.30 ±0.10, 0.40 ±0.03, 0.37 ±0.08, 0.15 ±0.06, 1.00 ±0.00
Homer-1b/c cortactin SHANK3 synapsin-1 NR2B actin bassoon PSD-95 VGLUT1 MAP2
1 0.8 0.6 0.4 0.2 0 -0.2 -0.4 -0.6 -0.8 -1

(Continued)

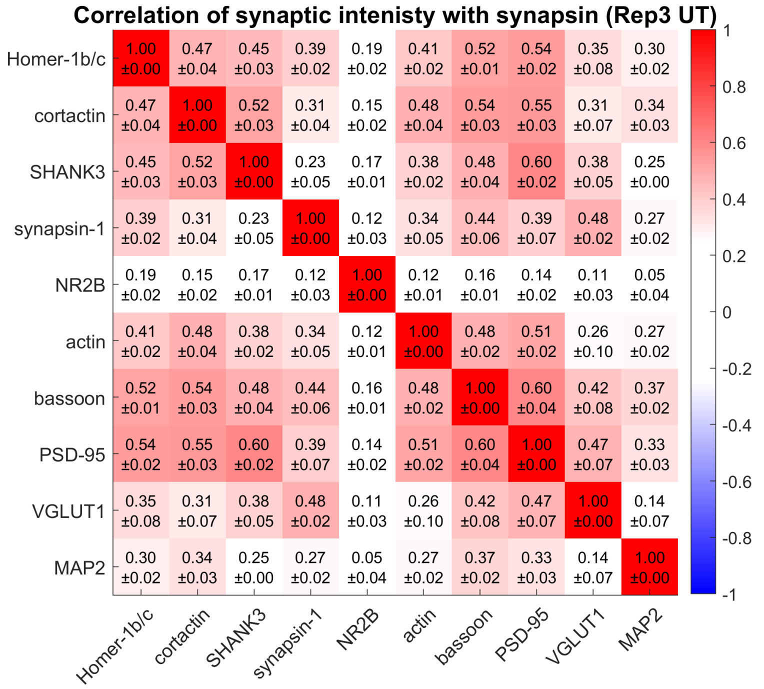
Correlation of synaptic intenisty with synapsin (Rep3 UT)
Homer-1b/c: 1.00 ±0.00, 0.47 ±0.04, 0.45 ±0.03, 0.39 ±0.02, 0.19 ±0.02, 0.41 ±0.02, 0.52 ±0.01, 0.54 ±0.02, 0.35 ±0.08, 0.30 ±0.02
cortactin: 0.47 ±0.04, 1.00 ±0.00, 0.52 ±0.03, 0.31 ±0.04, 0.15 ±0.02, 0.48 ±0.04, 0.54 ±0.03, 0.55 ±0.03, 0.31 ±0.07, 0.34 ±0.03
SHANK3: 0.45 ±0.03, 0.52 ±0.03, 1.00 ±0.00, 0.23 ±0.05, 0.17 ±0.01, 0.38 ±0.02, 0.48 ±0.04, 0.60 ±0.02, 0.38 ±0.05, 0.25 ±0.00
synapsin-1: 0.39 ±0.02, 0.31 ±0.04, 0.23 ±0.05, 1.00 ±0.00, 0.12 ±0.03, 0.34 ±0.05, 0.44 ±0.06, 0.39 ±0.07, 0.48 ±0.02, 0.27 ±0.02
NR2B: 0.19 ±0.02, 0.15 ±0.02, 0.17 ±0.01, 0.12 ±0.03, 1.00 ±0.00, 0.12 ±0.01, 0.16 ±0.01, 0.14 ±0.02, 0.11 ±0.03, 0.05 ±0.04
actin: 0.41 ±0.02, 0.48 ±0.04, 0.38 ±0.02, 0.34 ±0.05, 0.12 ±0.01, 1.00 ±0.00, 0.48 ±0.02, 0.51 ±0.02, 0.26 ±0.10, 0.27 ±0.02
bassoon: 0.52 ±0.01, 0.54 ±0.03, 0.48 ±0.04, 0.44 ±0.06, 0.16 ±0.01, 0.48 ±0.02, 1.00 ±0.00, 0.60 ±0.04, 0.42 ±0.08, 0.37 ±0.02
PSD-95: 0.54 ±0.02, 0.55 ±0.03, 0.60 ±0.02, 0.39 ±0.07, 0.14 ±0.02, 0.51 ±0.02, 0.60 ±0.04, 1.00 ±0.00, 0.47 ±0.07, 0.33 ±0.03
VGLUT1: 0.35 ±0.08, 0.31 ±0.07, 0.38 ±0.05, 0.48 ±0.02, 0.11 ±0.03, 0.26 ±0.10, 0.42 ±0.08, 0.47 ±0.07, 1.00 ±0.00, 0.14 ±0.07
MAP2: 0.30 ±0.02, 0.34 ±0.03, 0.25 ±0.00, 0.27 ±0.02, 0.05 ±0.04, 0.27 ±0.02, 0.37 ±0.02, 0.33 ±0.03, 0.14 ±0.07, 1.00 ±0.00
Homer-1b/c cortactin SHANK3 synapsin-1 NR2B actin bassoon PSD-95 VGLUT1 MAP2
1 0.8 0.6 0.4 0.2 0 -0.2 -0.4 -0.6 -0.8 -1

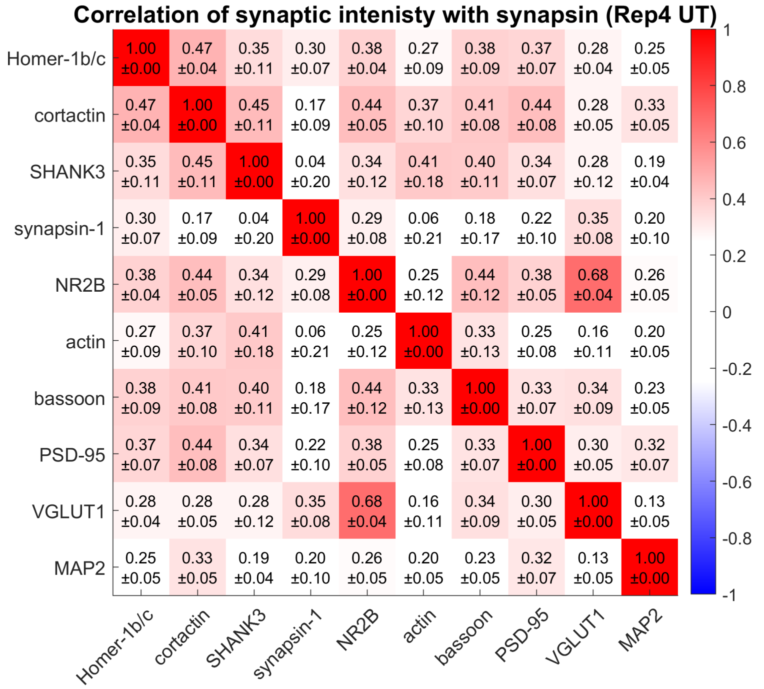
Correlation of synaptic intenisty with synapsin (Rep4 UT)
Homer-1b/c: 1.00 ±0.00, 0.47 ±0.04, 0.35 ±0.11, 0.30 ±0.07, 0.38 ±0.04, 0.27 ±0.09, 0.38 ±0.09, 0.37 ±0.07, 0.28 ±0.04, 0.25 ±0.05
cortactin: 0.47 ±0.04, 1.00 ±0.00, 0.45 ±0.11, 0.17 ±0.09, 0.44 ±0.05, 0.37 ±0.10, 0.41 ±0.08, 0.44 ±0.08, 0.28 ±0.05, 0.33 ±0.05
SHANK3: 0.35 ±0.11, 0.45 ±0.11, 1.00 ±0.00, 0.04 ±0.20, 0.34 ±0.12, 0.41 ±0.18, 0.40 ±0.11, 0.34 ±0.07, 0.28 ±0.12, 0.19 ±0.04
synapsin-1: 0.30 ±0.07, 0.17 ±0.09, 0.04 ±0.20, 1.00 ±0.00, 0.29 ±0.08, 0.06 ±0.21, 0.18 ±0.17, 0.22 ±0.10, 0.35 ±0.08, 0.20 ±0.10
NR2B: 0.38 ±0.04, 0.44 ±0.05, 0.34 ±0.12, 0.29 ±0.08, 1.00 ±0.00, 0.25 ±0.12, 0.44 ±0.12, 0.38 ±0.05, 0.68 ±0.04, 0.26 ±0.05
actin: 0.27 ±0.09, 0.37 ±0.10, 0.41 ±0.18, 0.06 ±0.21, 0.25 ±0.12, 1.00 ±0.00, 0.33 ±0.13, 0.25 ±0.08, 0.16 ±0.11, 0.20 ±0.05
bassoon: 0.38 ±0.09, 0.41 ±0.08, 0.40 ±0.11, 0.18 ±0.17, 0.44 ±0.12, 0.33 ±0.13, 1.00 ±0.00, 0.33 ±0.07, 0.34 ±0.09, 0.23 ±0.05
PSD-95: 0.37 ±0.07, 0.44 ±0.08, 0.34 ±0.07, 0.22 ±0.10, 0.38 ±0.05, 0.25 ±0.08, 0.33 ±0.07, 1.00 ±0.00, 0.30 ±0.05, 0.32 ±0.07
VGLUT1: 0.28 ±0.04, 0.28 ±0.05, 0.28 ±0.12, 0.35 ±0.08, 0.68 ±0.04, 0.16 ±0.11, 0.34 ±0.09, 0.30 ±0.05, 1.00 ±0.00, 0.13 ±0.05
MAP2: 0.25 ±0.05, 0.33 ±0.05, 0.19 ±0.04, 0.20 ±0.10, 0.26 ±0.05, 0.20 ±0.05, 0.23 ±0.05, 0.32 ±0.07, 0.13 ±0.05, 1.00 ±0.00
Homer-1b/c cortactin SHANK3 synapsin-1 NR2B actin bassoon PSD-95 VGLUT1 MAP2
1 0.8 0.6 0.4 0.2 0 -0.2 -0.4 -0.6 -0.8 -1

(Continued)

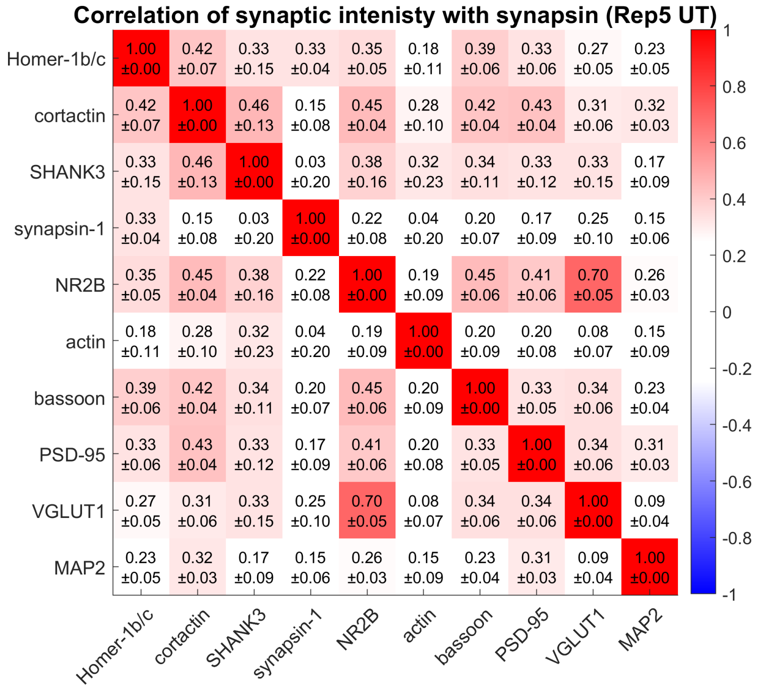
Correlation of synaptic intenisty with synapsin (Rep5 UT)
Homer-1b/c 1.00 ±0.00 0.42 ±0.07 0.33 ±0.15 0.33 ±0.04 0.35 ±0.05 0.18 ±0.11 0.39 ±0.06 0.33 ±0.06 0.27 ±0.05 0.23 ±0.05
cortactin 0.42 ±0.07 1.00 ±0.00 0.46 ±0.13 0.15 ±0.08 0.45 ±0.04 0.28 ±0.10 0.42 ±0.04 0.43 ±0.04 0.31 ±0.06 0.32 ±0.03
SHANK3 0.33 ±0.15 0.46 ±0.13 1.00 ±0.00 0.03 ±0.20 0.38 ±0.16 0.32 ±0.23 0.34 ±0.11 0.33 ±0.12 0.33 ±0.15 0.17 ±0.09
synapsin-1 0.33 ±0.04 0.15 ±0.08 0.03 ±0.20 1.00 ±0.00 0.22 ±0.08 0.04 ±0.20 0.20 ±0.07 0.17 ±0.09 0.25 ±0.10 0.15 ±0.06
NR2B 0.35 ±0.05 0.45 ±0.04 0.38 ±0.16 0.22 ±0.08 1.00 ±0.00 0.19 ±0.09 0.45 ±0.06 0.41 ±0.06 0.70 ±0.05 0.26 ±0.03
actin 0.18 ±0.11 0.28 ±0.10 0.32 ±0.23 0.04 ±0.20 0.19 ±0.09 1.00 ±0.00 0.20 ±0.09 0.20 ±0.08 0.08 ±0.07 0.15 ±0.09
bassoon 0.39 ±0.06 0.42 ±0.04 0.34 ±0.11 0.20 ±0.07 0.45 ±0.06 0.20 ±0.09 1.00 ±0.00 0.33 ±0.05 0.34 ±0.06 0.23 ±0.04
PSD-95 0.33 ±0.06 0.43 ±0.04 0.33 ±0.12 0.17 ±0.09 0.41 ±0.06 0.20 ±0.08 0.33 ±0.05 1.00 ±0.00 0.34 ±0.06 0.31 ±0.03
VGLUT1 0.27 ±0.05 0.31 ±0.06 0.33 ±0.15 0.25 ±0.10 0.70 ±0.05 0.08 ±0.07 0.34 ±0.06 0.34 ±0.06 1.00 ±0.00 0.09 ±0.04
MAP2 0.23 ±0.05 0.32 ±0.03 0.17 ±0.09 0.15 ±0.06 0.26 ±0.03 0.15 ±0.09 0.23 ±0.04 0.31 ±0.03 0.09 ±0.04 1.00 ±0.00
Homer-1b/c cortactin SHANK3 synapsin-1 NR2B actin bassoon PSD-95 VGLUT1 MAP2
1 0.8 0.6 0.4 0.2 0 -0.2 -0.4 -0.6 -0.8 -1

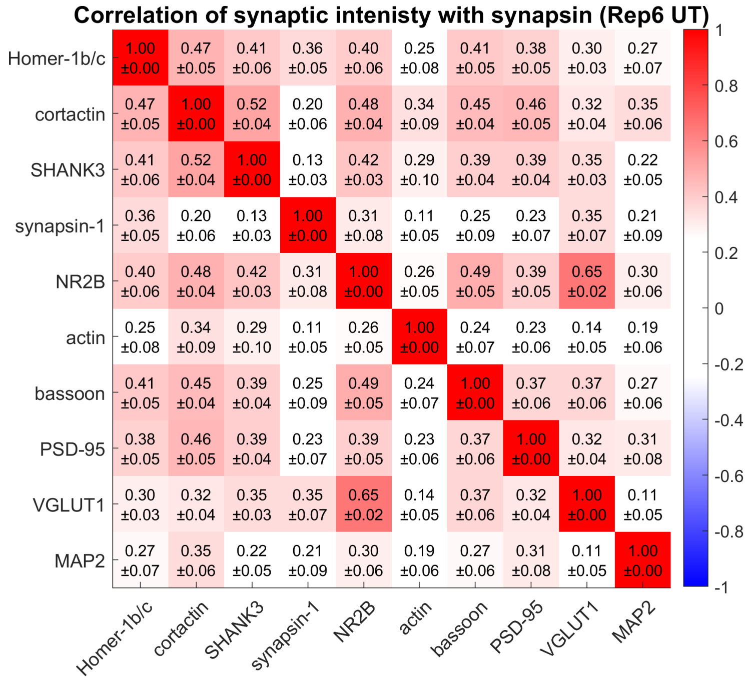
Correlation of synaptic intenisty with synapsin (Rep6 UT)
Homer-1b/c 1.00 ±0.00 0.47 ±0.05 0.41 ±0.06 0.36 ±0.05 0.40 ±0.06 0.25 ±0.08 0.41 ±0.05 0.38 ±0.05 0.30 ±0.03 0.27 ±0.07
cortactin 0.47 ±0.05 1.00 ±0.00 0.52 ±0.04 0.20 ±0.06 0.48 ±0.04 0.34 ±0.09 0.45 ±0.04 0.46 ±0.05 0.32 ±0.04 0.35 ±0.06
SHANK3 0.41 ±0.06 0.52 ±0.04 1.00 ±0.00 0.13 ±0.03 0.42 ±0.03 0.29 ±0.10 0.39 ±0.04 0.39 ±0.04 0.35 ±0.03 0.22 ±0.05
synapsin-1 0.36 ±0.05 0.20 ±0.06 0.13 ±0.03 1.00 ±0.00 0.31 ±0.08 0.11 ±0.05 0.25 ±0.09 0.23 ±0.07 0.35 ±0.07 0.21 ±0.09
NR2B 0.40 ±0.06 0.48 ±0.04 0.42 ±0.03 0.31 ±0.08 1.00 ±0.00 0.26 ±0.05 0.49 ±0.05 0.39 ±0.05 0.65 ±0.02 0.30 ±0.06
actin 0.25 ±0.08 0.34 ±0.09 0.29 ±0.10 0.11 ±0.05 0.26 ±0.05 1.00 ±0.00 0.24 ±0.07 0.23 ±0.06 0.14 ±0.05 0.19 ±0.06
bassoon 0.41 ±0.05 0.45 ±0.04 0.39 ±0.04 0.25 ±0.09 0.49 ±0.05 0.24 ±0.07 1.00 ±0.00 0.37 ±0.06 0.37 ±0.06 0.27 ±0.06
PSD-95 0.38 ±0.05 0.46 ±0.05 0.39 ±0.04 0.23 ±0.07 0.39 ±0.05 0.23 ±0.06 0.37 ±0.06 1.00 ±0.00 0.32 ±0.04 0.31 ±0.08
VGLUT1 0.30 ±0.03 0.32 ±0.04 0.35 ±0.03 0.35 ±0.07 0.65 ±0.02 0.14 ±0.05 0.37 ±0.06 0.32 ±0.04 1.00 ±0.00 0.11 ±0.05
MAP2 0.27 ±0.07 0.35 ±0.06 0.22 ±0.05 0.21 ±0.09 0.30 ±0.06 0.19 ±0.06 0.27 ±0.06 0.31 ±0.08 0.11 ±0.05 1.00 ±0.00
Homer-1b/c cortactin SHANK3 synapsin-1 NR2B actin bassoon PSD-95 VGLUT1 MAP2
1 0.8 0.6 0.4 0.2 0 -0.2 -0.4 -0.6 -0.8 -1

(Continued)

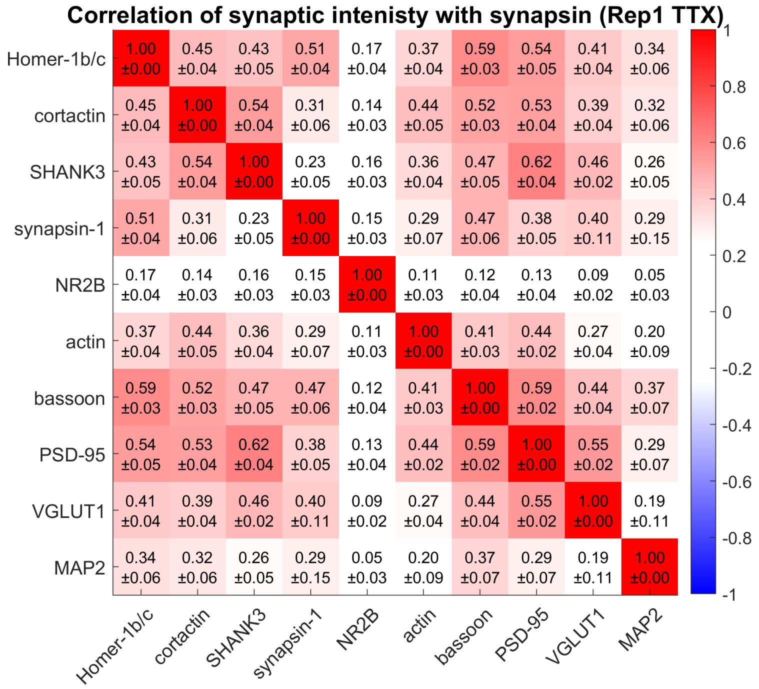
Correlation of synaptic intenisty with synapsin (Rep1 TTX)
Homer-1b/c 1.00 ±0.00 0.45 ±0.04 0.43 ±0.05 0.51 ±0.04 0.17 ±0.04 0.37 ±0.04 0.59 ±0.03 0.54 ±0.05 0.41 ±0.04 0.34 ±0.06
cortactin 0.45 ±0.04 1.00 ±0.00 0.54 ±0.04 0.31 ±0.06 0.14 ±0.03 0.44 ±0.05 0.52 ±0.03 0.53 ±0.04 0.39 ±0.04 0.32 ±0.06
SHANK3 0.43 ±0.05 0.54 ±0.04 1.00 ±0.00 0.23 ±0.05 0.16 ±0.03 0.36 ±0.04 0.47 ±0.05 0.62 ±0.04 0.46 ±0.02 0.26 ±0.05
synapsin-1 0.51 ±0.04 0.31 ±0.06 0.23 ±0.05 1.00 ±0.00 0.15 ±0.03 0.29 ±0.07 0.47 ±0.06 0.38 ±0.05 0.40 ±0.11 0.29 ±0.15
NR2B 0.17 ±0.04 0.14 ±0.03 0.16 ±0.03 0.15 ±0.03 1.00 ±0.00 0.11 ±0.03 0.12 ±0.04 0.13 ±0.04 0.09 ±0.02 0.05 ±0.03
actin 0.37 ±0.04 0.44 ±0.05 0.36 ±0.04 0.29 ±0.07 0.11 ±0.03 1.00 ±0.00 0.41 ±0.03 0.44 ±0.02 0.27 ±0.04 0.20 ±0.09
bassoon 0.59 ±0.03 0.52 ±0.03 0.47 ±0.05 0.47 ±0.06 0.12 ±0.04 0.41 ±0.03 1.00 ±0.00 0.59 ±0.02 0.44 ±0.04 0.37 ±0.07
PSD-95 0.54 ±0.05 0.53 ±0.04 0.62 ±0.04 0.38 ±0.05 0.13 ±0.04 0.44 ±0.02 0.59 ±0.02 1.00 ±0.00 0.55 ±0.02 0.29 ±0.07
VGLUT1 0.41 ±0.04 0.39 ±0.04 0.46 ±0.02 0.40 ±0.11 0.09 ±0.02 0.27 ±0.04 0.44 ±0.04 0.55 ±0.02 1.00 ±0.00 0.19 ±0.11
MAP2 0.34 ±0.06 0.32 ±0.06 0.26 ±0.05 0.29 ±0.15 0.05 ±0.03 0.20 ±0.09 0.37 ±0.07 0.29 ±0.07 0.19 ±0.11 1.00 ±0.00
Homer-1b/c cortactin SHANK3 synapsin-1 NR2B actin bassoon PSD-95 VGLUT1 MAP2
1 0.8 0.6 0.4 0.2 0 -0.2 -0.4 -0.6 -0.8 -1

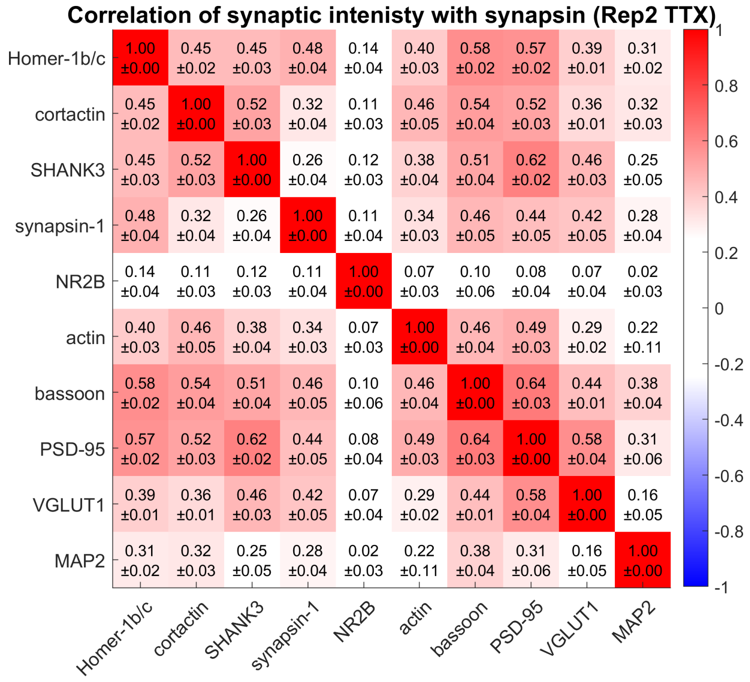
Correlation of synaptic intenisty with synapsin (Rep2 TTX)
Homer-1b/c 1.00 ±0.00 0.45 ±0.02 0.45 ±0.03 0.48 ±0.04 0.14 ±0.04 0.40 ±0.03 0.58 ±0.02 0.57 ±0.02 0.39 ±0.01 0.31 ±0.02
cortactin 0.45 ±0.02 1.00 ±0.00 0.52 ±0.03 0.32 ±0.04 0.11 ±0.03 0.46 ±0.05 0.54 ±0.04 0.52 ±0.03 0.36 ±0.01 0.32 ±0.03
SHANK3 0.45 ±0.03 0.52 ±0.03 1.00 ±0.00 0.26 ±0.04 0.12 ±0.03 0.38 ±0.04 0.51 ±0.04 0.62 ±0.02 0.46 ±0.03 0.25 ±0.05
synapsin-1 0.48 ±0.04 0.32 ±0.04 0.26 ±0.04 1.00 ±0.00 0.11 ±0.04 0.34 ±0.03 0.46 ±0.05 0.44 ±0.05 0.42 ±0.05 0.28 ±0.04
NR2B 0.14 ±0.04 0.11 ±0.03 0.12 ±0.03 0.11 ±0.04 1.00 ±0.00 0.07 ±0.03 0.10 ±0.06 0.08 ±0.04 0.07 ±0.04 0.02 ±0.03
actin 0.40 ±0.03 0.46 ±0.05 0.38 ±0.04 0.34 ±0.03 0.07 ±0.03 1.00 ±0.00 0.46 ±0.04 0.49 ±0.03 0.29 ±0.02 0.22 ±0.11
bassoon 0.58 ±0.02 0.54 ±0.04 0.51 ±0.04 0.46 ±0.05 0.10 ±0.06 0.46 ±0.04 1.00 ±0.00 0.64 ±0.03 0.44 ±0.01 0.38 ±0.04
PSD-95 0.57 ±0.02 0.52 ±0.03 0.62 ±0.02 0.44 ±0.05 0.08 ±0.04 0.49 ±0.03 0.64 ±0.03 1.00 ±0.00 0.58 ±0.04 0.31 ±0.06
VGLUT1 0.39 ±0.01 0.36 ±0.01 0.46 ±0.03 0.42 ±0.05 0.07 ±0.04 0.29 ±0.02 0.44 ±0.01 0.58 ±0.04 1.00 ±0.00 0.16 ±0.05
MAP2 0.31 ±0.02 0.32 ±0.03 0.25 ±0.05 0.28 ±0.04 0.02 ±0.03 0.22 ±0.11 0.38 ±0.04 0.31 ±0.06 0.16 ±0.05 1.00 ±0.00
Homer-1b/c cortactin SHANK3 synapsin-1 NR2B actin bassoon PSD-95 VGLUT1 MAP2
1 0.8 0.6 0.4 0.2 0 -0.2 -0.4 -0.6 -0.8 -1

(Continued)

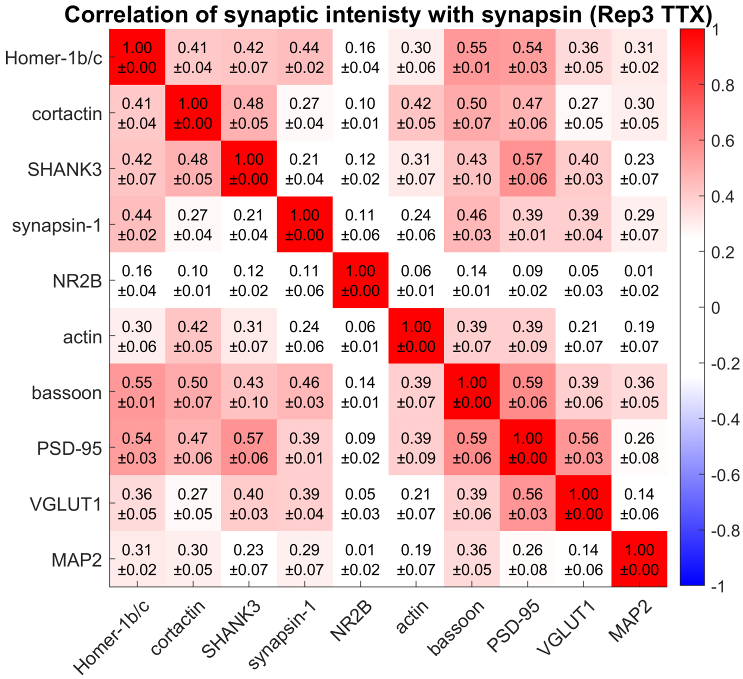
Correlation of synaptic intenisty with synapsin (Rep3 TTX)
Homer-1b/c 1.00 ±0.00 0.41 ±0.04 0.42 ±0.07 0.44 ±0.02 0.16 ±0.04 0.30 ±0.06 0.55 ±0.01 0.54 ±0.03 0.36 ±0.05 0.31 ±0.02
cortactin 0.41 ±0.04 1.00 ±0.00 0.48 ±0.05 0.27 ±0.04 0.10 ±0.01 0.42 ±0.05 0.50 ±0.07 0.47 ±0.06 0.27 ±0.05 0.30 ±0.05
SHANK3 0.42 ±0.07 0.48 ±0.05 1.00 ±0.00 0.21 ±0.04 0.12 ±0.02 0.31 ±0.07 0.43 ±0.10 0.57 ±0.06 0.40 ±0.03 0.23 ±0.07
synapsin-1 0.44 ±0.02 0.27 ±0.04 0.21 ±0.04 1.00 ±0.00 0.11 ±0.06 0.24 ±0.06 0.46 ±0.03 0.39 ±0.01 0.39 ±0.04 0.29 ±0.07
NR2B 0.16 ±0.04 0.10 ±0.01 0.12 ±0.02 0.11 ±0.06 1.00 ±0.00 0.06 ±0.01 0.14 ±0.01 0.09 ±0.02 0.05 ±0.03 0.01 ±0.02
actin 0.30 ±0.06 0.42 ±0.05 0.31 ±0.07 0.24 ±0.06 0.06 ±0.01 1.00 ±0.00 0.39 ±0.07 0.39 ±0.09 0.21 ±0.07 0.19 ±0.07
bassoon 0.55 ±0.01 0.50 ±0.07 0.43 ±0.10 0.46 ±0.03 0.14 ±0.01 0.39 ±0.07 1.00 ±0.00 0.59 ±0.06 0.39 ±0.06 0.36 ±0.05
PSD-95 0.54 ±0.03 0.47 ±0.06 0.57 ±0.06 0.39 ±0.01 0.09 ±0.02 0.39 ±0.09 0.59 ±0.06 1.00 ±0.00 0.56 ±0.03 0.26 ±0.08
VGLUT1 0.36 ±0.05 0.27 ±0.05 0.40 ±0.03 0.39 ±0.04 0.05 ±0.03 0.21 ±0.07 0.39 ±0.06 0.56 ±0.03 1.00 ±0.00 0.14 ±0.06
MAP2 0.31 ±0.02 0.30 ±0.05 0.23 ±0.07 0.29 ±0.07 0.01 ±0.02 0.19 ±0.07 0.36 ±0.05 0.26 ±0.08 0.14 ±0.06 1.00 ±0.00
Homer-1b/c cortactin SHANK3 synapsin-1 NR2B actin bassoon PSD-95 VGLUT1 MAP2
1 0.8 0.6 0.4 0.2 0 -0.2 -0.4 -0.6 -0.8 -1

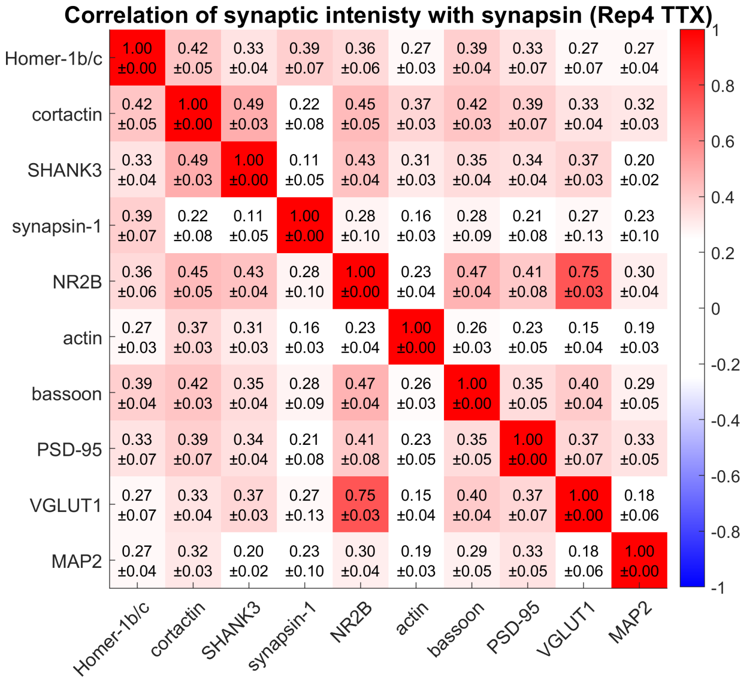
Correlation of synaptic intenisty with synapsin (Rep4 TTX)
Homer-1b/c 1.00 ±0.00 0.42 ±0.05 0.33 ±0.04 0.39 ±0.07 0.36 ±0.06 0.27 ±0.03 0.39 ±0.04 0.33 ±0.07 0.27 ±0.07 0.27 ±0.04
cortactin 0.42 ±0.05 1.00 ±0.00 0.49 ±0.03 0.22 ±0.08 0.45 ±0.05 0.37 ±0.03 0.42 ±0.03 0.39 ±0.07 0.33 ±0.04 0.32 ±0.03
SHANK3 0.33 ±0.04 0.49 ±0.03 1.00 ±0.00 0.11 ±0.05 0.43 ±0.04 0.31 ±0.03 0.35 ±0.04 0.34 ±0.04 0.37 ±0.03 0.20 ±0.02
synapsin-1 0.39 ±0.07 0.22 ±0.08 0.11 ±0.05 1.00 ±0.00 0.28 ±0.10 0.16 ±0.03 0.28 ±0.09 0.21 ±0.08 0.27 ±0.13 0.23 ±0.10
NR2B 0.36 ±0.06 0.45 ±0.05 0.43 ±0.04 0.28 ±0.10 1.00 ±0.00 0.23 ±0.04 0.47 ±0.04 0.41 ±0.08 0.75 ±0.03 0.30 ±0.04
actin 0.27 ±0.03 0.37 ±0.03 0.31 ±0.03 0.16 ±0.03 0.23 ±0.04 1.00 ±0.00 0.26 ±0.03 0.23 ±0.05 0.15 ±0.04 0.19 ±0.03
bassoon 0.39 ±0.04 0.42 ±0.03 0.35 ±0.04 0.28 ±0.09 0.47 ±0.04 0.26 ±0.03 1.00 ±0.00 0.35 ±0.05 0.40 ±0.04 0.29 ±0.05
PSD-95 0.33 ±0.07 0.39 ±0.07 0.34 ±0.04 0.21 ±0.08 0.41 ±0.08 0.23 ±0.05 0.35 ±0.05 1.00 ±0.00 0.37 ±0.07 0.33 ±0.05
VGLUT1 0.27 ±0.07 0.33 ±0.04 0.37 ±0.03 0.27 ±0.13 0.75 ±0.03 0.15 ±0.04 0.40 ±0.04 0.37 ±0.07 1.00 ±0.00 0.18 ±0.06
MAP2 0.27 ±0.04 0.32 ±0.03 0.20 ±0.02 0.23 ±0.10 0.30 ±0.04 0.19 ±0.03 0.29 ±0.05 0.33 ±0.05 0.18 ±0.06 1.00 ±0.00
Homer-1b/c cortactin SHANK3 synapsin-1 NR2B actin bassoon PSD-95 VGLUT1 MAP2
1 0.8 0.6 0.4 0.2 0 -0.2 -0.4 -0.6 -0.8 -1

(Continued)

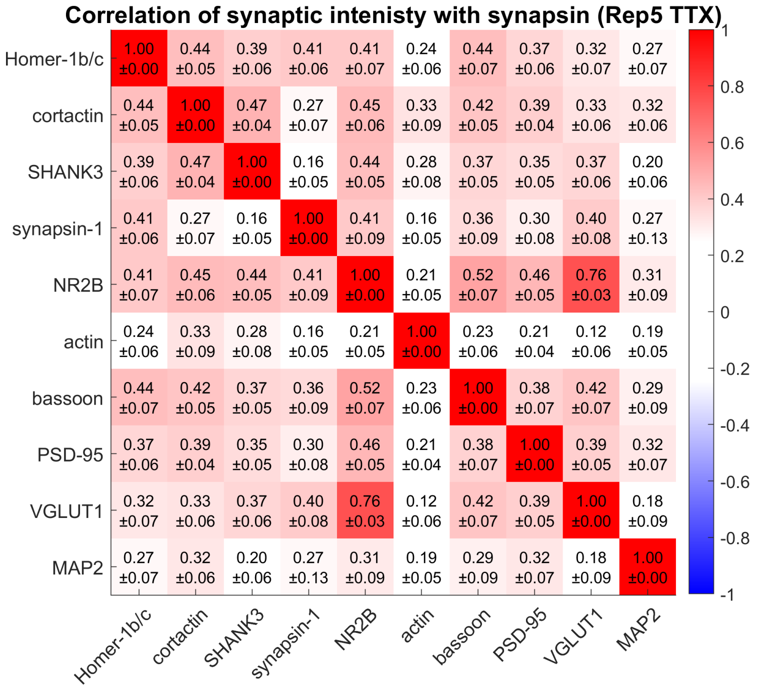

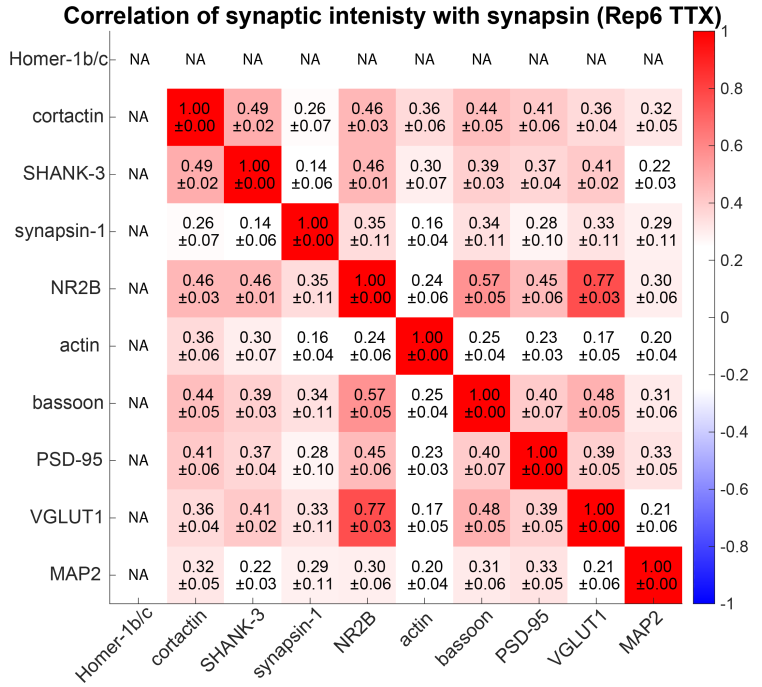

Supplementary Figure 21. PCC between intensity levels of synaptic proteins within synapses. Correlation matrices for six replicates are shown. NA indicates no data available.

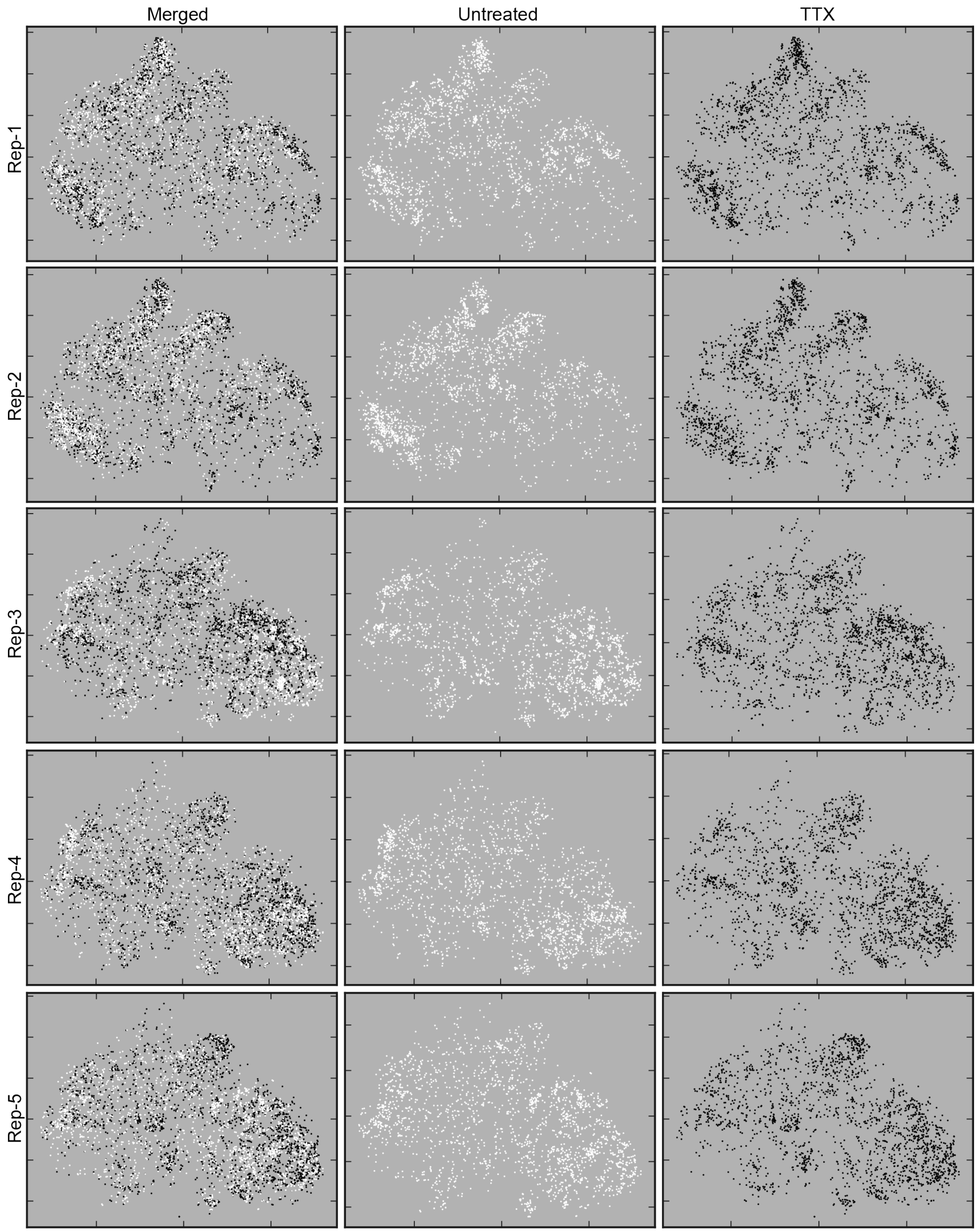

Supplementary Figure 22. t-SNE plots for each individual replicate. 4000 synapses were randomly chosen from each replicate. 2000 from Untreated (white) samples and 2000 from TTX (black) treated samples. Each point represents an individual synapse.

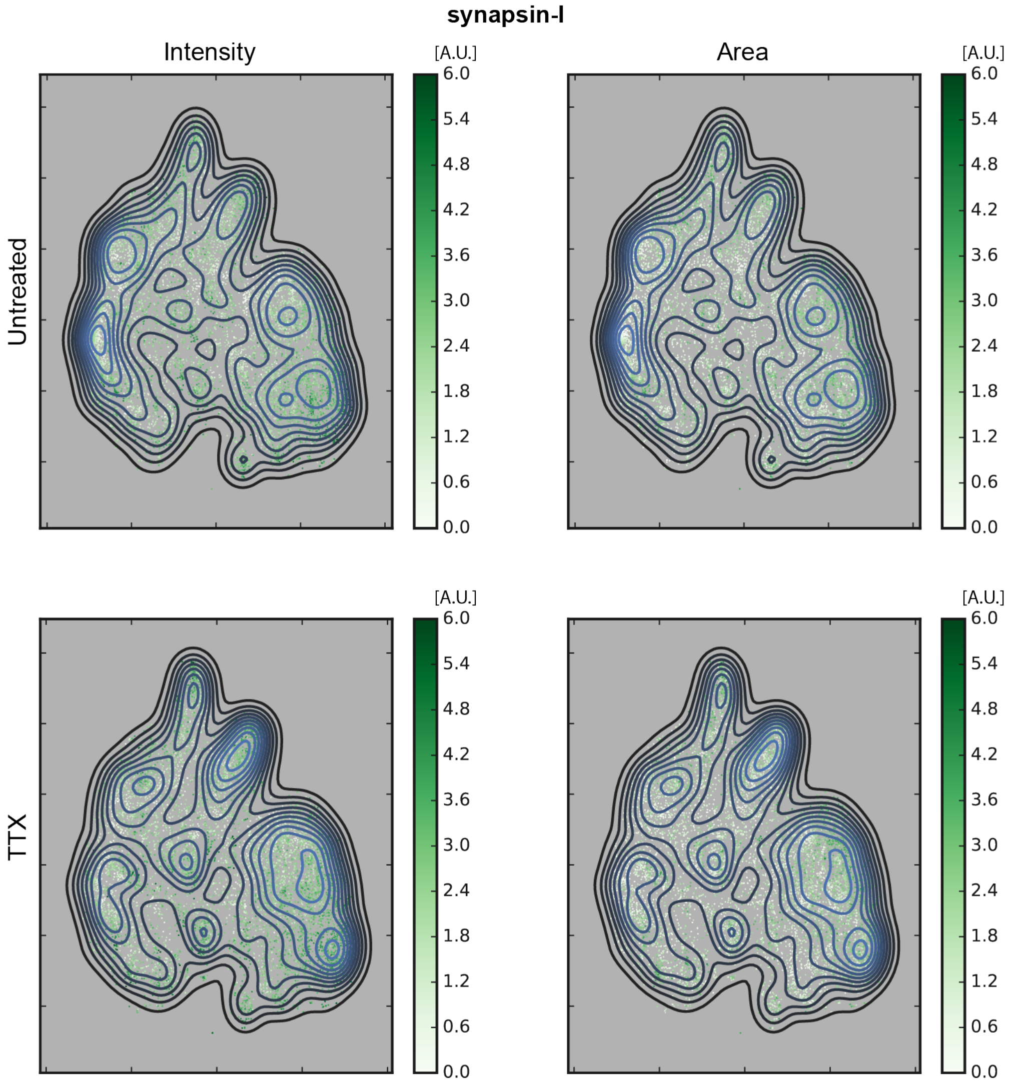

Supplementary Figure 23. t-SNE plot with synaptic density contour plot colored for synapsin-I Intensity (left) or Area (right). Each point represents an individual synapse.

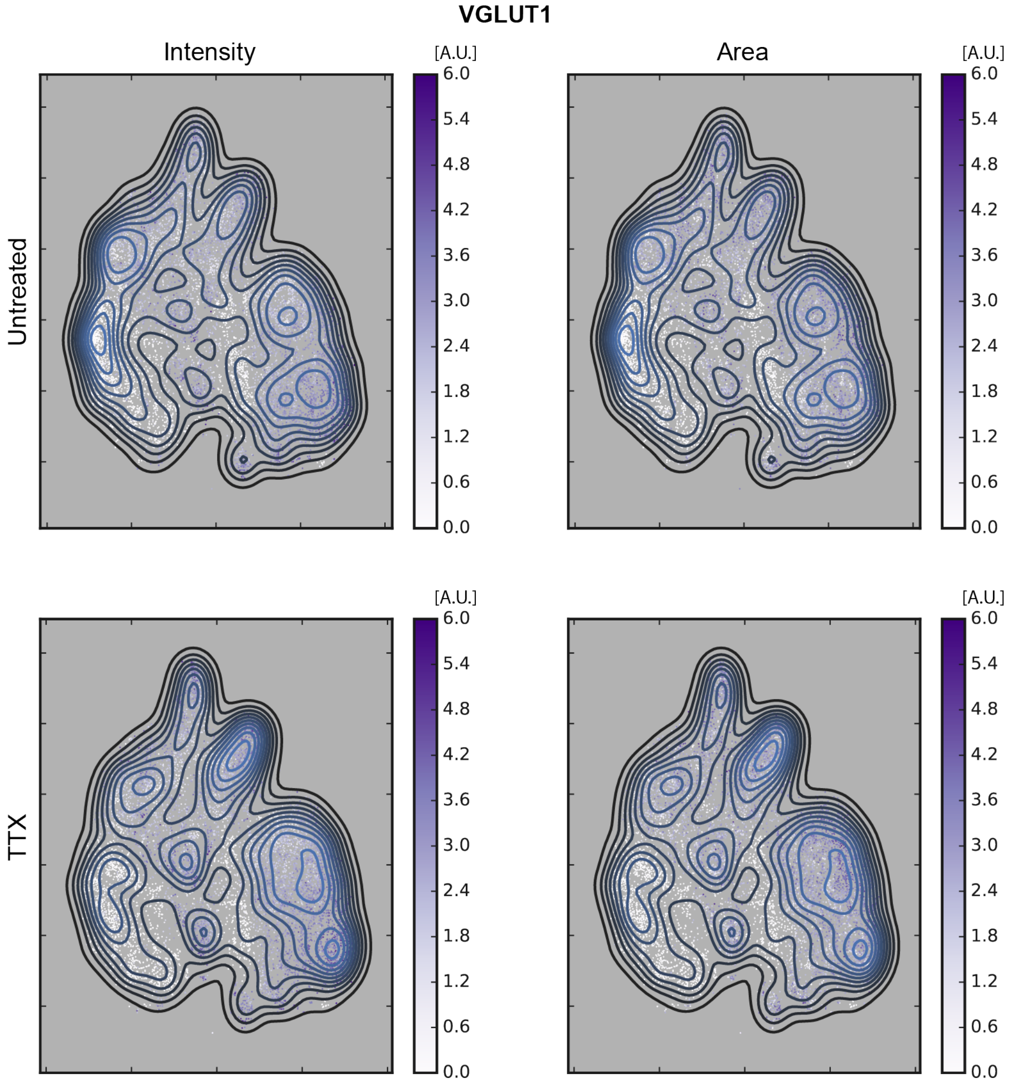

Supplementary Figure 24. t-SNE plot with synaptic density contour plot colored for VGLUT1 Intensity (left) or Area (right). Each point represents an individual synapse.

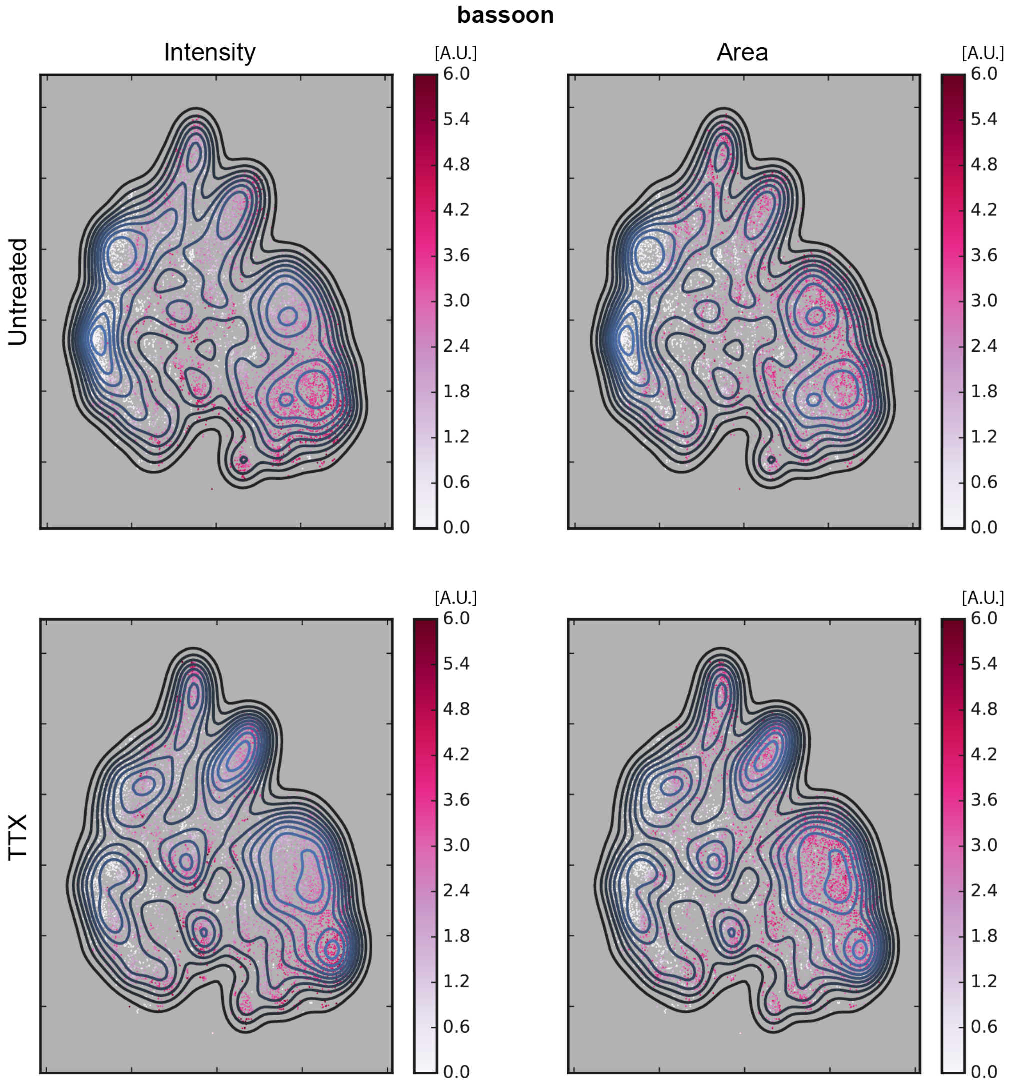

Supplementary Figure 25. t-SNE plot with synaptic density contour plot colored for bassoon Intensity (left) or Area (right). Each point represents an individual synapse.

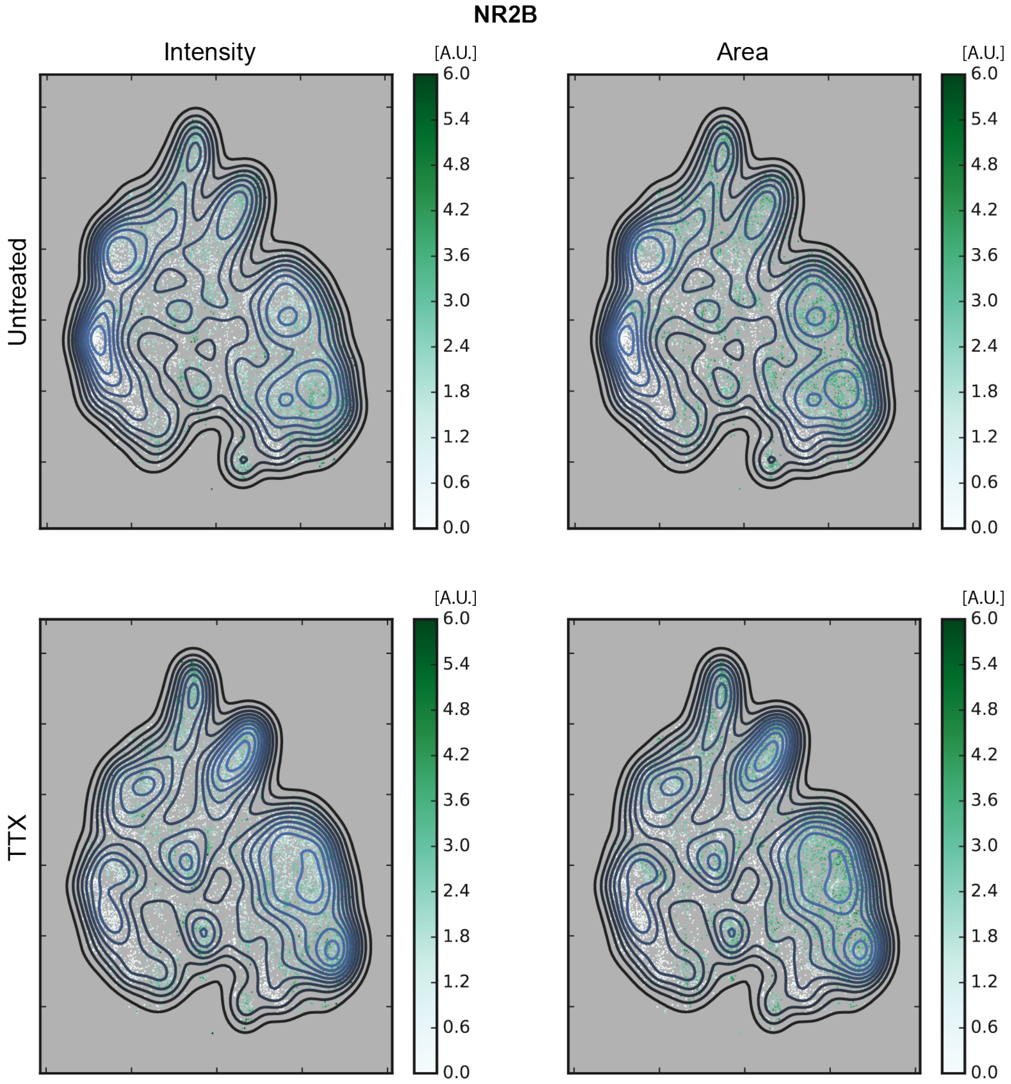

Supplementary Figure 26. t-SNE plot with synaptic density contour plot colored for NR2B Intensity (left) or Area (right). Each point represents an individual synapse.

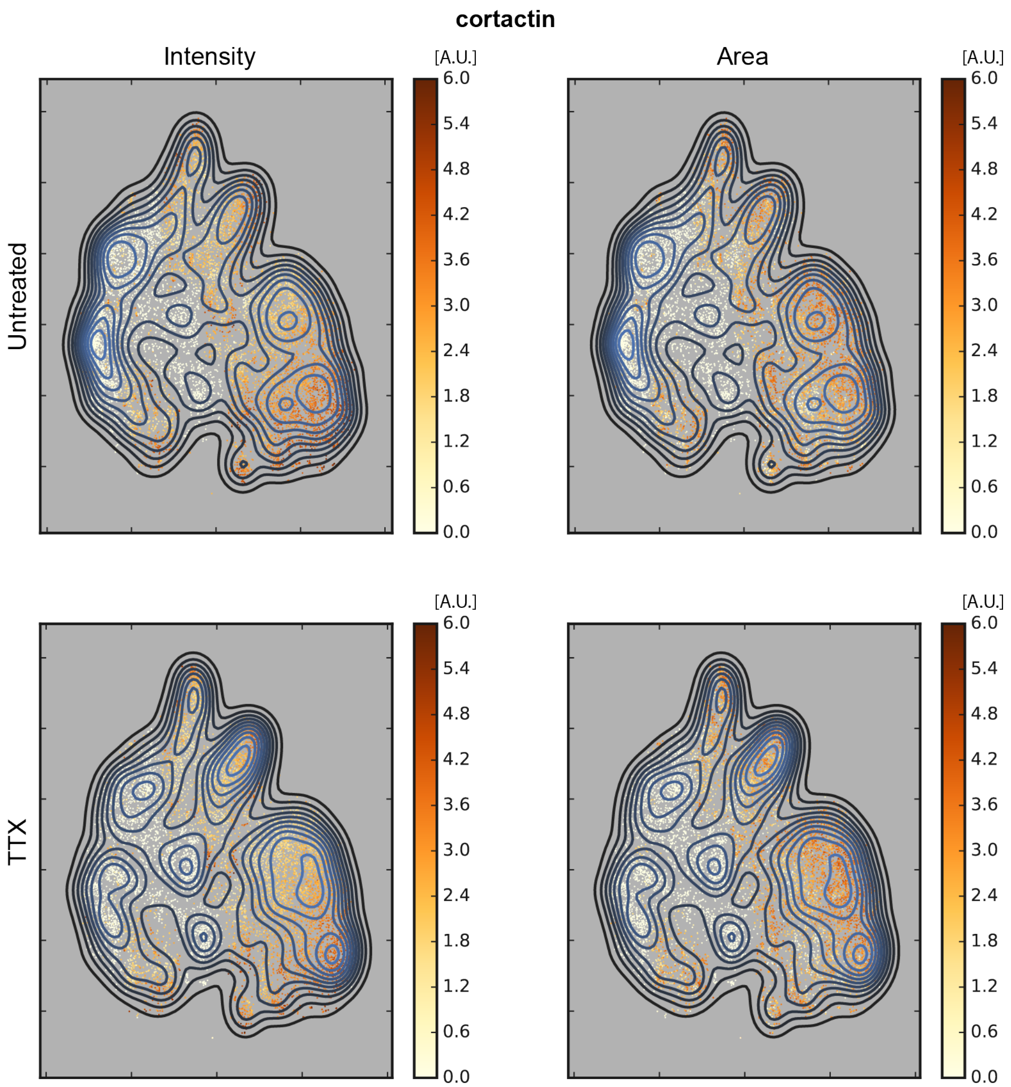

Supplementary Figure 27. t-SNE plot with synaptic density contour plot colored for cortactin Intensity (left) or Area (right). Each point represents an individual synapse.

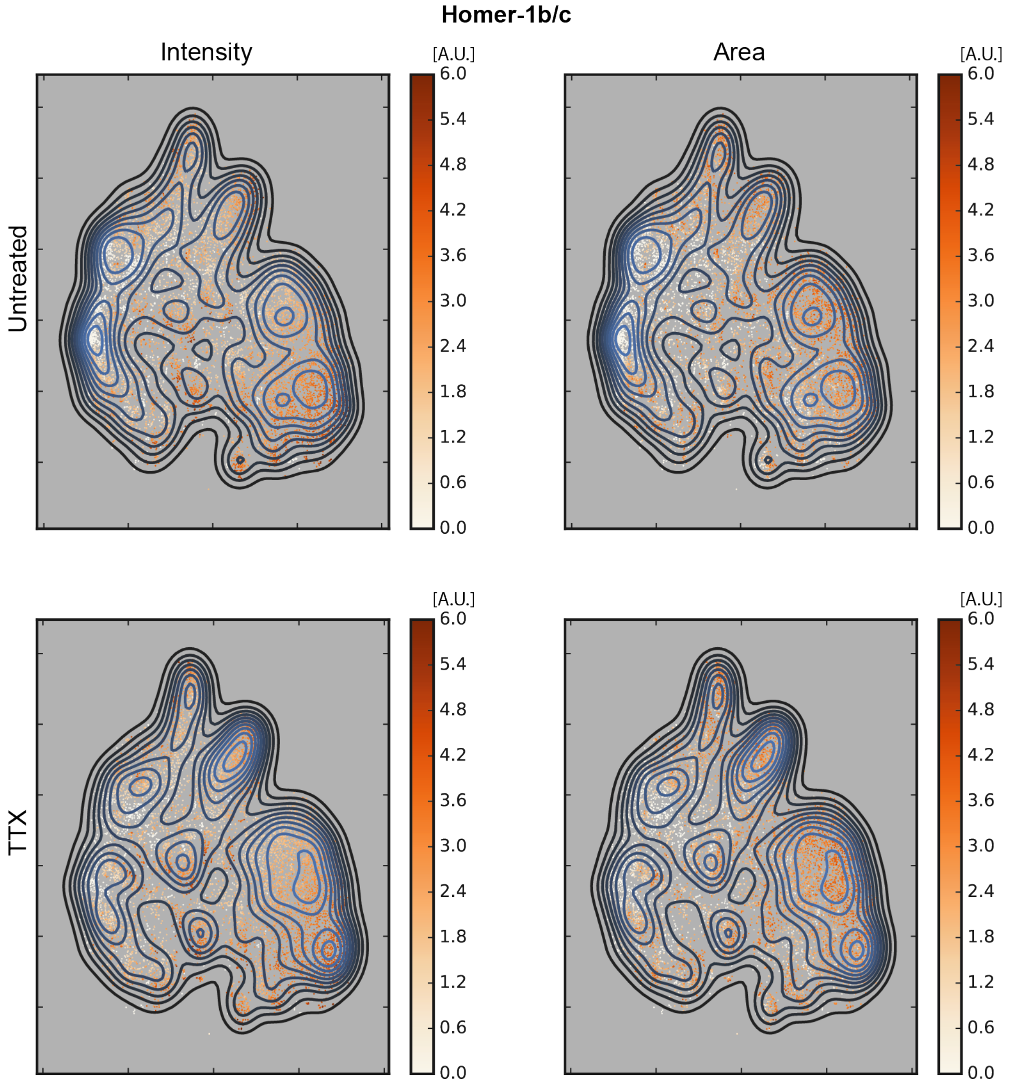

Supplementary Figure 28. t-SNE plot with synaptic density contour plot colored for Homer1b/c Intensity (left) or Area (right). Each point represents an individual synapse.

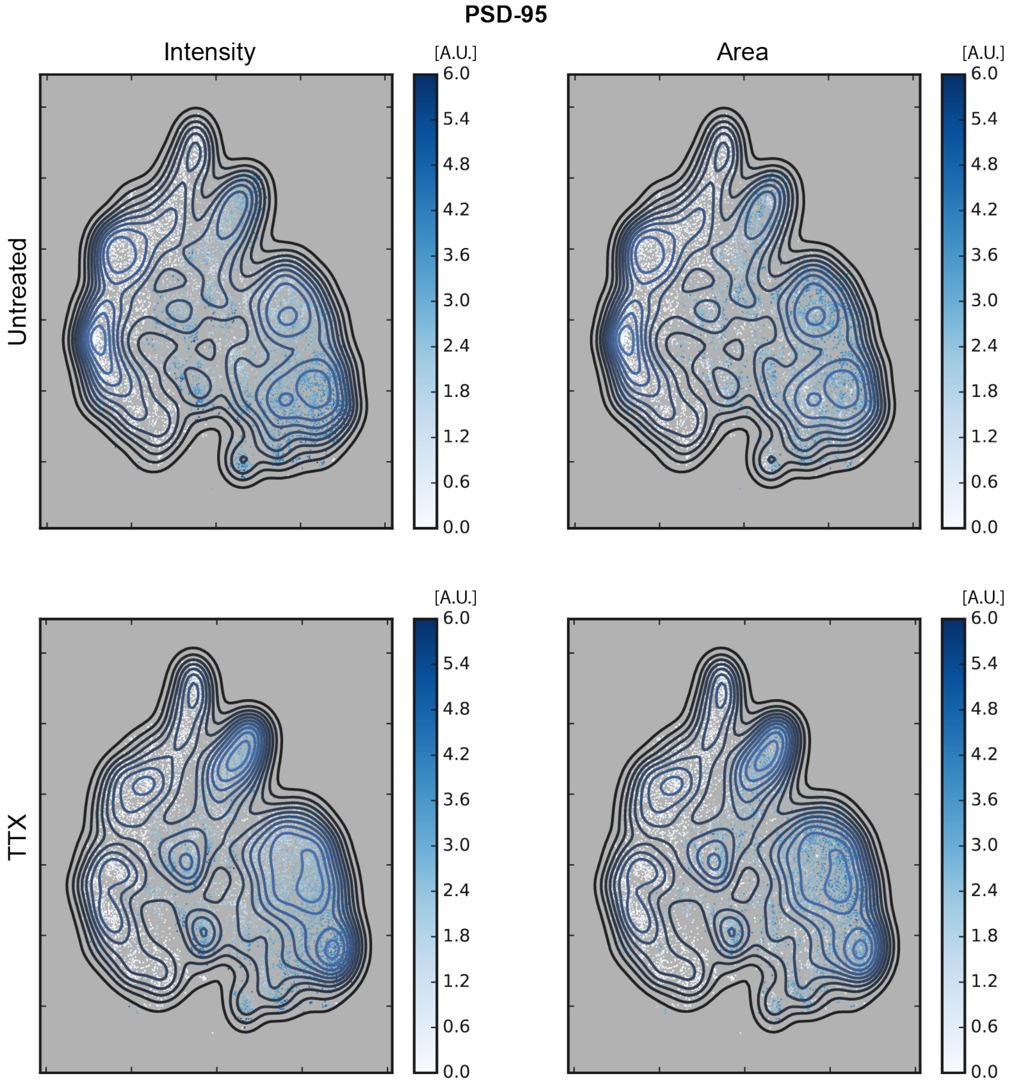

Supplementary Figure 29. t-SNE plot with synaptic density contour plot colored for PSD-95 Intensity (left) or Area (right). Each point represents an individual synapse.

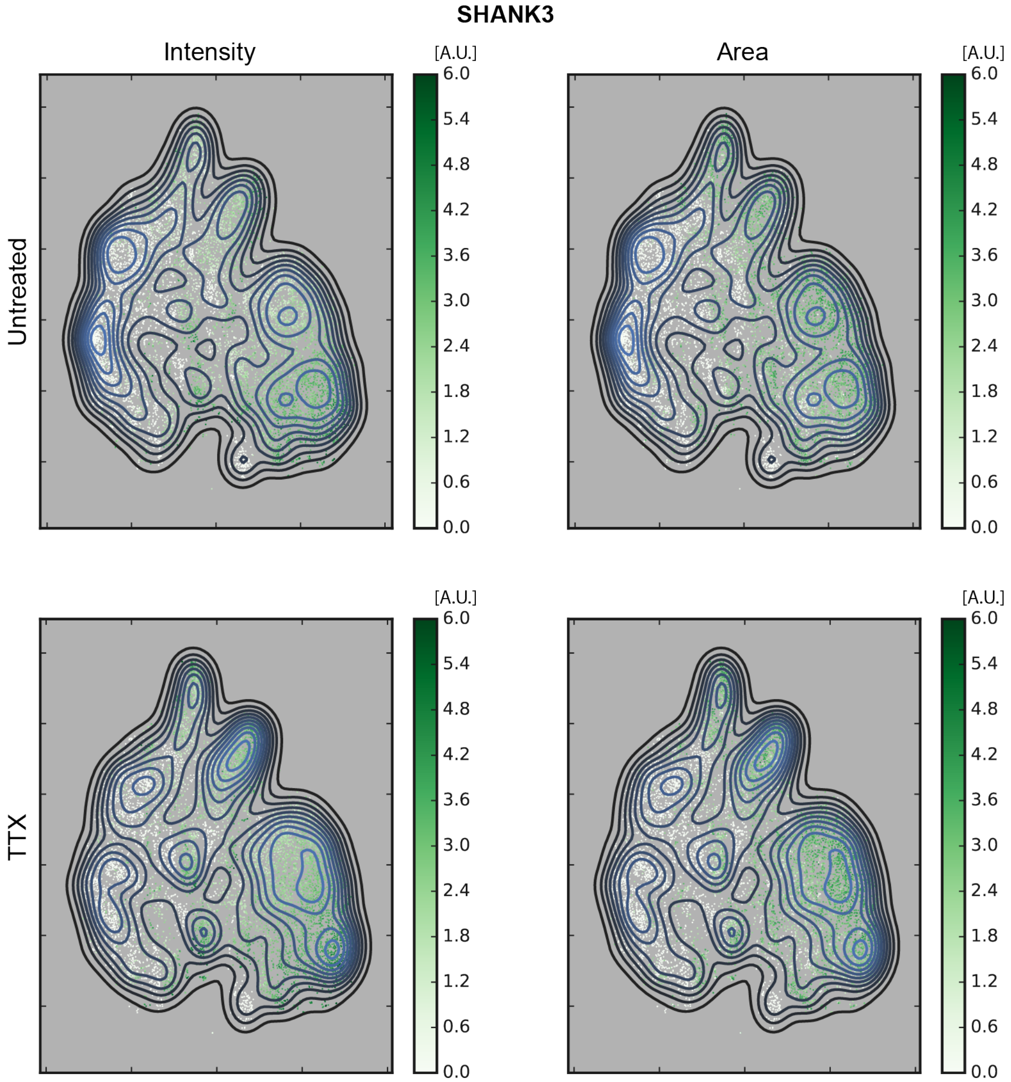

Supplementary Figure 30. t-SNE plot with synaptic density contour plot colored for SHANK3 Intensity (left) or Area (right). Each point represents an individual synapse.

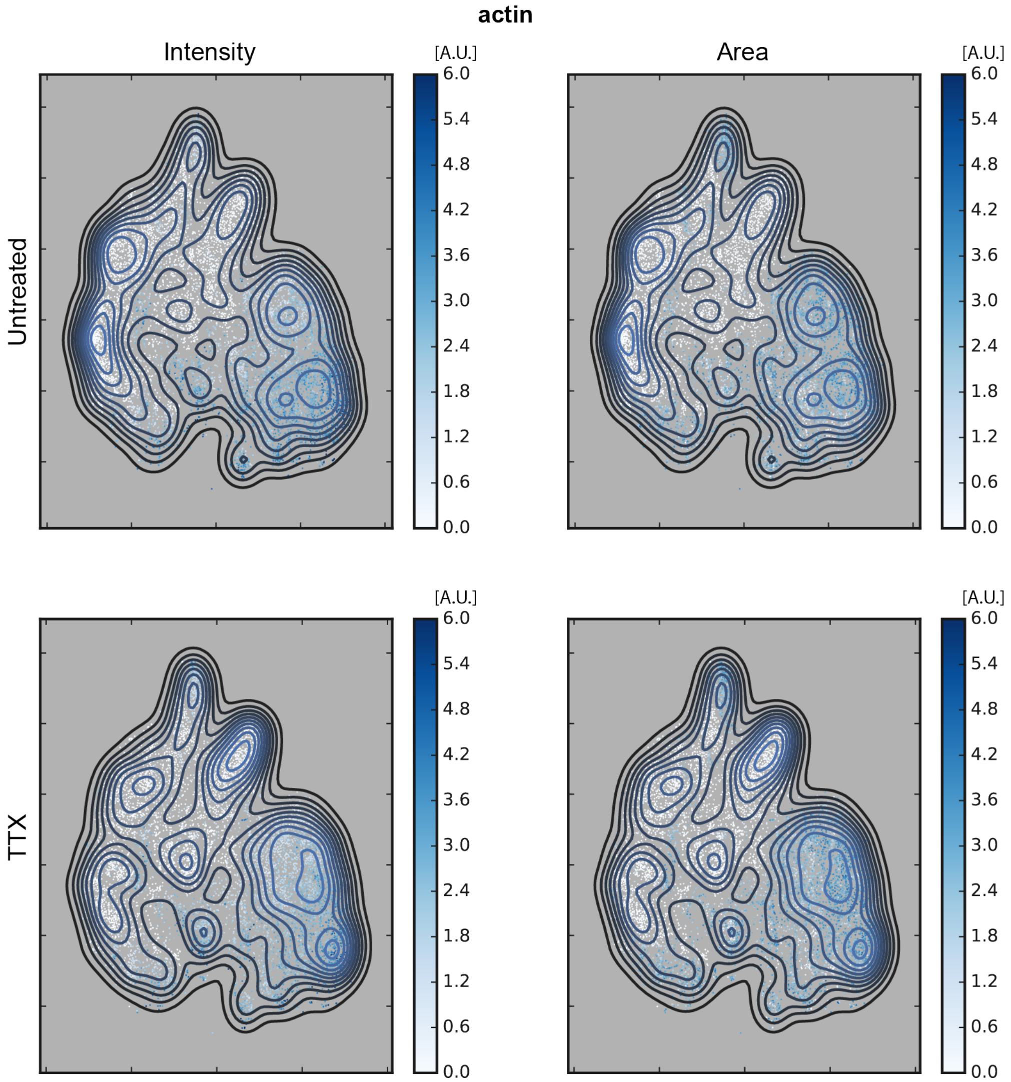

Supplementary Figure 31. t-SNE plot with synaptic density contour plot colored for actin Intensity (left) or Area (right). Each point represents an individual synapse.

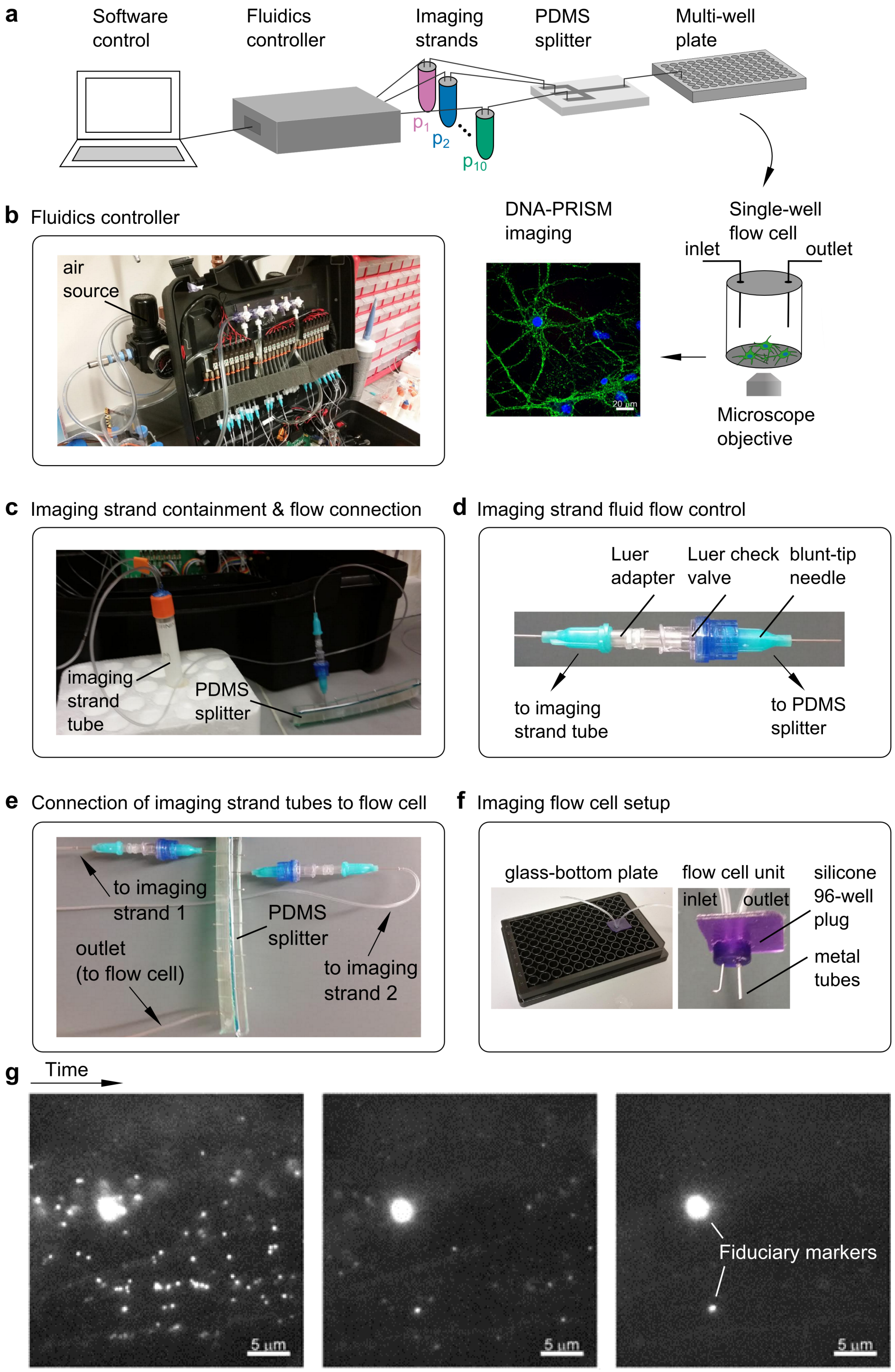

Supplementary Figure 32. Fluidics system for automated multiplexed imaging using DNA-PRISM. (a) Schematic of the system components, connections, and imaging setup. (b) A portable briefcase houses the solenoid valves that selectively control air flow. These valves are regulated by a microcontroller that is operated by computer software via a USB

port. An external air source with regulated pressure feeds into the solenoids. (c) A tube containing solution of a single fluorophore-conjugated nucleic acid oligo sequence (imaging probe/strand) diluted in imaging buffer is connected at the inlet to a single solenoid valve, which can be shut on/off by the computer software. The outlet is connected to a check value assembly (d) through blunt-tip needles and Luer adapters, ensuring that imaging strand flows only in one direction toward the sample. (e) Multiple check valve assemblies are connected to a PDMS splitter with a thin, long channel, which enables multiple imaging strands to feed into a single outlet. The splitter outlet connects with the imaging flow cell. (f) A flow cell is assembled within a well of a standard glass-bottom 96-well plate that contains fixed cells stained for all molecular targets of interest. The flow cell unit is assembled using a silicone plug that fits tightly into a well of the 96-well plate and contains an inlet and outlet for flowing imaging strand or wash buffer in and out. (g) Wash-out of ssDNA imaging strand using the fluidics system. Snapshot of DNA-PRISM imaging of synapsin-I before washing (left), after two minutes of washing (middle), and after five minutes of washing (right) using the fluidics system described. The fiduciary markers (two remaining bright spots in the right panel that are gold nanoparticles used for super-resolution image reconstruction) are not washed out at the input pressure used (flow rates generated at 5-20 psi at the air source are sufficient for imaging experiments in practice).

Supplementary Table 1. Neuronal markers and their concentrations used for staining.

**Antibody target**	**Target Type**	**Vendor**	**Cat No.**	**Species and Clonality**	**Conjugation Strategy**	**Docking Strand Sequence**	**Working Concentration [µg/mL]**
PSD-95	post-synaptic	Cell Signaling Technology	3450	rabbit monoclonal	–	–	0.1
Bassoon	pre-synaptic	Enzo Life Sciences	ADI-VAM-PS003	mouse monoclonal	SMCC	P8	4
Bassoon	pre-synaptic	Enzo Life Sciences	ADI-VAM-PS003	mouse monoclonal	SiteClick	p5(PNA)	3.4
MAP2	dendritic	Novus Biologicals	NB300-213	chicken polyclonal	–	–	
Tuj-1	cytoskeletal	Sigma	T5076	mouse monoclonal	SMCC	p3	2.5
Phalloidin-NH2	cytoskeletal	Bachem	H-7634	–	SMCC	p2	50
ARPC2	cytoskeletal	Millipore	07-227	rabbit polyclonal	SMCC	p7	14
Cortactin	cytoskeletal	Millipore	05-180	mouse monoclonal	SMCC	p4	20
synapsin-I	pre-synaptic	Santa Cruz	sc-7379	goat polyclonal	SMCC	p9	3
SHANK3	post-synaptic	Santa Cruz	sc-30193	rabbit polyclonal	SiteClick	p6	7.4
Homer-1b/c	post-synaptic	Santa Cruz	sc-20807	rabbit polyclonal	SMCC	p10	4
NR2B	receptor	NeuroMab	75-097	mouse monoclonal	–	–	10
anti-rabbit secondary	Rabbit IgG	Life Technologies	A16126	goat polyclonal	SMCC	p1	3
anti-mouse secondary	Mouse IgG	Life Technologies	A16068	goat polyclonal	SMCC	p12	3
MAP2	dendritic	Abcam	Ab5392	chicken polyclonal	–	–	9.5
VGLUT1	pre-synaptic	Synaptic Systems	135304	guinea pig polyclonal	–	–	1:400 (dilution)
Alexa 488 anti-chicken secondary	Chicken IgY	Thermo Fisher	A11039	goat polyclonal	–	–	4
Alexa 555 anti-Guinea Pig secondary	Guinea Pig IgG	Thermo Fisher	A21435	goat polyclonal	–	–	4

Supplementary Table 2. Docking strand and imaging probe sequences used in PRISM.

**Sequence Name**	**Docking Strand Sequence (5' to 3')**	**ssLNA Imaging Probe Sequence (5' to 3')**	**ssDNA Imaging Probe Sequence (5' to 3')**
p1	TTATACATCTA	TAGATGTATAA	CTAGATGTAT
p2	TTATCTACATA	TATGTAGATAA	TATGTAGATC
p3	TTTCTTCATTA	TAATGAAGAAA	GTAATGAAGA
p4	TTATGAATCTA	TAGATTCATAA	GTAGATTCAT
p5	TAGGTAA (PNA)	-	ACCTA
p6	TTAATTGAGTA	TACTCAATTAA	GTACTCAATT
p7	TTAATTAGGAT	ATCCTAATTAA	CATCCTAATT
p8	TTATAATGGAT	ATCCATTATAA	GATCCATTAT
p9	TTTAATAAGGT	ACCTTATTAAA	CACCTTATTA
p10	TTATAGAGAAG	CTTCTCTATAA	CCTTCTCTAT
p12	TTATAGTGATT	AATCACTATAA	GAATCACTAT

LNA nucleotides are colored blue.

Supplementary Table 3. Neuronal markers and their concentrations used for staining.

Antibody target	Target Type	Vendor	Cat No.	Species and Clonality	Conjugation Strategy	Docking Strand Sequence	Working Concentration [µg/mL]
PSD-95	post-synaptic	Cell Signaling Technology	3450	rabbit monoclonal	–	–	0.1
Bassoon	pre-synaptic	Enzo Life Sciences	ADI-VAM-PS003	mouse monoclonal	SMCC	P8	0.65
MAP2	dendritic	Novus Biologicals	NB300-213	chicken polyclonal	–	–	
Phalloidin-NH2	cytoskeletal	Bachem	H-7634	–	SMCC	p2	2.6
Cortactin	cytoskeletal	Millipore	05-180	mouse monoclonal	SMCC	p4	20
synapsin-I	pre-synaptic	Santa Cruz	sc-7379	goat polyclonal	SMCC	p9	5
SHANK3	post-synaptic	Santa Cruz	sc-30193	rabbit polyclonal	SMCC	p6	20
Homer-1b/c	post-synaptic	Santa Cruz	sc-20807	rabbit polyclonal	SMCC	p10	10
NR2B	receptor	NeuroMab	75-097	mouse monoclonal	–	–	10
anti-rabbit secondary	Rabbit IgG	Life Technologies	A16126	goat polyclonal	SMCC	p1	13
anti-mouse secondary	Mouse IgG	Life Technologies	A16068	goat polyclonal	SMCC	p12	3
MAP2	dendritic	Abcam	Ab5392	chicken polyclonal	–	–	9.5
VGLUT1	pre-synaptic	Synaptic Systems	135304	guinea pig polyclonal	–	–	1:400 (dilution)
VGAT	pre-synaptic	Synaptic Systems	131011	mouse monoclonal	SMCC	P3	10
Gephyrin	post-synaptic	Synaptic Systems	147208	rat monoclonal	–	–	0.5
anti-rat secondary	Goat IgG	Invitrogen	A16126	goat polyclonal	SMCC	P7	7.6
Alexa 488 anti-chicken secondary	Chicken IgY	Thermo Fisher	A11039	goat polyclonal	–	–	4
Alexa 555 anti-Guinea Pig secondary	Guinea Pig IgG	Thermo Fisher	A21435	goat polyclonal	–	–	4
