# Supplementary material for: Multiplexed and high-throughput neuronal fluorescence imaging with diffusible probes
Source: Nat Commun. 2019 Sep 26;10:4377. doi: 10.1038/s41467-019-12372-6 (PMC6763432; doi:10.1038/s41467-019-12372-6)
Supplement: Supplementary file 3 — Description of Additional Supplementary Files [file 41467_2019_12372_MOESM3_ESM.pdf]

Description of Additional Supplementary Files

File Name: Supplementary Data 1
Description: Detailed information on all statistical tests performed for Fig 5b,c and Supplementary Figure 20.

File Name: Supplementary Data 2
Description: Detailed information on all statistical tests performed for Supplementary Figure 21.

File Name: Supplementary Software 1
Description: Zip file containing MATLAB and python scripts to process and analyze PRISM images. Software is also available for download at: https://github.com/lcbb/PRISM_analysis
