# Supplementary material for: Multiplexed and high-throughput neuronal fluorescence imaging with diffusible probes
Source: Nat Commun. 2019 Sep 26;10:4377. doi: 10.1038/s41467-019-12372-6 (PMC6763432; doi:10.1038/s41467-019-12372-6)
Supplement: Supplementary file 7 — Reporting Summary [file 41467_2019_12372_MOESM7_ESM.pdf]

nature research

Corresponding author(s): Mark Bathe, Jeffrey Cottrell

Last updated by author(s): Aug 20, 2019

Reporting Summary

Nature Research wishes to improve the reproducibility of the work that we publish. This form provides structure for consistency and transparency in reporting. For further information on Nature Research policies, see Authors & Referees and the Editorial Policy Checklist.

Statistics

For all statistical analyses, confirm that the following items are present in the figure legend, table legend, main text, or Methods section.

n/a	Confirmed	
☐	☒	The exact sample size (*n*) for each experimental group/condition, given as a discrete number and unit of measurement
☐	☒	A statement on whether measurements were taken from distinct samples or whether the same sample was measured repeatedly
☐	☒	The statistical test(s) used AND whether they are one- or two-sided<br>*Only common tests should be described solely by name; describe more complex techniques in the Methods section.*
☒	☐	A description of all covariates tested
☒	☐	A description of any assumptions or corrections, such as tests of normality and adjustment for multiple comparisons
☐	☒	A full description of the statistical parameters including central tendency (e.g. means) or other basic estimates (e.g. regression coefficient) AND variation (e.g. standard deviation) or associated estimates of uncertainty (e.g. confidence intervals)
☐	☒	For null hypothesis testing, the test statistic (e.g. *F*, *t*, *r*) with confidence intervals, effect sizes, degrees of freedom and *P* value noted<br>*Give P values as exact values whenever suitable.*
☒	☐	For Bayesian analysis, information on the choice of priors and Markov chain Monte Carlo settings
☒	☐	For hierarchical and complex designs, identification of the appropriate level for tests and full reporting of outcomes
☐	☒	Estimates of effect sizes (e.g. Cohen's *d*, Pearson's *r*), indicating how they were calculated

Our web collection on statistics for biologists contains articles on many of the points above.

Software and code

Policy information about availability of computer code

Data collection	LNA-PRISM imaging was performed on an Opera Phenix High-Content Screening System (PerkinElmer) using software associated with the machine (Harmony). Single and dual channel PAINT imaging was performed on an inverted Nikon Eclipse Ti microscope (Nikon Instruments). Localization of the center of each diffraction-limited spot corresponding to a single-molecule in the acquired movies was performed using DAOSTORM. All image reconstruction and analysis procedures except for single-molecule localization were performed using MATLAB R2015a .
Data analysis	Custom Matlab scripts, python scripts and the R statistical environment were used to analyze all data. See Methods for details. Links to software will be provided on GitHut.

For manuscripts utilizing custom algorithms or software that are central to the research but not yet described in published literature, software must be made available to editors/reviewers. We strongly encourage code deposition in a community repository (e.g. GitHub). See the Nature Research guidelines for submitting code & software for further information.

Data

Policy information about availability of data

All manuscripts must include a data availability statement. This statement should provide the following information, where applicable:

- Accession codes, unique identifiers, or web links for publicly available datasets
- A list of figures that have associated raw data
- A description of any restrictions on data availability

The full datasets are available from the corresponding author on reasonable request.

Field-specific reporting

Please select the one below that is the best fit for your research. If you are not sure, read the appropriate sections before making your selection.

☒ Life sciences ☐ Behavioural & social sciences ☐ Ecological, evolutionary & environmental sciences

For a reference copy of the document with all sections, see nature.com/documents/nr-reporting-summary-flat.pdf

Life sciences study design

All studies must disclose on these points even when the disclosure is negative.

Sample size	LNA-PRISM (n=3), LNA-PRISM (Synaptic Remodeling) n = 6.
Data exclusions	The Homer-1b/c data was excluded from replicate 6 (TTX) treatment because there was a focal failure error reported by the Phenix machine during that imaging round.
Replication	To account for variabilities within each multi-well plate and across different cultures, imaging was performed on three independent neuronal cultures, and three wells were imaged from each neuronal culture. For the synaptic remodeling experiments, imaging was performed on six wells from two independent rodent neuronal cultures.
Randomization	Not applicable
Blinding	Image acquisition and data analysis were automated and not subject to human bias.

Reporting for specific materials, systems and methods

We require information from authors about some types of materials, experimental systems and methods used in many studies. Here, indicate whether each material, system or method listed is relevant to your study. If you are not sure if a list item applies to your research, read the appropriate section before selecting a response.

Materials & experimental systems

n/a	Involved in the study	
☐	☒	Antibodies
☒	☐	Eukaryotic cell lines
☒	☐	Palaeontology
☐	☐	Animals and other organisms
☒	☐	Human research participants
☒	☐	Clinical data

Methods

n/a	Involved in the study	
☒	☐	ChIP-seq
☒	☐	Flow cytometry
☒	☐	MRI-based neuroimaging

Antibodies

Antibodies used

PSD-95,Cell Signaling Technology,3450,rabbit monoclonal;
bassoon,Enzo Life Sciences,ADI-VAM-PS003,mouse monoclonal;
bassoon,Enzo Life Sciences,ADI-VAM-PS003,mouse monoclonal;
MAP2,Novus Biologicals,NB300-213,chicken polyclonal;
Tuj-1,Sigma,T5076,mouse monoclonal;
phalloidin,Bachem,H-7634,peptide;
ARPC2,Millipore,07-227,rabbit polyclonal;
cortactin,Millipore,05-180,mouse monoclonal;
synapsin-I,Santa Cruz,sc-7379,goat polyclonal;
SHANK3,Santa Cruz,sc-30193,rabbit polyclonal;
Homer-1b/c,Santa Cruz,sc-20807,rabbit polyclonal;
NR2B,NeuroMab,75-097,mouse monoclonal;
anti-rabbit secondary,rabbit IgG,Life Technologies,A16126,goat polyclonal;
anti-mouse secondary,mouse IgG,Life Technologies,A16068,goat polyclonal;
MAP2,Abcam,Ab5392,chicken polyclonal;
VGLUT1,pre-synaptic,Synaptic Systems,135304,guinea pig polyclonal;
Alexa 488 anti-chicken,secondary,chicken IgY,Thermo Fisher,A11039,goat polyclonal;
Alexa 555 anti-guinea pig,secondary,guinea pig IgG,Thermo Fisher,A21435,goat polyclonal;
anti-rat secondaty, goat IgG, Invitrogen, A16126, goat polyclonal;
anti-vGAT,pre-synaptic,Synaptic Systems,131011,mouse monoclonal;
anti-Gephyrin, post-synaptic, Synaptic Systems, 147208,rat monoclonal;

Validation	*Describe the validation of each primary antibody for the species and application, noting any validation statements on the manufacturer's website, relevant citations, antibody profiles in online databases, or data provided in the manuscript.*

Animals and other organisms

Policy information about studies involving animals; ARRIVE guidelines recommended for reporting animal research

Laboratory animals	For rat hippocampal neuronal cultures, E18 embryos were collected from CO2 euthanized pregnant Sprague Dawley rats (Taconic). Hippocampal and cortical mouse neuronal cultures were prepared from postnatal day 0 or day 1 Swiss Webster mice (Taconic).
Wild animals	NA
Field-collected samples	NA
Ethics oversight	Procedures for rat neuronal culture were reviewed and approved for use by the Broad Institutional Animal Care and Use Committee, Procedures for mouse neuronal culture preparation were approved by the Massachusetts Institute of Technology Committee on Animal Care.

Note that full information on the approval of the study protocol must also be provided in the manuscript.
